# Supplementary material for: Effectiveness of a participatory approach to develop school health interventions in four low resource cities: study protocol of the ‘empowering adolescents to lead change using health data’ cluster randomised controlled trial
Source: BMJ Open. 2023 Jul 5;13(7):e071353. doi: 10.1136/bmjopen-2022-071353 (PMC10335517; doi:10.1136/bmjopen-2022-071353)
Supplement: Supplementary data [file bmjopen-2022-071353supp001.pdf]

Appendix

G-SHPPS questionnaire

GSHS questionnaire

Notification, permission and assent forms

Data-to-action workshop agenda

Global School Health Policies and Practices Survey (G-SHPPS)

2021

This questionnaire will be used to assess school health policies and practices across our country. Your cooperation is essential for making the results of this survey comprehensive, accurate, and timely. Your answers will be kept confidential. Individual school results will not be reported.

INSTRUCTIONS

1. This questionnaire should be completed by the principal or headteacher (or the person acting in that capacity) and concerns only activities that occur in this school and local community. Please feel free to consult with other people (such as a health education teacher or school nurse) if you are not sure of an answer.

2. For the purposes of this questionnaire, a "policy" or "school policy" is any **written** law, rule, regulation, administrative order, guideline, standard, or similar kind of mandate issued or created **by this school or a local, state, district, or federal agency or organization with authority over this school**. Schools may sometimes grant policy exceptions or waivers, but please answer each question based on what is considered the general policy and standard practice in this school.

3. For the purposes of this questionnaire, "health" is defined as a state of complete physical, mental, and social well-being and not merely the absence of disease or infirmity.

4. Follow the instructions for each question. If you are unsure about the meaning of a word or phrase that is bolded with an asterisk you will find a definition for it at the end of that question.

5. You may move forward and backward through the questionnaire by using the navigation buttons in the questionnaire. Do not use the forward and backward arrows on your browser.

6. If you need to stop responding and resume later, your responses will be saved. Just press the **Resume later** link at the bottom of the page before closing your browser. To resume please use the same URL or token provided initially to finalize your questionnaire.

7. Once you are comfortable with all your responses, click on the SUBMIT button at the end of the questionnaire.

8. Please answer every question.

9. If you have any questions about this survey, please contact the person identified on the email you were sent about this survey.

THANK YOU FOR YOUR ASSISTANCE.

Table of Contents

	Page
**1. School Overview**	3
**2. School Health Services**	4
School health workers	4
Health services for students	5
Health information management	9
School entry requirements	9
**3. School Physical Environment**	10
Water, sanitation, and hygiene	10
Injury prevention and safety	11
Sun safety	11
Pest control	12
**4. Food and Nutrition Services**	13
Food preparation	14
Other foods and beverages available at school	15
Food and nutrition services environment	16
**5. Health Education**	17
Content of health education	18
**6. Physical Education**	20
Content of physical education	21
Facilities and equipment	21
Physical activity or recreation clubs and competitive sports	22
**7. School Governance and Leadership**	23
School health councils, committees, or teams	23
Community partnerships	24
**8. School Policies and Resources**	25
Overall curriculum	25
Professional development for teachers	26
Bullying and violence prevention	26
Tobacco use prevention	27
Alcohol use prevention	27
Illicit drug use prevention	28
Crisis preparedness and emergency response	28
Eye health	29
Pregnancy students	29

Guthold R, *et al. BMJ Open* 2023; 13:e071353. doi: 10.1136/bmjopen-2022-071353

1. School Overview

1-01. Who attends this school?

A. All genders
B. Only males
C. Only females

1-02. How many students are enrolled in this school? _____

1-03. How many full-time teachers provide instruction to students in this school? _____

1-04. Are the following educational levels taught in this school? *(Mark yes or no for each level.)*

	Yes	No
A. Primary education (**ISCED 1**[1])	O	O
B. Lower secondary education (ISCED 2)	O	O
C. Upper secondary education (ISCED 3)	O	O

1-05. Is this a boarding school where students both learn and live?

A. Yes
B. No

1-06. How would you classify this school?

A. Government or public school
B. Non-government, religious, or private school
C. None of the above

1-07. Is this school located in a mostly rural or mostly urban setting?

A. Mostly rural
B. Mostly urban

1-08. Does this school have internet service reliable enough that it can be routinely used for student instruction?

A. Yes
B. No

[1] A widely-used global reference classification for education systems that is maintained and periodically revised by the UNESCO Institute for Statistics in consultation with Member States and other international and regional organizations. The International Standard for Classification of Education (ISCED) allows comparison of education systems across countries.

2. School Health Services

2-01. Which of the following statements **best** describes how **health services**[2] are regularly provided to students in this school?

A. Health services are provided both on school premises and at separate facilities (not on school premises) through a formal agreement with this school
B. Health services are provided only on school premises
C. Health services are provided only at separate facilities (not on school premises) through a formal agreement with this school to provide health services
D. Health services are only provided on an emergency basis **(Go to 2-15)**
E. I do not know **(Go to 2-15)**

2-02. Which of the following statements best describes how school policy addresses health services for students?

A. School policy provides a specific plan or guidelines for implementing health services for students
B. Health services are mentioned in a school policy, but there is no specific plan or guideline for implementing health services for students
C. Health services for students are not addressed in any school policy
D. I do not know

2-03. Does this school have someone officially responsible for managing or coordinating this school's health services?

A. Yes
B. No
C. I do not know

2-04. Does this school have the supplies needed for teachers and other school staff to apply **standard or universal precautions**[3], including disposable gloves and bandages?

A. Yes
B. No
C. I do not know

School health workers

2-05. Do the following types of **health workers**[4] provide health services to students in this school? *(Mark yes or no or I do not know for each type of health worker.)*

	**Yes**	**No**	**I do not know**
A. Nurses	O	O	O
B. Physicians	O	O	O
C. Psychologists, counsellors, or social workers	O	O	O
D. Dentists or dental hygienists	O	O	O
E. Nutritionists	O	O	O

[2] Services provided by a health worker to students enrolled in primary or secondary education, either on school premises or in a facility not on school premises that has a formal agreement with the school to provide health services to the school's students.
[3] A method of infection control in which all human blood, certain body fluids, and fresh tissues and cells of human origin are handled as if they are known to be infected with Human Immunodeficiency Virus (HIV), Hepatitis B Virus (HBV), and/or other blood-borne pathogens.
[4] A health worker is a person whose main function is to deliver health promotion, prevention, care, and/or treatment services to students, such as a nurse or clinical psychologist, but not a teacher.

Guthold R, *et al. BMJ Open* 2023; 13:e071353. doi: 10.1136/bmjopen-2022-071353

2-06. On average, how many days per week is a health worker available to students in this school?

A. Less than 1 day
B. 1 day
C. 2 days
D. 3 days
E. 4 days
F. 5 or more days
G. I do not know

2-07. Does this school provide professional development opportunities to health workers to improve the quality of health services for students?

A. Yes
B. No
C. I do not know

Health services for students

2-08. As part of the health services offered to students, are the following **preventive interventions**[5] routinely provided? *(Mark yes or no or I do not know for each preventive intervention.)*

	Yes	No	I do not know
A. Routine preventive health check-ups	O	O	O
B. Administration of recommended immunizations (such as **diphtheria, HPV, tetanus, measles, and rubella**[6])	O	O	O
C. **Micronutrient**[7] supplements (such as iron, iodine, zinc, or Vitamin A)	O	O	O

2-09. Does this school provide information to students, parents, caregivers, and families about the value and importance of receiving routine immunizations to prevent infectious diseases?

A. Yes
B. No
C. I do not know

2-10. As part of the health services offered to students, are the following **screenings**[8] routinely provided for at least most students? *(Mark yes or no or I do not know for each screening.)*

	Yes	No	I do not know
A. Eye and vision	O	O	O
B. Ear and hearing	O	O	O
C. Oral health	O	O	O
D. Nutrition (such as for **anaemia, malnutrition, and obesity**[9])	O	O	O

Note: If A, B, C, and D are all No or I do not know – Go to 2-12.

[5] A health intervention to prevent illness, disease, or injury.
[6] Diphtheria – A serious bacterial infection of the nose and throat. HPV – A sexually transmitted infection caused by the human papillomavirus that may lead to genital warts or cancer. Tetanus – A bacterial infection that causes painful muscle contractions particularly in the jaw and neck. Measles – A highly contagious viral disease characterized by a high fever and rash. Rubella – A viral disease characterized by a low fever, sore throat, and rash.
[7] Vitamins and minerals vital to healthy development, disease prevention, and well-being.
[8] Medical tests to check for diseases and health conditions before there are any signs or symptoms.
[9] Anaemia – A condition marked by a lack of red blood cells or of hemoglobin in the blood. Malnutrition – Lack of proper nutrition caused by not having enough to eat, not eating enough of the right things, or another disease. Obesity – A disorder involving excess body fat that increases the risk of other health problems.

Guthold R, *et al. BMJ Open* 2023; 13:e071353. doi: 10.1136/bmjopen-2022-071353

2-11. Do the following actions typically occur when a student's screening indicates a potential problem? *(Mark yes or no or I do not know for each action.)*

	Yes	No	I do not know
A. The student is notified	O	O	O
B. The student's parents or caregiver are notified	O	O	O
C. The student's teachers are notified	O	O	O
D. A referral is provided for an appropriate health worker	O	O	O

2-12. As part of the health services offered to students, are the following **health promotion**[10] activities routinely provided? *(Mark yes or no or I do not know for each health promotion activity.)*

	Yes	No	I do not know
A. Timely care-seeking from an appropriate provider	O	O	O
B. Increased health literacy	O	O	O
C. Improved personal hygiene and handwashing with soap	O	O	O
D. Improved oral health care	O	O	O
E. Reduced consumption of sugar and **sugar-sweetened**[11] beverages	O	O	O
F. Increased physical activity and limited **sedentary behaviour**[12]	O	O	O
G. Appropriate use of electronic devices	O	O	O
H. Attainment of adequate sleep	O	O	O
I. Appropriate sun exposure for the context	O	O	O
J. Menstrual hygiene management	O	O	O

Note: Ask J only if females attend this school.

[10] The process of enabling individuals to increase control over, and to improve, their health. Health promotion can happen formally or informally, in a group or one-on-one, and in a clinical setting.
[11] Sugar-sweetened beverages include carbonated soft drinks, sports drinks, energy drinks,100% fruit juices, fruit drinks that are not 100% juice, sugar-sweetened flavoured milks, and sugar-sweetened teas, coffees, or flavoured waters.
[12] Activities occurring in a reclining, seated, or lying position requiring very low energy expenditure.

2-13. As part of the health services offered to students, are the following health services in each health area routinely provided to students? Health services may include a clinical assessment and subsequent care and support or referral to another facility for care. *(Mark yes or no or I do not know for each health service.)*

	Yes	No	I do not know
**General/cross cutting**			
A. Provision of first aid	O	O	O
B. Administration of over-the-counter and prescribed medications	O	O	O
C. Control and management of pain (such as from a headache or toothache)	O	O	O
D. Management of non-specific symptoms (such as diarrhoea or fever)	O	O	O
**Positive health development**			
E. Identification of developmental difficulties and disabilities	O	O	O
F. Counselling related to physical and psychosocial development (such as puberty, skin changes, body image, or child marriage)	O	O	O
**Unintentional injury**			
G. Management of common childhood injuries (such as fractures or wounds)	O	O	O
H. Management of burns	O	O	O
I. Management of non-fatal drowning and related complications	O	O	O
**Violence**			
J. Counselling to prevent violence (including intimate partner violence, sexual violence, gender-based violence, bullying, and gang violence)	O	O	O
K. Support for victims of violence	O	O	O
**Sexual and reproductive health, including HIV**			
L. Contraceptive counselling	O	O	O
M. Counselling on prevention of HIV or sexually-transmitted infections	O	O	O
N. **HIV pre-exposure and/or post-exposure prophylaxis**[13]	O	O	O
O. HIV testing services	O	O	O
P. Management of sexually-transmitted infections	O	O	O
Q. Management of pregnancy (including the option to continue or return to school)	O	O	O

Note: Ask Q only if females attend this school.

[13] Medicine taken to prevent getting HIV either before or after high risk behaviour.

2-14. As part of the health services offered to students, are the following health services in each health area routinely provided to students? Health services may include a clinical assessment and subsequent care and support or referral to another facility for care. *(Mark yes or no or I do not know for each health service.)*

	Yes	No	I do not know
**Communicable disease**			
A. Management of common infections (such as ear, eye, dental, skin, throat, or urinary tract infections)	O	O	O
B. Management of other infectious diseases (such as **cholera, dengue, dysentery, helminths, tuberculosis, or malaria**[14*])	O	O	O
C. Support for chronic care of HIV-infected children	O	O	O
**Noncommunicable disease, physical disability, and nutrition**			
D. Management of **anaemia**[15] (such as iron supplementation)	O	O	O
E. Support for management of overweight and **obesity**[16]	O	O	O
F. Management of asthma	O	O	O
G. Management of other chronic conditions (such as developmental disabilities, diabetes, heart disease, or seizures)	O	O	O
H. Rehabilitation, assistive technology, and other services for injured or disabled children	O	O	O
**Mental health, substance use, and self-harm**			
I. Assessment of risk factors and health behaviours (such as tobacco use, drug use, or physical inactivity)	O	O	O
J. Counselling on tobacco, alcohol, and other substance use	O	O	O
K. Short-term counselling or crisis intervention focused on mental health or situational concerns (such as grief or difficult transitions)	O	O	O
L. Management of common behavioural disorders in children (such as ADHD)	O	O	O
M. Management of emotional, anxiety, and depressive disorders	O	O	O
N. Management of eating disorders (such as **anorexia or bulimia**[17])	O	O	O
O. Stress management	O	O	O
P. Management of self-harm and/or suicide risk	O	O	O
Q. Management of **somatoform disorders**[18] and other psychosomatic conditions	O	O	O
R. Management of psychotic disorders	O	O	O
S. Management of harmful use of, dependence on, or withdrawal from a substance (such as alcohol or illicit drugs)	O	O	O

[14] Cholera – A bacterial disease causing acute diarrhoea and dehydration. Dengue – A viral disease spread through the bite of a certain type of infected mosquito. Dysentery – A bacterial disease causing inflammation of the intestines and bloody diarrhoea. Helminths – A parasitic worm that causes disease. Tuberculosis – A bacterial infection that mainly effects the lungs. Malaria – A serious and sometimes fatal disease caused by a parasite that infects a certain type of mosquito that feeds on humans.

[15] Anaemia – A condition marked by a lack of red blood cells or of hemoglobin in the blood.

[16] Obesity – A disorder involving excess body fat that increases the risk of other health problems.

[17] Anorexia – An eating disorder characterized by a very low body weight, an intense fear of gaining weight, and a distorted perception of weight. Bulimia - An eating disorder marked by binging followed by methods to avoid weight gain.

[18] Physical symptoms that suggest illness or injury, but which cannot be explained fully by a general medical condition or by the direct effect of a substance.

Health information management

2-15. Are the following types of health information recorded or kept on file in hard copy or electronic format for students in this school? *(Mark yes or no or I do not know for each type of health information.)*

	Yes	No	I do not know
A. Emergency contact information	O	O	O
B. Physical health history	O	O	O
C. Mental health history	O	O	O
D. Screening results	O	O	O
E. Vaccination history	O	O	O
F. Food allergy or other allergy information	O	O	O
G. Disabilities or special learning needs	O	O	O

2-16. Are the following types of data routinely collected from all students that attend this school? *(Mark yes or no or I do not know for each type of data.)*

	Yes	No	I do not know
A. Student health risk behaviours	O	O	O
B. Student injuries or illnesses that occur at school	O	O	O
C. Student use of school health services	O	O	O
D. Student perception of the quality of school health services	O	O	O
E. Reasons why students are absent from school	O	O	O

Note: If A, B, C, D, and E are all No or I do not know - Go to 2-18.

2-17. Does this school have an established process for routinely reviewing these data collected from students to help develop and implement school policies, programs, or activities?

A. Yes
B. No
C. I do not know

2-18. Does this school report any **notifiable diseases**[19] or infectious disease outbreaks that occur at school among students to the federal, state, or local ministry of health?

A. Yes
B. No
C. I do not know

School entry requirements

2-19. Does this school have a policy requiring students to be in compliance with the **National Immunization Schedule**[20] prior to enrollment in this school?

A. Yes
B. No
C. Not applicable. This country does not have a National Immunization Schedule.
D. I do not know

2-20. Does this school require students to have a preventive health check-up prior to enrollment in this school?

A. Yes
B. No
C. I do not know

[19] A notifiable disease is any disease that is required by law to be reported to government authorities.
[20] A National Immunization Schedule is a list of vaccinations, including the timing of all doses, which may be either recommended or compulsory, depending on the country

3. School Physical Environment

Water, sanitation, and hygiene

3-01. Which of the following statements **best** describes the **current** service level of this school's handwashing facilities for students?

A. Soap and water are available at handwashing facilities for students
B. Only water, but no soap, is available at handwashing facilities for students
C. No water is available at handwashing facilities for students
D. No handwashing facilities for students are available
E. I do not know

3-02. Which of the following statements **best** describes the **current** service level of this school's drinking water for students?

A. Drinking water for students is available from an **improved source**[21]
B. Drinking water for students comes from an improved source, but is not available
C. Drinking water for students is available from an **unimproved source**[22]
D. No drinking water for students is available
E. I do not know

3-03. Which of the following statements **best** describes the **current** service level of this school's sanitation facilities (such as toilets or latrines) for students?

A. Students have access to **improved sanitation facilities**[23] that are single sex, functional, and private
B. Students have access to improved sanitation facilities, but they are not single sex, they are not functional, or they are not private
C. Students only have access to **unimproved sanitation facilities**[24]
D. No sanitation facilities for students are available **(Go to 3-06)**
E. I do not know **(Go to 3-06)**

3-04. Does this school have sanitation facilities (such as toilets or latrines) that are accessible to students with physical disabilities?

A. Yes
B. No
C. I do not know

3-05. Are the school's sanitation facilities (such as toilets or latrines) usually cleaned daily when this school is in session?

A. Yes
B. No
C. I do not know

---

[21] Improved drinking water sources includes sources that, by nature of their construction or through active intervention, are protected from outside contamination, particularly fecal matter. These include piped water in a dwelling, plot, or yard; public standpipe; borehole; protected dug well; protected spring; and rainwater collection.
[22] Unimproved drinking water sources include unprotected dug well, unprotected spring, cart with small tank/drum, tanker truck, and surface water (river, dam, lake, pond, stream, canal, and irrigation channels).
[23] Improved sanitation facilities include a connection to a public sewer system, connection to a septic system, pour-flush latrine, and a ventilated improved pit latrine.
[24] Unimproved sanitation facilities include a public or shared latrine, an open pit latrine, and a bucket latrine.

3-06. Is garbage usually removed daily from the school premises when this school is in session?

A. Yes
B. No
C. I do not know

3-07. Does this school provide sanitary napkins or other menstrual supplies to students?

A. Yes, for free
B. Yes, for a fee
C. No
D. I do not know

Note: Ask this question only if females attend this school.

Injury prevention and safety

3-08. Are the following places or types of equipment routinely inspected for safety issues and hazards? *(Mark yes or no or I do not know for each place or type of equipment or mark not applicable (N/A) if this school does not have the place or type of equipment.)*

	Yes	No	I do not know	N/A
A. Playground equipment	O	O	O	O
B. Sports facilities	O	O	O	O
C. Fire extinguishers or other equipment to extinguish a fire (such as buckets of sand)	O	O	O	O
D. School structures and buildings	O	O	O	O
E. School grounds	O	O	O	O
F. Water source	O	O	O	O

3-09. Does this school have the following features to promote safety among students, including students with disabilities? *(Mark yes or no or I do not know for each feature.)*

	Yes	No	I do not know
A. Handrails on stairs	O	O	O
B. Ramps to facilitate change of level	O	O	O
C. Assistive devices (such as wheelchairs, wider doorways, specialized software, or adaptive switches or knobs)	O	O	O
D. Signage or paint to address vision impairments	O	O	O

Sun safety

3-10. Does this school use the following strategies to reduce sun exposure among students? *(Mark yes or no or I do not know for each strategy.)*

	Yes	No	I do not know
A. Schedule outdoor activities to avoid peak sun intensity	O	O	O
B. Provide shade areas	O	O	O
C. Encourage students to wear hats, other head coverings, or protective clothing when they are outside	O	O	O
D. Encourage students to wear sunscreen or other skin protection products when they are outside	O	O	O

Guthold R, *et al. BMJ Open* 2023; 13:e071353. doi: 10.1136/bmjopen-2022-071353

Pest control

3-11. Are the school grounds where students play or exercise surrounded by a fence to keep out stray or roaming animals?

A. Yes
B. No
C. Not applicable. Stray or roaming animals are not a problem on school grounds
D. I do not know

3-12. Are procedures routinely implemented to control common pests (such as rodents, insects, or snakes) on school grounds?

A. Yes
B. No
C. I do not know

4. Food and Nutrition Services

4-01. Does this school provide **food and nutrition services**[25] to students?

A. Yes
B. No **(Go to 4-13)**
C. I do not know **(Go to 4-13)**

4-02. Which of the following statements **best** describes how school policy addresses food and nutrition services for students?

A. School policy provides a specific plan or guidelines for implementing food and nutrition services for students
B. Food and nutrition services are mentioned in a school policy, but there is no specific plan or guideline for implementing food and nutrition services for students
C. Food and nutrition services for students are not addressed in any school policy
D. I do not know

4-03. Does this school have someone officially responsible for managing or coordinating this school's food and nutrition services?

A. Yes
B. No
C. I do not know

4-04. Are the following meals or snacks routinely made available to students as part of this school's food and nutrition services? *(Mark yes or no or I do not know for each meal or snack.)*

	Yes	No	I do not know
A. Breakfast	O	O	O
B. Lunch	O	O	O
C. Evening meal	O	O	O
D. Snack(s)	O	O	O
E. Take-home rations	O	O	O

4-05. Which of the following statements **best** describes which students receive free or deeply discounted food as part of this school's food and nutrition services?

A. All students receive free or deeply discounted food
B. Some students receive free or deeply discounted food as determined by some kind of **social means testing**[26]* or other quantitative criteria
C. No students receive free or deeply discounted food
D. I do not know

4-06. Does this school routinely conduct the following activities as part of its food and nutrition services for students? *(Mark yes or no or I do not know for each activity.)*

	Yes	No	I do not know
A. Collect suggestions from students, families, teachers, or other school staff on nutritious food and beverage preferences	O	O	O
B. Provide information to students or families on the nutrition and caloric content of foods and beverages	O	O	O
C. Work with local businesses to enhance the quality or variety of foods offered as part of this school's food and nutrition services	O	O	O

[25] The provision of food support either in the form of actual food or cash/vouchers/stipends for students to buy food available on school grounds.
[26] Assessment using set criteria to identify students who should receive school meals at no cost or at a deeply discounted price.

4-07. Are the following foods and beverages routinely made available to students through the school's food and nutrition services. *(Mark yes or no or I do not know for each food or beverage.)*

	Yes	No	I do not know
A. Fruit	O	O	O
B. Vegetables	O	O	O
C. Milk or milk products	O	O	O
D. Foods high in fiber such as whole grains, legumes, or nuts	O	O	O
E. Foods high in sugar including cookies, pastries, or other baked goods	O	O	O
F. Salty foods such as chips or crackers	O	O	O
G. Chocolate or other candy	O	O	O
H. Fried foods or other foods high in unhealthy fats	O	O	O
I. Sugar-sweetened carbonated soft drinks	O	O	O
J. Sports and energy drinks	O	O	O
K. 100% fruit or vegetable juices	O	O	O
L. Fruit or vegetable drinks that are not 100% juice	O	O	O
M. Sugar-sweetened flavoured milks	O	O	O
N. Sugar-sweetened teas, coffees, or flavoured waters	O	O	O
O. Water	O	O	O

Food preparation

4-08. Does this school have a policy describing how food served as part of the food and nutrition services should be prepared (in the school or elsewhere) to maximize its nutritional quality?

A. Yes
B. No **(Go to 4-13)**
C. I do not know **(Go to 4-13)**

4-09. Are the following nutrition objectives specifically addressed by this policy? *(Mark yes or no or I do not know for each nutrition objective.)*

	Yes	No	I do not know
A. Reduce saturated or trans-fat intake	O	O	O
B. Reduce salt intake	O	O	O
C. Reduce sugar intake	O	O	O
D. Increase fruit and vegetable consumption	O	O	O
E. Increase consumption of whole grains, legumes, and nuts	O	O	O
F. Increase **micronutrient**[27] intake	O	O	O

4-10. Are any foods served as part of this school's food and nutrition services fortified with **micronutrients**[27] (such as iron, iodine, zinc, or Vitamin A)?

A. Yes
B. No
C. I do not know

4-11. Are the foods that are part of this school's food and nutrition services prepared in the following locations? *(Mark yes or no or I do not know for each location.)*

	Yes	No	I do not know
A. On school grounds	O	O	O
B. Off-site in a centralized (not private) facility	O	O	O
C. Off-site in a private facility, such as a caterer	O	O	O

[27] Vitamins and minerals vital to healthy development, disease prevention, and well-being.

4-12. Does this school have a policy requiring food preparation staff (on school grounds or off-site) to follow safe food handling practices, such as **WHO's 5 Keys to Safer Food**[28]?

A. Yes
B. No
C. I do not know

Other foods and beverages available at school

4-13. May students purchase food or beverages from a vending machine, school store, canteen, or snack bar on school premises?

A. Yes
B. No **(Go to 4-17)**
C. I do not know **(Go to 4-17)**

4-14. Are healthy foods and beverages in the vending machine, school store, canteen, or snack bar on school premises priced lower than unhealthy options to encourage students to purchase the healthy ones?

A. Yes
B. No
C. I do not know

4-15. Does this school have a policy banning the sale of **sugar-sweetened beverages**[29] to students?

A. Yes
B. No
C. I do not know

4-16. Are the following foods and beverages routinely made available to students through the vending machines, stores, canteens, or snack bars on school premises. *(Mark yes or no or I do not know for each food or beverage.)*

	Yes	No	I do not know
A. Fruit	O	O	O
B. Vegetables	O	O	O
C. Milk or milk products	O	O	O
D. Foods high in fiber such as whole grains, legumes, or nuts	O	O	O
E. Foods high in sugar including cookies, pastries, or other baked goods	O	O	O
F. Salty foods such as chips or crackers	O	O	O
G. Chocolate or other candy	O	O	O
H. Fried foods or other foods high in unhealthy fats	O	O	O
I. Sugar-sweetened carbonated soft drinks	O	O	O
J. Sports and energy drinks	O	O	O
K. 100% fruit or vegetable juices	O	O	O
L. Fruit or vegetable drinks that are not 100% juice	O	O	O
M. Sugar-sweetened flavoured milks	O	O	O
N. Sugar-sweetened teas, coffees, or flavoured waters	O	O	O
O. Water	O	O	O

[28] WHO's 5 Keys to Safer Food are clean, separate raw and cooked, cook thoroughly, keep food at safe temperatures, and use safe water and raw materials.
[29] Sugar-sweetened beverages include carbonated soft drinks, sports drinks, energy drinks, 100% fruit juices, fruit drinks that are not 100% juice, sugar-sweetened flavoured milks, and sugar-sweetened teas, coffees, or flavoured waters.

Food and nutrition services environment

4-17. Does this school have a policy that students may bring food from home to eat during the school day?

A. Yes
B. No
C. I do not know

4-18. Does this school have a policy that students may bring water from home to drink during the school day?

A. Yes
B. No
C. I do not know

4-19. Where do most students **usually** eat meals during the school day?

A. In their regular classroom
B. In a dedicated eating space, such as a school cafeteria, dining hall, or other special room besides their regular classroom
C. Outside on school grounds
D. Somewhere else
E. Students do not eat meals during the school day
F. I do not know

4-20. Does this school have a policy prohibiting advertisements or promotions, including sponsorships, for candy, **sugar-sweetened beverages**[30], or other unhealthy foods and beverages on school premises?

A. Yes
B. No
C. I do not know

[30] Sugar-sweetened beverages include carbonated soft drinks, sports drinks, energy drinks, 100% fruit juices, fruit drinks that are not 100% juice, sugar-sweetened flavoured milks, and sugar-sweetened teas, coffees, or flavoured waters.

Guthold R, *et al. BMJ Open* 2023; 13:e071353. doi: 10.1136/bmjopen-2022-071353

5. Health Education

5-01. Does this school teach **health education**[31]?

A. Yes
B. No **(Go to 6-01)**
C. I do not know **(Go to 6-01)**

5-02. Which of the following statements **best** describes how health education instruction occurs in this school?

A. Health education instruction occurs only in a regular classroom setting
B. Health education instruction occurs only through extra-curricular activities (such as after school, during lunch, or via clubs or school assemblies)
C. Health education instruction occurs both in a regular classroom setting and through extra-curricular activities
D. I do not know

5-03. Do students receive a grade for health education?

A. Yes
B. No
C. I do not know

5-04. Does this school have someone officially responsible for managing or coordinating this school's health education?

A. Yes
B. No
C. I do not know

5-05. Who provides **most** of the health education instruction in this school?

A. A health education teacher or specialist
B. A nurse or some other kind of health worker
C. Someone else
D. I do not know

5-06. Are the following materials provided to teachers in this school to guide health education instruction? *(Mark yes or no or I do not know for each material.)*

	**Yes**	**No**	**I do not know**
A. Learning outcomes or objectives	O	O	O
B. A planned progression of lesson plans or learning strategies and experiences	O	O	O
C. Teaching and learning resources and content	O	O	O
D. Assessment tools	O	O	O

[31] Any combination of learning experiences designed to help students improve their health by increasing their knowledge, influencing motivation, and improving health literacy.

5-07. Which of the following statements **best** describes how school policy addresses teaching **skills-based, participatory health education**[32]?

A. School policy provides a specific plan or guidance on teaching skills-based participatory health education
B. School policy mentions health education, but not teaching skills-based participatory health education
C. School policy does not mention health education
D. I do not know

5-08. Does this school routinely provide professional development opportunities for teachers to improve the quality of health education instruction?

A. Yes
B. No
C. I do not know

Content of health education

5-09. Are the following skills taught to students in this school to help them avoid or reduce health risks? *(Mark yes or no or I do not know for each skill.)*

	**Yes**	**No**	**I do not know**
A. How to access high quality health information, products, or services	O	O	O
B. Interpersonal communication	O	O	O
C. Decision-making	O	O	O
D. Problem-solving	O	O	O
E. Goal-setting	O	O	O
F. Refusal	O	O	O
F. Coping or stress management	O	O	O
G. Hand washing with soap	O	O	O
H. Tooth brushing with fluoride toothpaste	O	O	O
I. Using the internet and social media safely	O	O	O
J. Advocating for personal, family, or community health and wellbeing	O	O	O

[32] Skills-based, participatory health education includes strategies and techniques focused on skill development that encourage students to become actively involved in their learning process.

5-10. Are the following health topics taught to students in this school? *(Mark yes or no or I do not know for each topic.)*

	Topic	Yes	No	I do not know
A.	Physical activity and fitness	O	O	O
B.	Nutrition and dietary behaviour	O	O	O
C.	**Sexual and reproductive health**[33]	O	O	O
D.	HIV transmission, prevention, and treatment	O	O	O
E.	Road safety	O	O	O
F.	Violence and bullying prevention	O	O	O
G.	Suicide prevention	O	O	O
H.	Emotional and mental health	O	O	O
I.	Tobacco and nicotine use prevention	O	O	O
J.	Alcohol use prevention	O	O	O
K.	Illicit drug use prevention	O	O	O
L.	Infectious disease (such as the cold, flu, or COVID-19) prevention	O	O	O
M.	Non-communicable disease (such as diabetes, cancer, or obesity) prevention	O	O	O
N.	Oral health and prevention of oral disease	O	O	O
O.	Healthy sleep	O	O	O
P.	Personal hygiene, including handwashing	O	O	O
Q.	Environmental health (such as the importance of clean air or water)	O	O	O
R.	Sustainable development and consumption	O	O	O
S.	Natural disaster preparedness and response	O	O	O
T.	First aid	O	O	O
U.	Immunizations (such as the HPV or COVID-19 vaccinations)	O	O	O
V.	Safe sun exposure	O	O	O
W.	Equity, inclusion, diversity, and human rights	O	O	O
X.	Gender inequality and social norms	O	O	O

[33] Includes instruction on human growth and development, family life, reproduction, condoms and contraception, pregnancy, sexual behaviour, sexual abuse, and transmission and prevention of sexually transmitted infections.

6. Physical Education

Note: Ask this question if only males or only females attend this school.
6-01. Does this school teach **physical education**[34]?

A. Yes
B. No **(Go to 6-16)**
C. I do not know **(Go to 6-16)**

Note: Ask this question if both males and females attend this school.
6-02. Who is taught **physical education**[34] in this school?

A. All genders
B. Only males
C. Only females
D. No one **(Go to 6-16)**
E. I do not know **(Go to 6-16)**

6-03. Does this school provide adapted physical education for students with disabilities?

A. Yes
B. No
C. I do not know

6-04. Do students receive a grade for physical education?

A. Yes
B. No
C. I do not know

6-05. Can students in this school be excused from physical education for an extended period for the following reasons? *(Mark yes or no or I do not know for each reason.)*

	**Yes**	**No**	**I do not know**
A. Cultural	O	O	O
B. Gender	O	O	O
C. Academic achievement	O	O	O
D. Academic struggles or failure	O	O	O
E. Participation in other school activities	O	O	O
F. Poor physical health	O	O	O
G. Menstruation	O	O	O

Note: Ask G only if females attend this school.

6-06. Who provides **most** of the physical education instruction in this school?

A. A physical education teacher or specialist
B. A nurse or some other kind of health worker
C. Someone else
D. I do not know

[34] Class time spent teaching a physical education curriculum. Does not include instruction on physical activity topics in health education or any other subject.

6-07. Are the following materials provided to teachers in this school to guide physical education? *(Mark yes or no or I do not know for each type of material.)*

	Yes	No	I do not know
A. Learning outcomes or objectives	O	O	O
B. A planned progression of lesson plans or learning strategies and experiences	O	O	O
C. Teaching and learning resources and content	O	O	O
D. Assessment tools	O	O	O

6-08. Which of the following statements **best** describes how school policy addresses physical education for students?

A. School policy provides a specific plan or guidelines for implementing physical education
B. Physical education is mentioned in a school policy, but there is no specific plan or guideline for implementing physical education
C. Physical education is not addressed in any school policy
D. I do not know

6-09. Does this school routinely provide professional development opportunities for teachers to improve the quality of physical education instruction?

A. Yes
B. No
C. I do not know

Content of physical education

6-10. Are the following topics taught to students in this school? *(Mark yes or no or I do not know for each topic.)*

	Yes	No	I do not know
A. Movement concepts and skills (including motor skills such as walking or skipping and manipulative skills such as throwing, catching, or kicking)	O	O	O
B. The importance of life-long participation in physical activity	O	O	O
C. Developing an individualized physical activity plan, including frequency, intensity, and duration	O	O	O
D. The value and importance of fair play	O	O	O
E. Preventing injury during physical activity	O	O	O
F. The value of physical activity for health, enjoyment, challenge, self-expression, and/or social interaction	O	O	O
G. Strength training	O	O	O
H. Group or team sports or activities	O	O	O
I. Individual or paired sports or activities	O	O	O
J. The importance of endurance (aerobic) exercise for overall health and fitness	O	O	O
K. Flexibility or range of movement	O	O	O

Facilities and equipment

6-11. Does this school have a safe and clean **indoor** space for physical education class?

A. Yes
B. No
C. I do not know

6-12. Does this school have a safe and clean **outdoor** space for physical education class?

A. Yes
B. No **(Go to 6-16)**
C. I do not know **(Go to 6-16)**

6-13. Does this school allow community members to access this outdoor space for physical activity when school is not in session?

A. Yes
B. No
C. I do not know

6-14. Does this school provide a place where males and females can separately and privately change clothes before and after physical education?

A. Yes
B. No
C. I do not know

Note: Ask this question if both males and females attend this school.

6-15. Does this school have equipment (such as skipping ropes or balls) for use during physical education?

A. Yes
B. No
C. I do not know

Physical activity or recreation clubs and competitive sports

6-16. Does this school regularly provide recess or other physical activity breaks to students during the school day?

A. Yes
B. No
C. I do not know

6-17. Does this school offer **non-competitive physical activity or recreation clubs**[35] for students?

A. Yes
B. No
C. I do not know

6-18. Does this school offer school-sponsored sports teams that compete against teams from other schools?

A. Yes, for males only
B. Yes, for females only
C. Yes, for all genders
D. No
E. I do not know

Note: Ask this question if both males and females attend this school.

6-19. Does this school offer school-sponsored sports teams that compete against teams from other schools?

A. Yes
B. No
C. I do not know

Note: Ask this question if only males or only females attend this school.

[35] Any non-competitive physical activity program that is voluntary for students, in which students are given an equal opportunity to participate regardless of physical ability, and in which students have the opportunity to be involved in the planning, organization, and administration of the program, under the supervision of a qualified adult.

7. School Governance and Leadership

School health councils, committees, or teams

7-01. Does this school have an official council, committee, or team responsible for implementing health promoting school policies, programs, and activities?

A. Yes
B. No **(Go to 7-05)**
C. I do not know **(Go to 7-05)**

7-02. How often each year does this council, committee, or team routinely meet to address health promoting school policies, programs, and activities?

A. Weekly or more often
B. Monthly
C. Quarterly
D. Twice a year
E. Once a year or less often
F. I do not know

7-03. Are the following groups formally represented on this council, committee, or team? *(Mark yes or no or I do not know for each group.)*

	Yes	No	I do not know
A. School administrators	O	O	O
B. Teachers	O	O	O
C. Other school staff	O	O	O
D. Students	O	O	O
E. Parents, caregivers, or families	O	O	O
F. Government officials	O	O	O
G. Local businesses	O	O	O
H. Health workers (such as doctors or nurses)	O	O	O
I. Religious leaders	O	O	O

7-04. Does this council, committee, or team routinely conduct the following activities? *(Mark yes or no or I do not know for each activity.)*

	Yes	No	I do not know
A. Identify student health needs based on a review of relevant data	O	O	O
B. Recommend new or revised health promoting policies, programs, or activities to school administrators	O	O	O
C. Seek funding or leverage resources to support health promoting policies, programs, or activities for students or school staff	O	O	O
D. Communicate the importance of health promoting policies, programs, or activities to the school or community	O	O	O
E. Review health-related curricula or instructional materials	O	O	O

Guthold R, *et al. BMJ Open* 2023; 13:e071353. doi: 10.1136/bmjopen-2022-071353

Community partnerships

7-05. Are the following groups regularly involved and engaged in **developing** health promoting school policies? *(Mark yes or no or I do not know for each group.)*

	Yes	No	I do not know
A. School administrators	O	O	O
B. Teachers	O	O	O
C. Other school staff	O	O	O
D. Students	O	O	O
E. Parents, caregivers, or families	O	O	O
F. Government officials	O	O	O
G. Local businesses	O	O	O
H. Health workers (such as doctors or nurses)	O	O	O
I. Religious leaders	O	O	O

7-06. Do the following groups regularly **receive copies of or information about** updated or new health promoting school policies? *(Mark yes or no or I do not know for each group.)*

	Yes	No	I do not know
A. School administrators	O	O	O
B. Teachers	O	O	O
C. Other school staff	O	O	O
D. Students	O	O	O
E. Parents, caregivers, or families	O	O	O
F. Government officials	O	O	O
G. Local businesses	O	O	O
H. Health workers (such as doctors or nurses)	O	O	O
I. Religious leaders	O	O	O

Guthold R, *et al. BMJ Open* 2023; 13:e071353. doi: 10.1136/bmjopen-2022-071353

8. School Policies and Resources

8-01. Which of the following statements **best** describes how overall student health is promoted in school policy?

A. School policy provides a specific plan or guidance for promoting overall student health
B. Overall student health is mentioned in school policy, but without a specific plan or guidance on how to promote it
C. Overall student health is not addressed in any school policy
D. I do not know

8-02. Which of the following statements **best** describes the availability of resources in this school's budget to improve overall student health?

A. The school budget contains adequate resources to improve overall student health
B. The school budget contains some resources for improvement of overall student health, but not enough to do what is needed
C. The school budget does not contain any resources for improvement of overall student health
D. I do not know

8-03. Which of the following statements **best** describes how the health of teachers and other school staff is promoted in school policy?

A. School policy provides a specific plan or guidance for promoting the health of teachers and other school staff
B. The health of teachers and other school staff is mentioned in school policy, but without a specific plan or guidance on how to promote it
C. The health of teachers and other school staff is not addressed in any school policy
D. I do not know

8-04. Does this school regularly monitor, evaluate, or assess the quality of its health promoting policies, programs, or activities?

A. Yes
B. No
C. I do not know

The concept of a Health Promoting School embodies a whole-school approach to promoting health and educational attainment by capitalizing on the organizational potential of schools to foster the physical, social–emotional, and psychological conditions for health and positive educational outcomes. A Health Promoting School constantly strengthens its capacity as a safe and healthy setting for living, learning, and working. Other terms used to describe Health Promoting Schools are "comprehensive school health," "healthy school communities," and "school health education."

8-05. Does this school have a policy specifically about becoming or continuing to be a Health Promoting School?

A. Yes
B. No
C. I do not know

Overall curriculum

8-06. Does this school support **skills-based, participatory teaching methods**[36]?

A. Yes
B. No
C. I do not know

[36] Skills-based, participatory teaching methods are strategies and techniques focused on skill development that encourage students to become actively involved in their learning process.

8-07. Does this school allow teachers to make curriculum adaptations as appropriate to address the learning needs of students with disabilities?

A. Yes
B. No
C. I do not know

Professional development for teachers

8-08. Does this school routinely provide professional development opportunities for teachers on the following topics? *(Mark yes or no or I do not know for each topic.)*

	Yes	No	I do not know
A. The link between health and learning	O	O	O
B. Skills-based, participatory teaching methods	O	O	O
C. **Positive classroom management techniques**[37]	O	O	O
D. How to be a health promoting school	O	O	O
E. How to assess the health-related needs of students	O	O	O
F. How to deliver health-related content that best meets the needs of students	O	O	O
G. Standards for a healthy and safe learning environment	O	O	O
H. Child and adolescent development	O	O	O

Bullying and violence prevention

8-09. Does this school have a policy specifically prohibiting the following types of violence? *(Mark yes or no or I do not know for each type of violence.)*

	Yes	No	I do not know
A. Bullying among students	O	O	O
B. Fighting among students	O	O	O
C. Corporal punishment of students by teachers or other school staff	O	O	O
D. Physical, emotional, or sexual abuse of students by teachers or other school staff	O	O	O

8-10. Does this school have specific procedures in place for how a student can safely report any type of violence (such as bullying; fighting; corporal punishment; or physical, emotional, or sexual abuse)?

A. Yes
B. No
C. I do not know

8-11. Does this school have a policy describing how to respond when any type of violence (such as bullying; fighting; corporal punishment; or physical, emotional, or sexual abuse) occurs?

A. Yes
B. No
C. I do not know

[37] Positive classroom management techniques are focused on supporting and involving students to help them learn rather than focusing on bad behaviour.

8-12. Does this school collect data about incidences of violence (such as bullying; fighting; corporal punishment; or physical, emotional, or sexual abuse) including when, where, or how it occurred or who was involved?

A. Yes
B. No
C. I do not know

Tobacco use prevention

8-13. Does this school have a policy prohibiting use of at least some tobacco or nicotine products (such as cigarettes, other forms of smoked tobacco products, smokeless tobacco products, or electronic cigarettes) among the following groups on school premises? *(Mark yes or no or I do not know for each group.)*

	Yes	No	I do not know
A. Students	O	O	O
B. Teachers and other school staff	O	O	O
C. Visitors to the school	O	O	O

8-14. Does this school take the following actions to help reduce use of tobacco or nicotine products among students? *(Mark yes or no or I do not know for each action.)*

	Yes	No	I do not know
A. Implement policies on how to respond when students are caught using a tobacco or nicotine product on school premises	O	O	O
B. Post signs marking a tobacco-free school zone, that is, a specified distance from school grounds where tobacco use is prohibited	O	O	O
C. Prohibit advertising and promotion for tobacco and nicotine products on school premises	O	O	O

Alcohol use prevention

8-15. Does this school have a policy prohibiting use of alcohol among the following groups on school premises? *(Mark yes or no or I do not know for each group.)*

	Yes	No	I do not know
A. Students	O	O	O
B. Teachers and other school staff	O	O	O
C. Visitors to the school	O	O	O

8-16. Does this school take the following actions to help reduce use of alcohol among students? *(Mark yes or no or I do not know for each action.)*

	Yes	No	I do not know
A. Implement policies on how to respond when students are caught using alcohol on school premises	O	O	O
B. Post signs marking an alcohol-free school zone, that is, a specified distance from school grounds where alcohol use is prohibited	O	O	O
C. Prohibit advertising and promotion for alcohol products on school premises	O	O	O

Guthold R, *et al. BMJ Open* 2023; 13:e071353. doi: 10.1136/bmjopen-2022-071353

Illicit drug use prevention

8-17. Does this school have a policy prohibiting use of illicit drugs among the following groups on school premises? *(Mark yes or no or I do not know for each group.)*

	Yes	No	I do not know
A. Students	O	O	O
B. Teachers and other school staff	O	O	O
C. Visitors to the school	O	O	O

8-18. Does this school take the following actions to help reduce use of illicit drugs among students? *(Mark yes or no or I do not know for each action.)*

	Yes	No	I do not know
A. Implement policies on how to respond when students are caught using illicit drugs on school premises	O	O	O
B. Post signs marking an illicit drug-free school zone, that is, a specified distance from school grounds where illicit drug use is prohibited	O	O	O

Crisis preparedness and emergency response

8-19. Does this school have a policy on crisis preparedness, response, and recovery from a natural disaster, conflict, pandemic, or other emergency situation?

A. Yes
B. No **(Go to 8-22)**
C. I do not know **(Go to 8-22)**

8-20. Does this policy ensure continuity for the following school services if the school building needs to be closed to students? *(Mark yes or no or I do not know for each service.)*

	Yes	No	I do not know
A. School health services	O	O	O
B. School food and nutrition services	O	O	O

8-21. Does this policy ensure continuity of instruction (possibly via remote learning) for the following subjects (or curricula) if the school building needs to be closed to students? *(Mark yes or no or I do not know for each subject.)*

	Yes	No	I do not know
A. Health education	O	O	O
B. Physical education	O	O	O
C. Other subjects	O	O	O

8-22. Does this school conduct regular emergency drills, such as fire or earthquake drills?

A. Yes
B. No
C. I do not know

8-23. Is this school officially designated to serve as a shelter for community members during or after a natural disaster?

A. Yes
B. No
C. I do not know

8-24. Does this school have measures in place in case of violence or conflict to help protect students and school staff (such as locked entry points, outsider identification processes, metal detectors, or security guards)?

A. Yes
B. No
C. I do not know

Eye health

8-25. Does this school have a policy requiring a minimum amount of outdoor activity daily to help prevent myopia or nearsightedness?

A. Yes
B. No
C. I do not know

Pregnant students

8-26. Does this school have a policy to allow pregnant students to attend school either in person or via remote learning?

A. Yes
B. No
C. I do not know

Note: Ask this question only if females attend this school.

Guthold R, *et al. BMJ Open* 2023; 13:e071353. doi: 10.1136/bmjopen-2022-071353

GLOBAL SCHOOL-BASED STUDENT HEALTH SURVEY

2021 Version

This survey is about your health and the things you do that may affect your health. Students like you all over your country are doing this survey. Students in many other countries around the world also are doing this survey. The information you give will be used to develop better health programs for young people like yourself.

DO NOT write your name on this survey or the answer sheet. The answers you give will be kept private. No one will know how you answer. Answer the questions based on what you really know or do. There are no right or wrong answers.

Completing the survey is voluntary. Your grade or mark in this class will not be affected whether or not you answer the questions. If you do not want to answer a question, just leave it blank.

Make sure to read every question. Fill in the circles on your answer sheet that match your answer. Use only the pencil you are given. When you are done, do what the person who is giving you the survey says to do.

Here is an example of how to fill in the circles:

Fill in the circles like this ● Not like this 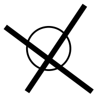 or 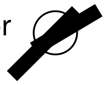

Survey

1. Do fish live in water?
   A. Yes
   B. No

Answer sheet

1. ● Ⓑ Ⓒ Ⓓ Ⓔ Ⓕ Ⓖ Ⓗ

Thank you very much for your help.

GSHS Core Questionnaire Respondent Demographics Module

1. How old are you?

    A. 11 years old or younger
    B. 12 years old
    C. 13 years old
    D. 14 years old
    E. 15 years old
    F. 16 years old
    G. 17 years old
    H. 18 years old or older

2. What is your sex?

    A. Male
    B. Female

3. In what grade/class/standard are you?
   COUNTRY SPECIFIC RESPONSE OPTIONS

    A. OPTION 1
    B. OPTION 2
    C. OPTION 3
    D. OPTION 4
    E. OPTION 5
    F. OPTION 6

Guthold R, *et al. BMJ Open* 2023; 13:e071353. doi: 10.1136/bmjopen-2022-071353

GSHS Core Questionnaire Alcohol Use Module

The next 8 questions ask about drinking alcohol. This includes drinking COUNTRY SPECIFIC EXAMPLES. Drinking alcohol does not include drinking a few sips of wine for religious purposes. A "drink" is a glass of wine, a bottle of beer, a small glass of liquor or home brew, or a mixed drink.

1. How old were you when you had your first drink of alcohol other than a few sips?

   A. I have never had a drink of alcohol other than a few sips
   B. 7 years old or younger
   C. 8 or 9 years old
   D. 10 or 11 years old
   E. 12 or 13 years old
   F. 14 or 15 years old
   G. 16 or 17 years old
   H. 18 years old or older

2. During the past 30 days, on how many days did you have at least one drink containing alcohol?

   A. 0 days
   B. 1 or 2 days
   C. 3 to 5 days
   D. 6 to 9 days
   E. 10 to 19 days
   F. 20 to 29 days
   G. All 30 days

3. During the past 30 days, on the days you drank alcohol, how many drinks did you **usually** drink per day?

   A. I did not drink alcohol during the past 30 days
   B. Less than one drink
   C. 1 drink
   D. 2 drinks
   E. 3 drinks
   F. 4 drinks
   G. 5 or more drinks

4. During the past 30 days, what is the largest number of alcoholic drinks you had in a row,that is, within a couple of hours?

   A. I did not drink alcohol during the past 30 days
   B. 1 or 2 drinks
   C. 3 drinks
   D. 4 drinks
   E. 5 drinks
   F. 6 or 7 drinks
   G. 8 or 9 drinks
   H. 10 or more drinks

5. During the past 30 days, how did you **usually** get the alcohol you drank? SELECT ONLY ONE RESPONSE.

   A. I did not drink alcohol during the past 30 days
   B. I bought it in a store, shop, or from a street vendor
   C. I gave someone else money to buy it for me
   D. I got it from my friends
   E. I got it from my family
   F. I stole it or got it without permission
   G. I got it some other way

6. During your life, how many times have you got into trouble at home, work, or school or got into fights, as a result of drinking alcohol?

   A. 0 times
   B. 1 or 2 times
   C. 3 to 5 times
   D. 6 to 9 times
   E. 10 to 19 times
   F. 20 or more times

Staggering when walking, not being able to speak right, throwing up, and passing out are some signs of being really drunk.

7. During your life, how many times have you drank so much alcohol that you were really drunk?

   A. 0 times
   B. 1 or 2 times
   C. 3 to 5 times
   D. 6 to 9 times
   E. 10 to 19 times
   F. 20 or more times

Guthold R, *et al. BMJ Open* 2023; 13:e071353. doi: 10.1136/bmjopen-2022-071353

GSHS Supplementary question on school attendance under the influence of alcohol

8. During the past 12 months, how many times did you attend school under the influence of alcohol?

   A. 0 times
   B. 1 or 2 times
   C. 3 to 5 times
   D. 6 to 9 times
   E. 10 to 19 times
   F. 20 to 39 times
   G. 40 or more times

GSHS Core Questionnaire Dietary Behaviours Module

The next 3 questions ask about your height, weight, and going hungry.

1. How tall are you without your shoes on? ON THE ANSWER SHEET, WRITE YOUR HEIGHT IN THE SHADED BOXES AT THE TOP OF THE GRID. THEN FILL IN THE CIRCLE BELOW EACH NUMBER.

Example

Height (cm)		
1	***5***	***3***
0	0	0
●	1	1
2	2	2
	3	●
	4	4
	●	5
	6	6
	7	7
	8	8
	9	9
	I do not know 9	

2. How much do you weigh without your shoes on? ON THE ANSWER SHEET, WRITE YOUR WEIGHT IN THE SHADED BOXES AT THE TOP OF THE GRID. THEN FILL IN THE CIRCLE BELOW EACH NUMBER.

Example

Weight (kg)		
0	***5***	***2***
●	0	0
1	1	1
2	2	●
	3	3
	4	4
	●	5
	6	6
	7	7
	8	8
	9	9
	I do not know 9	

3. During the past 30 days, how often did you go hungry because there was not enough food in your home?

A. Never
B. Rarely
C. Sometimes
D. Most of the time
E. Always

The next 4 questions ask about what you might eat and drink.

4. During the past 7 days, how many times did you eat fruit, such as COUNTRY SPECIFIC EXAMPLES?

A. I did not eat fruit during the past 7 days
B. 1 to 3 times during the past 7 days
C. 4 to 6 times during the past 7 days
D. 1 time per day
E. 2 times per day
F. 3 times per day
G. 4 or more times per day

5. During the past 7 days, how many times did you eat vegetables, such as COUNTRY SPECIFIC EXAMPLES?

A. I did not eat vegetables during the past 7 days
B. 1 to 3 times during the past 7 days
C. 4 to 6 times during the past 7 days
D. 1 time per day
E. 2 times per day
F. 3 times per day
G. 4 or more times per day

6. During the past 7 days, how many times did you drink a can, bottle, or glass of a carbonated soft drink, such as COUNTRY SPECIFIC EXAMPLES? (Do **not** count diet soft drinks.)

A. I did not drink carbonated soft drinks during the past 7 days
B. 1 to 3 times during the past 7 days
C. 4 to 6 times during the past 7 days
D. 1 time per day
E. 2 times per day
F. 3 times per day
G. 4 or more times per day

GSHS Core Questionnaire Dietary Behaviours Module

For this question, sugar-sweetened drinks include sports drinks (COUNTRY SPECIFIC EXAMPLES), energy drinks (COUNTRY SPECIFIC EXAMPLES), 100% fruit juices (COUNTRY SPECIFIC EXAMPLES), fruit drinks that are not 100% juice (COUNTRY SPECIFIC EXAMPLES), sugar-sweetened flavoured milks (COUNTRY SPECIFIC EXAMPLES), and sugar-sweetened teas, coffees, or flavoured waters.

For this question, DO NOT COUNT carbonated soft drinks measured in the previous question or diet or no calorie drinks.

7. During the past 7 days, how many times did you drink a can, bottle, or glass of a sugar-sweetened drink?

   A. I did not drink sugar-sweetened drinks during the past 7 days
   B. 1 to 3 times during the past 7 days
   C. 4 to 6 times during the past 7 days
   D. 1 time per day
   E. 2 times per day
   F. 3 times per day
   G. 4 or more times per day

GSHS Core Questionnaire Drug Use Module and supplementary question on school attendance under the influence of drugs

The next 4 questions ask about drug use. This includes using cannabis (also called marijuana), amphetamines, cocaine, inhalants, and heroin, and COUNTRY SPECIFIC EXAMPLES. Do not count tobacco and alcohol use.

1. How old were you when you first used drugs?

   A. I have never used drugs
   B. 7 years old or younger
   C. 8 or 9 years old
   D. 10 or 11 years old
   E. 12 or 13 years old
   F. 14 or 15 years old
   G. 16 or 17 years old
   H. 18 years old or older

2. During your life, how many times have you used cannabis (also called marijuana and COUNTRY SPECIFIC SLANG TERMS FOR CANNABIS)?

   A. 0 times
   B. 1 or 2 times
   C. 3 to 5 times
   D. 6 to 9 times
   E. 10 to 19 times
   F. 20 or more times

3. During the past 30 days, how many times did you use cannabis (also called marijuana and COUNTRY SPECIFIC SLANG TERMS FOR CANNABIS)?

   A. 0 times
   B. 1 or 2 times
   C. 3 to 5 times
   D. 6 to 9 times
   E. 10 to 19 times
   F. 20 or more times

4. During your life, how many times have you used amphetamines or methamphetamines (also called COUNTRY SPECIFIC SLANG TERMS FOR AMPHETAMINES AND METHAMPHETAMINES) for non-medical purposes?

   A. 0 times
   B. 1 or 2 times
   C. 3 to 5 times
   D. 6 to 9 times
   E. 10 to 19 times
   F. 20 or more times

5. During the past 12 months, how many times did you attend school under the influence of drugs, such as cannabis or cocaine?

   A. 0 times
   B. 1 or 2 times
   C. 3 to 5 times
   D. 6 to 9 times
   E. 10 to 19 times
   F. 20 or more times

Guthold R, *et al. BMJ Open* 2023; 13:e071353. doi: 10.1136/bmjopen-2022-071353

GSHS Core Questionnaire Hygiene Module

The next 3 questions ask about your oral health.

1. During the past 30 days, how many times per day did you **usually** clean or brush your teeth?

   A. I did not clean or brush my teeth during the past 30 days
   B. Less than 1 time per day
   C. 1 time per day
   D. 2 times per day
   E. 3 or more times per day

2. During the past 30 days, did you **usually** use a toothpaste that contains fluoride when you cleaned or brushed your teeth?

   A. I did not clean or brush my teeth during the past 30 days
   B. Yes, I usually used a toothpaste that contains fluoride
   C. No, I did not usually use a toothpaste that contains fluoride
   D. I do not know if the toothpaste I usually used contains fluoride

3. During the past 30 days, did a problem with your mouth, teeth, or gums cause you to miss classes or school?

   A. Yes
   B. No

The next 3 questions ask about washing your hands.

4. During the past 30 days, how often did you wash your hands before eating?

   A. Never
   B. Rarely
   C. Sometimes
   D. Most of the time
   E. Always

5. During the past 30 days, how often did you wash your hands after using the toilet or latrine?

   A. Never
   B. Rarely
   C. Sometimes
   D. Most of the time
   E. Always

6. During the past 30 days, how often did you use soap when washing your hands?

   A. Never
   B. Rarely
   C. Sometimes
   D. Most of the time
   E. Always

GSHS Core Questionnaire Mental Health Module

The next 3 questions ask about your friendships and feelings.

1. How many close friends do you have?

   A. 0 friends
   B. 1 friend
   C. 2 friends
   D. 3 or more friends

2. During the past 12 months, how often did you feel lonely?

   A. Never
   B. Rarely
   C. Sometimes
   D. Most of the time
   E. Always

3. During the past 12 months, how often were you so worried about something that you could not sleep at night?

   A. Never
   B. Rarely
   C. Sometimes
   D. Most of the time
   E. Always

Sometimes people feel so depressed about the future that they may consider attempting suicide, that is, taking some action to end their own life. The next 3 questions ask about attempted suicide.

4. During the past 12 months, did you **seriously** consider attempting suicide?

   A. Yes
   B. No

5. During the past 12 months, did you make a plan about how you would attempt suicide?

   A. Yes
   B. No

6. During the past 12 months, how many times did you attempt suicide?

   A. 0 times
   B. 1 time
   C. 2 or 3 times
   D. 4 or 5 times
   E. 6 or more times

GSHS Core Questionnaire Physical Activity Module

The next 4 questions ask about physical activity. Physical activity is any activity that increases your heart rate and makes you breathe hard. Physical activity can be done in sports, playing with friends, walking to school, or in physical education class. Some examples of physical activity are running, fast walking, biking, dancing, football, and COUNTRY SPECIFIC EXAMPLES.

1. During the past 7 days, on how many days were you physically active for a total of at least 60 minutes per day? ADD UP ALL THE TIME YOU SPENT IN ANY KIND OF PHYSICAL ACTIVITY EACH DAY.

    A. 0 days
    B. 1 day
    C. 2 days
    D. 3 days
    E. 4 days
    F. 5 days
    G. 6 days
    H. 7 days

2. During the past 7 days, on how many days did you do exercises to strengthen or tone your muscles, such as push-ups, sit-ups, or weightlifting?

    A. 0 days
    B. 1 day
    C. 2 days
    D. 3 days
    E. 4 days
    F. 5 days
    G. 6 days
    H. 7 days

3. During the past 7 days, on how many days did you walk or ride a bicycle to or from school?

    A. 0 days
    B. 1 day
    C. 2 days
    D. 3 days
    E. 4 days
    F. 5 days
    G. 6 days
    H. 7 days

4. During this school year, on how many days did you go to physical education (PE) class each week?

    A. 0 days
    B. 1 day
    C. 2 days
    D. 3 days
    E. 4 days
    F. 5 or more days

The next question asks about the time you spend mostly sitting or lying down when you are not in school or doing homework.

5. How much time do you spend during a **typical or usual** day sitting or lying down doing such things as watching television, playing computer games, talking with friends, using your mobile phone, traveling in a motor vehicle, napping, or doing other activities sitting or lying down, such as COUNTRY SPECIFIC EXAMPLES? (Do **not** count time spent sleeping at night.)

    A. Less than 1 hour per day
    B. 1 to 2 hours per day
    C. 3 to 4 hours per day
    D. 5 to 6 hours per day
    E. 7 to 8 hours per day
    F. More than 8 hours per day

The next question asks about how much sleep you get.

6. On an average school night, how many hours of sleep do you get?

    A. 4 or less hours
    B. 5 hours
    C. 6 hours
    D. 7 hours
    E. 8 hours
    F. 9 hours
    G. 10 hours
    H. 11 or more hours

Guthold R, *et al. BMJ Open* 2023; 13:e071353. doi: 10.1136/bmjopen-2022-071353

GSHS Core Questionnaire Protective Factors Module

The next 7 questions ask about your experiences at school and at home.

1. During the past 30 days, on how many days did you miss classes or school without permission?

   A. 0 days
   B. 1 or 2 days
   C. 3 to 5 days
   D. 6 to 9 days
   E. 10 or more days

2. During the past 30 days, how often were most of the students in your school kind and helpful?

   A. Never
   B. Rarely
   C. Sometimes
   D. Most of the time
   E. Always

3. During the past 30 days, how often were you able to talk to someone about difficult problems and worries?

   A. Never
   B. Rarely
   C. Sometimes
   D. Most of the time
   E. Always

4. During the past 30 days, how often did your parents or guardians understand your problems and worries?

   A. Never
   B. Rarely
   C. Sometimes
   D. Most of the time
   E. Always

5. During the past 30 days, how often did your parents or guardians check to see if your homework was done?

   A. Never
   B. Rarely
   C. Sometimes
   D. Most of the time
   E. Always

6. During the past 30 days, how often did your parents or guardians **really** know what you were doing with your free time?

   A. Never
   B. Rarely
   C. Sometimes
   D. Most of the time
   E. Always

7. During the past 30 days, how often did your parents or guardians go through your things without your approval?

   A. Never
   B. Rarely
   C. Sometimes
   D. Most of the time
   E. Always

Guthold R, *et al. BMJ Open* 2023; 13:e071353. doi: 10.1136/bmjopen-2022-071353

GSHS Core Questionnaire Sexual Behaviours That Contribute to HIV Infection, Other STI, and Unintended Pregnancy Module

The next 5 questions ask about sexual intercourse.

1. Have you ever had sexual intercourse?

   A. Yes
   B. No

2. How old were you when you had sexual intercourse for the first time?

   A. I have never had sexual intercourse
   B. 11 years old or younger
   C. 12 years old
   D. 13 years old
   E. 14 years old
   F. 15 years old
   G. 16 or 17 years old
   H. 18 years old or older

3. During your life, with how many people have you had sexual intercourse?

   A. I have never had sexual intercourse
   B. 1 person
   C. 2 people
   D. 3 people
   E. 4 people
   F. 5 people
   G. 6 or more people

4. The **last time** you had sexual intercourse, did you or your partner use a condom or [COUNTRY SPECIFIC SLANG TERM FOR CONDOM]?

   A. I have never had sexual intercourse
   B. Yes
   C. No

5. The **last time** you had sexual intercourse, what **one** method did you or your partner use to **prevent pregnancy**? SELECT ONLY ONE RESPONSE.

   A. I have never had sexual intercourse
   B. No method was used to prevent pregnancy
   C. Birth control pills
   D. Condoms
   E. An IUD or implant
   F. A shot, patch, or birth control ring
   G. Withdrawal or some other method including COUNTRY SPECIFIC METHOD
   H. I do not know

GSHS Core Questionnaire Tobacco Use Module

The next 3 questions ask about cigarette use. Cigarettes include manufactured cigarettes, roll-your-own cigarettes, and kretek cigarettes, and COUNTRY SPECIFIC EXAMPLES.

1. Have you ever tried or experimented with cigarette smoking, even one or two puffs?

   A. Yes
   B. No

2. How old were you when you first tried smoking a cigarette?

   A. I have never tried smoking a cigarette
   B. 7 years old or younger
   C. 8 or 9 years old
   D. 10 or 11 years old
   E. 12 or 13 years old
   F. 14 or 15 years old
   G. 16 or 17 years old
   H. 18 years old or older

3. During the past 30 days, on how many days did you smoke cigarettes?

   A. 0 days
   B. 1 or 2 days
   C 3 to 5 days
   D. 6 to 9 days
   E. 10 to 19 days
   F. 20 to 29 days
   G. All 30 days

The next question asks about other forms of smoked tobacco products other than cigarettes. This includes pipes, cigars, mini cigars, cigarillos, waterpipes, hookah, shisha, narghile, hubble-bubble, bidis, and heated tobacco products (HTPs) and COUNTRY SPECIFIC EXAMPLES.

4. During the past 30 days, on how many days did you use any form of smoked tobacco products other than cigarettes?

   A. 0 days
   B. 1 or 2 days
   C 3 to 5 days
   D. 6 to 9 days
   E. 10 to 19 days
   F. 20 to 29 days
   G. All 30 days

The next question asks about smokeless tobacco products. This includes snuff, chewing tobacco, dip, betel quid with tobacco, and gutka and COUNTRY SPECIFIC EXAMPLES.

5. During the past 30 days, on how many days did you use any form of smokeless tobacco products?

   A. 0 days
   B. 1 or 2 days
   C 3 to 5 days
   D. 6 to 9 days
   E. 10 to 19 days
   F. 20 to 29 days
   G. All 30 days

The next question asks about electronic cigarettes. Electronic cigarettes, or e-cigarettes, are electronic devices that usually contain a nicotine-based liquid that is vaporized and inhaled. You may also know them as vape-pens, hookah-pens, electronic hookahs (e-hookahs), electronic cigars (e-cigars), electronic pipes (e-pipes), or e-vaporizers. Some look like cigarettes and others look like pens or small pipes. They are battery-powered devices that produce vapor instead of smoke. They do not contain tobacco.

6. During the past 30 days, on how many days did you use electronic cigarettes?

   A. 0 days
   B. 1 or 2 days
   C 3 to 5 days
   D. 6 to 9 days
   E. 10 to 19 days
   F. 20 to 29 days
   G. All 30 days

GSHS Core Questionnaire Violence and Unintentional Injury Module

The next 3 questions ask about serious injuries that happened to you. An injury is serious when it makes you miss at least one full day of usual activities (such as school, sports, or a job) or requires treatment by a doctor or nurse.

1. During the past 12 months, how many **times** were you seriously injured?

   A. 0 times
   B. 1 time
   C. 2 or 3 times
   D. 4 or 5 times
   E. 6 or 7 times
   F. 8 or 9 times
   G. 10 or 11 times
   H. 12 or more times

2. During the past 12 months, what was the **most serious injury** that happened to you?

   A. I was not seriously injured during the past 12 months
   B. I had a broken bone, a dislocated joint, or a broken or knocked out tooth
   C. I had a cut or stab wound
   D. I had a concussion or other head or neck injury, was knocked out, or could not breathe
   E. I had a gunshot wound
   F. I had a bad burn
   G. I was poisoned or took too much of a drug
   H. Something else happened to me

3. During the past 12 months, **what was the major cause** of the most serious injury that happened to you?

   A. I was not seriously injured during the past 12 months
   B. I was in a motor vehicle accident or hit by a motor vehicle
   C. I fell
   D. Something fell on me or hit me
   E. I was attacked or abused or was fighting with someone
   F. I was in a fire or too near a flame or something hot
   G. I inhaled or swallowed something bad for me
   H. Something else caused my injury

The next question asks about physical attacks. A physical attack occurs when one or more people hit or strike someone, or when one or more people hurt another person with a weapon (such as a stick, knife, or gun). It is not a physical attack when two students of about the same strength or power choose to fight each other.

4. During the past 12 months, how many times were you physically attacked?

   A. 0 times
   B. 1 time
   C. 2 or 3 times
   D. 4 or 5 times
   E. 6 or 7 times
   F. 8 or 9 times
   G. 10 or 11 times
   H. 12 or more times

The next question asks about physical fights. A physical fight occurs when two students of about the same strength or power choose to fight each other.

5. During the past 12 months, how many times were you in a physical fight?

   A. 0 times
   B. 1 time
   C. 2 or 3 times
   D. 4 or 5 times
   E. 6 or 7 times
   F. 8 or 9 times
   G. 10 or 11 times
   H. 12 or more times

Guthold R, *et al. BMJ Open* 2023; 13:e071353. doi: 10.1136/bmjopen-2022-071353

GSHS Core Questionnaire Violence and Unintentional Injury Module

The next 3 questions ask about bullying. Bullying occurs when one or more <u>students or other people about your age</u> say or do hurtful or mean things. Bullying can occur when someone teases, threatens, ignores, spreads rumors about, calls someone a bad name, makes sexual remarks, or hits, shoves, or hurts another person <u>over and over again</u>. It is not bullying when two people of about the same strength or power argue or fight or tease each other in a friendly way.

6. During the past 12 months, were you bullied **on school property**?

   A. Yes
   B. No

7. During the past 12 months, were you bullied when you were **not on school property**?

   A. Yes
   B. No

Cyber bullying is a form of bullying using social media and other forms of online communication. Cyber bullying may happen on Instagram, Twitter, Snapchat, and Facebook, COUNTRY SPECIFIC EXAMPLES, and other social media platforms or through texting and email.

8. During the past 12 months, were you **cyber** bullied?

   A. Yes
   B. No

Guthold R, *et al. BMJ Open* 2023; 13:e071353. doi: 10.1136/bmjopen-2022-071353

GSHS Supplementary questions on Physical Activity and the COVID-19 pandemic

The next question asks about physical activity. It is similar to but different than a question you were asked earlier. As a reminder, physical activity is any activity that increases your heart rate and makes you breathe hard. Physical activity can be done in sports, playing with friends, walking to school, or in physical education class. Some examples of physical activity are running, fast walking, biking, dancing, football, badminton, tennis, kabaddi, kho-kho, cricket, and swimming.

1. During the past 7 days, did you do at least **an average of** 60 minutes per day of physical activity across the 7 days?

   A. Yes
   B. No

Beginning in late 2019, the world experienced the coronavirus disease (COVID-19) pandemic. As part of the response to this pandemic, some schools and businesses were closed and some people were required to stay at home. Your community might still be experiencing the pandemic. The next question ask about the COVID-19 pandemic.

1. During the COVID-19 pandemic, did you attend school from home at least some of the time using a computer, mobile phone, or other electronic device?

   A. Yes
   B. No

Guthold R, *et al. BMJ Open* 2023; 13:e071353. doi: 10.1136/bmjopen-2022-071353

Global School-Based Student Health Survey (GSHS) Parental/Guardian Notification Form

[Date]

**Introduction**	[Name of school] is participating in a study to help empower students to lead change using health data. Three other cities around the world are also participating. The study is sponsored by [name of agency]. The study has two main components.
**Global School-Based Student Health Survey (GSHS)**	The GSHS is a paper-and-pencil survey being given to a small number of students aged 13-17 across our city. It asks students about health behaviours and experiences. Students will not get any immediate benefit from taking part in the GSHS. However, the results of this survey will be used by some students, teachers, and local leaders to help develop a program to reduce student risk behaviors. Questions will be asked about the following topics: [list the selected core modules].
**Timeframe**	Students will be asked to fill out a paper questionnaire during regular class time and it will take about 30 to 45 minutes to complete.
**Physical activity study**	In addition to the GSHS, students from one class in each school will be asked to wear an activity monitor on their wrist for one week. The activity monitor will measure physical activity (sitting, standing, walking, running, and lying down). The activity monitor will not interfere with their daily activities. Students in activity monitor classrooms will be given detailed directions about how to wear the monitor. At the end of the week, they will be asked to complete the GSHS just like students in all other sampled classes.
**Student privacy**	Survey procedures have been designed to protect student privacy. Students do not put their name on the questionnaire or answer sheet. No school or student is ever mentioned by name in a report of the results. Some students may find some questions to be a little sensitive.
**Voluntary participation**	We would like all students to take part in the survey and physical activity study, **but both activities are voluntary**. No action will be taken against the school, you, or a student, if a student does not take part. Students can skip any question that they do not wish to answer, students may stop participating in the survey at any point without penalty, and students may refuse to wear the activity monitor.

**Questions**	If you have any questions, please contact [name and contact information of survey coordinator].<br>Thank you for your cooperation.

Global School-Based Student Health Survey (GSHS) Parental/Guardian Permission Form

**Introduction**	[Name of school] is participating in a study to help empower students to lead change using health data. Three other cities around the world are also participating. The study is sponsored by [name of agency]. The study has two main components.
**Global School-Based Student Health Survey (GSHS)**	The GSHS is a paper-and-pencil survey being given to a small number of students aged 13-17 across our city. It asks students about health behaviours and experiences. Students will not get any immediate benefit from taking part in the GSHS. However, the results of this survey will be used by some students, teachers, and local leaders to help develop a program to reduce student risk behaviors. Questions will be asked about the following topics: [list the selected core modules].
**Timeframe**	Students will be asked to fill out a paper questionnaire during regular class time and it will take about 30 to 45 minutes to complete.
**Physical activity study**	In addition to the GSHS, students from one class in each school will be asked to wear an activity monitor on their wrist for one week. The activity monitor will measure physical activity (sitting, standing, walking, running, and lying down). The activity monitor will not interfere with their daily activities. Students in activity monitor classrooms will be given detailed directions about how to wear the monitor. At the end of the week, they will be asked to complete the GSHS just like students in all other sampled classes.
**Student privacy**	Survey procedures have been designed to protect student privacy. Students do not put their name on the questionnaire or answer sheet. No school or student is ever mentioned by name in a report of the results. Some students may find some questions to be a little sensitive.
**Voluntary participation**	We would like all students to take part in the survey and physical activity study, **but both activities are voluntary**. No action will be taken against the school, you, or a student, if a student does not take part. Students can skip any question that they do not wish to answer, students may stop participating in the survey at any point without penalty, and students may refuse to wear the activity monitor.
**Questions**	If you have any questions, please contact [name and contact information of survey coordinator]. Thank you for your cooperation.

Please complete and return the following permission form by [date] if you do NOT want your student to take part in the study.

Student's name:____

I have read this form and know what the survey is about.
[ ] My student may **NOT** take part in this survey.

Parent/guardian signature:_______________________________________ Date:___________________

Student Assent Form

Introduction

Your school is part of a study to help empower students to lead change using health data. This study is being done in three other cities around the world.

The study has several parts:

- a survey (called the Global School-based Student Health Survey – GSHS) where you will be asked to answer questions about your health;
- a physical activity study where some students will be asked to wear an activity monitor on their wrist;
- a workshop where some students will be asked to help come up with ideas to improve the health of all students in your school.

Global School-Based Student Health Survey (GSHS)

This survey is about your health and the things you do that may affect your health. Students like you from __ schools all over your city are doing this survey. Students in many other countries around the world have also done this survey before. The information you give will be used to develop better health programs for young people like yourself.

You will be asked to complete a paper questionnaire during regular class time. It will take about 30 to 45 minutes.

The answers you give will be kept private. No one will know how you answer. Please answer the questions based on what you really know or do. There are no right or wrong answers.

Physical activity study

Students from some classes also will be asked to wear an activity monitor on their wrist for one week. The activity monitor will measure physical activity (sitting, standing, walking, running, and lying down). The activity monitor will not interfere with anything you do. You will be given detailed directions about how to wear the monitor. At the end of the week, you will give the activity monitor back and then complete the GSHS just like students in other classes. The information collected with the activity monitor will be kept private and your name will not be recorded in the activity monitor.

Workshop

Some students will be asked to take part in data-to-action workshops. In these workshops, students, teachers, and local leaders will look at the study data and develop ideas on how the health of all students in your school could be improved.

Your privacy

The entire study has been designed to protect your privacy. There will not be any student, class, or school names on any questionnaire or answer sheet or recorded in the activity monitor. No school or student will be mentioned by name in a report of the results.

**Voluntary participation**	We would like all selected students to take part in the study, **but your participation is voluntary**. Your grade or mark in this class will not be affected whether or not you answer the questions or wear the activity monitor. If you do not want to answer a question, just leave it blank, you may stop participating in the study at any point without penalty, you may refuse to wear the activity monitor, and you do not have to volunteer for the data-to-action workshops.
**Questions**	If you have any questions, please contact [name and contact information of survey coordinator]. Thank you.

Please complete and return the following form by [date] if you do NOT want to take part in the study.

Your name:____

I have read this form and know what the study is about.
[ ] I do **NOT** want to take part in this study.

Student signature:_______________________________________ Date:___________________

Data to action workshop to improve adolescent health Parental/Guardian Notification Form

**Introduction**	[Name of school] has recently done a Global School-based Student Health Survey (GSHS) and a Global School Health Policies and Practices Survey (G-SHPSS) sponsored by [name of agency]. The GSHS collects information on health behaviours in 13-17 year old students, and the G-SHPPS on existing practices in schools. In the data to action workshop, the collected information will be used to plan interventions to improve the health of adolescents.
**Global AA-HA! workshop**	The data to action workshop will follow the Global Accelerated Action for the Health of Adolescents (Global AA-HA!) approach to improve young people's health. During the workshop, students, teachers and local authorities will explore information on student's behaviours and school practices together, identify where action is most needed, and set priorities for interventions to improve adolescent health. Students will not get any immediate benefit from taking part in the workshop. However, the results of the workshop will help students and other adolescents in the future.
**Timeframe**	Participation in the workshop will take about two hours per day on three consecutive days.
**Student privacy**	Workshop procedures have been designed to protect student privacy. No school or student will ever be mentioned by name in workshop documents.
**Voluntary participation**	**Participation in the workshop is voluntary**. Only parents/guardians of students that have put their name forward for voluntary participation in the workshop receive this permission form. If students wish, they may stop participating in the workshop at any point without penalty.
**Questions**	If you have any questions, please contact [name of local contact]. Thank you for your cooperation.

Data to action workshop to improve adolescent health Parental/Guardian Permission Form

**Introduction**	[Name of school] has recently done a Global School-based Student Health Survey (GSHS) and a Global School Health Policies and Practices Survey (G-SHPSS) sponsored by [name of agency]. The GSHS collects information on health behaviours in 13-17 year old students, and the G-SHPPS on existing practices in schools. In the data to action workshop, the collected information will be used to plan interventions to improve the health of adolescents.
**Global AA-HA! workshop**	The data to action workshop will follow the Global Accelerated Action for the Health of Adolescents (Global AA-HA!) approach to improve young people's health. During the workshop, students, teachers and local authorities will explore information on student's behaviours and school practices together, identify where action is most needed, and set priorities for interventions to improve adolescent health. Students will not get any immediate benefit from taking part in the workshop. However, the results of the workshop will help students and other adolescents in the future.
**Timeframe**	Participation in the workshop will take about two hours per day on three consecutive days.
**Student privacy**	Workshop procedures have been designed to protect student privacy. No school or student will ever be mentioned by name in workshop documents.
**Voluntary participation**	**Participation in the workshop is voluntary**. Only parents/guardians of students that have put their name forward for voluntary participation in the workshop receive this permission form. If students wish, they may stop participating in the workshop at any point without penalty.
**Questions**	If you have any questions, please contact [name of local contact]. Thank you for your cooperation.

Please complete and return the following permission form by [date] if you do NOT want your student to take part in the workshop.

Student's name:____

I have read this form and know what the workshop is about.

[ ] My student may **NOT** take part in this workshop.

Parent/guardian signature:____ Date:____________________

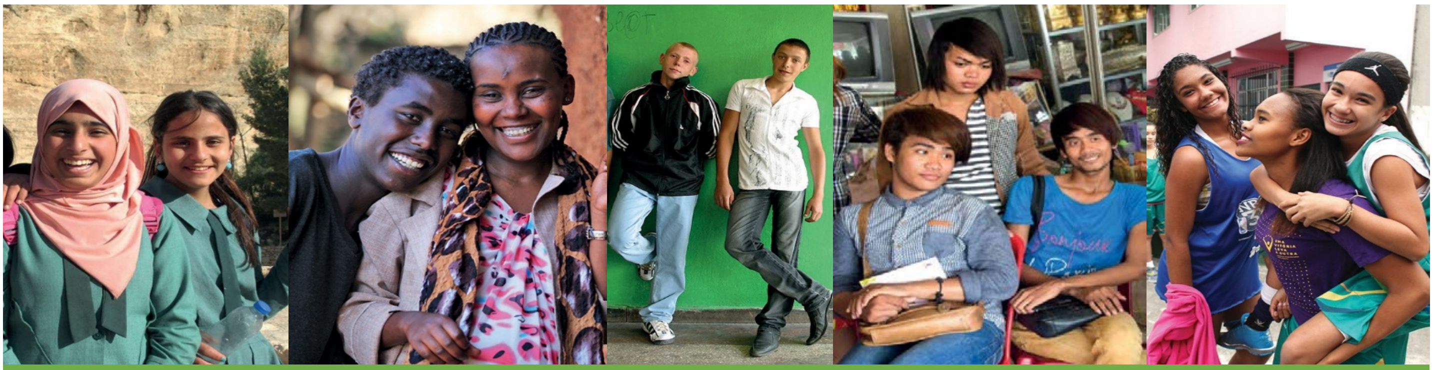

EMPOWERING ADOLESCENTS TO LEAD CHANGE USING HEALTH DATA

DATA-TO-ACTION WORKSHOP

Concept note

Background and Scope

Comprehensive local data on adolescent health are often lacking, particularly in lower-resource settings. Furthermore, there are knowledge gaps as to which interventions are effective to support healthy behaviours. To fill these gaps and generate new data and information, WHO across all three levels is conducting a study entitled "Empowering adolescents to lead change using health data", funded by Fondation Botnar.

The overarching aim of the study is to generate health information for students from cities in four low- and middle-income countries on which to plan, implement and subsequently evaluate a package of interventions to improve health outcomes. The study uses a cluster-randomized controlled trial design and is conducted in four cities of different world regions, namely Fez, Morocco; Jaipur, India; Saint Catherine Parish, Jamaica; and Sekondi-Takoradi, Ghana.

The study consists of several components: First, baseline assessments are conducted in randomly-selected schools of the four cities, using existing WHO tools: the Global School-based Student Health Survey (GSHS) to assess health behaviours and protective factors among students, and the Global School Health Policies and Practices Survey (G-SHPPS) to assess school policies. These data are being enhanced by photovoice (photos and drawings from the students). Second, in half of the schools (intervention arm), the GSHS and G-SHPPS baseline results will be used to plan and subsequently implement a package of interventions. This will be done following the approach of the Global Accelerated Action for the Health of Adolescents (AA-HA!) and based on the framework of the Global Standards for Health Promoting Schools. Third, follow-up surveys will be conducted two years after baseline to assess the differential change in health outcomes between the intervention and the control group.

The baseline surveys in the respective cities have been conducted from August 2022 – May 2023. The results from these baseline surveys will be used in a data-to-action workshop in each city, taking place a few months after completion of data collection.

Guthold R, et al. BMJ Open 2023; 13:e071353. doi: 10.1136/bmjopen-2022-071353

Workshop objectives

The objectives of the data-to-action workshop are:

- Familiarize workshop participants with the study and the data;
- Identify adolescent health needs based on the collected data (needs assessment);
- Identify gaps in policies/programmes/practices (landscape analysis);
- Identify priority areas for action based on the needs assessment and landscape analysis;
- Develop a package of interventions to be implemented based on the identified priority areas for action;
- Develop an action plan with timelines and a plan to monitor implementation of the interventions.

Expected outcomes

Expected outcomes of the data-to-action workshop are:

- Workshop participants familiarized with the study and the data;
- Adolescent health needs identified;
- Gaps in policies/programmes/practices identified;
- Priority areas for action identified;
- Package of interventions to be implemented based on the identified priority areas for action identified;
- Action plan with timelines and a plan to monitor implementation of the interventions developed.

Methodology

The data-to-action workshop will be held over three days. The workshop will be held in the respective language spoken in each city and include plenary and group work sessions.

Participants

The data-to-action workshop will bring together:

- Education and health authority representatives;
- Community leaders;
- From the selected intervention schools in each city:
    - School focal points (including teachers, school administrators and school health persons);
    - Students;
    - Parents;
- Representatives of WHO and UNESCO;
- External partners.

Guthold R, *et al. BMJ Open* 2023; 13:e071353. doi: 10.1136/bmjopen-2022-071353

Agenda

DAY 1

**DAY 1**		
Goal: Familiarize workshop participants with the study and the data and identify health needs		
**9.00 – 9.15**	Registration	
**9.15 - 9.30**	Welcome and opening remarks	Plenary
**9.30 – 10.00**	Workshop background and objectives<br>- Ice-breaking activity<br>- Participant's expected goals<br>- Expected outcomes of the workshop	Plenary
**10.00 – 10.30**	Introductory presentations<br>- Overview of the study and methods<br>- Overview of study implementation in the respective city	Plenary
**10.30 – 10.45**	Break	
**10.45 – 11.45**	Identifying health needs based on the GSHS results<br>- Presentation and review of GSHS results<br>- Group work	Plenary, Group work
**11.45 – 12.30**	Identifying health needs based on GSHS results<br>- Report back from group work	Group work
**12.30 – 13.30**	Lunch	
**13.30 – 15.00**	Identifying health needs based on photovoice<br>- Presentation and review of photovoice photos and drawing<br>- Group work and report back from group work	Plenary, Group work
**15.00 – 15.15**	Break	
**15.15 – 16.30**	Agreeing on health needs	Plenary

DAY 2

**DAY 2**		
Goal: Identify gaps in policies/programmes/practices and priority areas for action, draft interventions		
**9.00 – 9.20**	Recap of Day 1<br>- Review of expectations and of findings of Day 1	Plenary
**9.20 – 9.50**	Existing national & regional adolescent health programmes<br>- Presentations and short Q & A	Plenary
**9.50 – 10.30**	Landscape analysis: what policies/practices are in place and where are gaps? (Focus on health issues identified in Day 1)<br>- Presentation of G-SHPPS results<br>- Review of G-SHPPS results (group work)	Plenary, Group work
**10.30 – 10.45**	Break	
**10.45 – 11.30**	Identification of gaps in policies/programmes/practices	Plenary

Guthold R, *et al. BMJ Open* 2023; 13:e071353. doi: 10.1136/bmjopen-2022-071353

	- Report back from group work	
**11.30 – 12.30**	Identification of priority areas for action	Plenary
**12.30 – 13.30**	Lunch	
**13.30 – 14.30**	Intervention planning:<br>- Review evidence-based interventions<br>- Identification of possible intervention ideas	Group work
**14.15 – 15.00**	Intervention planning:<br>- Report back from group work	Plenary
**15.00 – 15.15**	Break	
**15.15 – 16.30**	Revisiting and prioritizing possible interventions	Plenary

DAY 3

**DAY 3**		
Goal: Develop a package of interventions and an action plan with timelines and monitoring		
**9.00 – 9.20**	Recap of Day 2<br>- Review of expectations and of findings of Day 1	Plenary
**9.20 – 10.30**	Finalizing a package of prioritized interventions	Plenary
**10.30 – 10.45**	Break	
**10.45 – 12.30**	Developing a logic model:<br>- Presentation of a logic model<br>- Transferring results of Day 2 in a logic model	Plenary, Group work
**12.30 – 13.30**	Lunch	
**13.30 – 15.00**	Developing a unified action plan<br>- What can be measured and how?<br>- Roles and responsibilities	Group work, Plenary
**15.00 – 15.15**	Break	
**15.15 – 16.45**	Developing a unified action plan, cont.<br>- Timelines for implementation	Plenary
**16.45 – 17.00**	Feed-back, congratulations and closing remarks	Plenary

Guthold R, *et al. BMJ Open* 2023; 13:e071353. doi: 10.1136/bmjopen-2022-071353
