# Supplementary material for: Effectiveness of a participatory approach to develop school health interventions in four low resource cities: study protocol of the ‘empowering adolescents to lead change using health data’ cluster randomised controlled trial
Source: BMJ Open. 2023 Jul 5;13(7):e071353. doi: 10.1136/bmjopen-2022-071353 (PMC10335517; doi:10.1136/bmjopen-2022-071353)
Supplement: Author's manuscript [file bmjopen-2022-071353.draft_revisions.pdf]

BMJ Open is committed to open peer review. As part of this commitment we make the peer review history of every article we publish publicly available.

When an article is published we post the peer reviewers' comments and the authors' responses online. We also post the versions of the paper that were used during peer review. These are the versions that the peer review comments apply to.

The versions of the paper that follow are the versions that were submitted during the peer review process. They are not the versions of record or the final published versions. They should not be cited or distributed as the published version of this manuscript.

BMJ Open is an open access journal and the full, final, typeset and author-corrected version of record of the manuscript is available on our site with no access controls, subscription charges or pay-per-view fees (http://bmjopen.bmj.com).

If you have any questions on BMJ Open's open peer review process please email info.bmjopen@bmj.com

BMJ Open

Empowering adolescents to lead change using health data: Study protocol of a cluster randomized controlled trial at schools in four cities from different world regions

Journal:	*BMJ Open*
Manuscript ID	bmjopen-2022-071353
Article Type:	Protocol
Date Submitted by the Author:	22-Dec-2022
Complete List of Authors:	Guthold, Regina ; WHO, Maternal, Newborn, Child and Adolescent Health and Ageing Department<br>Kann, Laura; WHO, Department of Noncommunicable Diseases<br>Bhatti, Lubna; WHO, Department of Noncommunicable Diseases<br>Abduvahobov, Parviz; UNESCO, Section of Health and Education, Education Sector<br>Ansong, Joana; WHO Country Office for Ghana<br>Atkinson, Uki; National Council on Drug Abuse<br>Baltag , Valentina; World Health Organisation, Maternal, Newborn, Child and Adolescent Health and Ageing Department<br>Caffe, Sonja; PAHO, Family, Health Promotion and Life Course<br>Caixeta, Roberta ; Pan American Health Organization, Noncommunicable Diseases and Mental Health Department<br>Diallo, Cheick Bady; WHO Regional Office for Africa, Universal Health Coverage/Communicable and Noncommunicable Diseases<br>Fouad, Heba; WHO Regional Office for the Eastern Mediterranean, Noncommunicable Diseases and Mental Health Department<br>Haddad, Sally; University of Bern, Department of Health Science, Institute of Sport Science<br>Hachri, Hafid; WHO Country Office for Morocco<br>Jaggi, Jeannine; University of Bern, Department of Health Science, Institute of Sport Science<br>Joshi, Pradeep; WHO Country Office for India<br>Karna, Priya; WHO Country Office for India<br>Louazani, Sidi Allal; WHO, Department of Noncommunicable Diseases<br>Mbola Mbassi, Symplice; WHO Regional Office for Africa, Universal Health Coverage/Life Course<br>Mehta, Rajesh; WHO Regional Office for South-East Asia<br>Mudgal, Yogendra; Department of School Education<br>Nigg, Claudio; University of Bern, Department of Health Science, Institute of Sport Science<br>Okely, Tony; University of Wollongong, School of Education<br>Ondarsuhu, Dolores; Pan American Health Organization, Noncommunicable Diseases and Mental Health Department<br>Ouaourir, Tahar; Ministry of Health, Population Department<br>Trhari, Fatima Zahra; Ministry of Health, Population Department<br>Riley, Leanne; World Health Organization, Department of Noncommunicable Diseases

Keywords:	EPIDEMIOLOGIC STUDIES, PAEDIATRICS, Community child health < PAEDIATRICS

SCHOLARONE™ Manuscripts

TITLE PAGE

Empowering adolescents to lead change using health data: Study protocol of a cluster randomized controlled trial at schools in four cities from different world regions

Author names

Regina Guthold,[1] Laura Kann,[2] Lubna Bhatti,[2] Parviz Abduvahobov,[3] Joana Ansong,[4] Uki Atkinson,[5] Valentina Baltag,[1] Sonja Caffe,[6] Roberta de Betânia Caixeta,[7] Cheick Bady Diallo,[8] Heba Fouad,[9] Sally Haddad,[10] Hafid Hachri,[11] Jeannine Jaggi,[10] Pradeep Joshi,[12] Priya Karna,[12] Sidi Allal Louazani,[2] Symplice Mbola Mbassi,[13] Rajesh Mehta,[14] Yogendra Mudgal,[15] Claudio R. Nigg,[10] Anthony D. Okely,[16] Dolores Ondarsuhu,[7] Tahar Ouaourir,[17] Fatima Zahra Trhari,[17] Leanne M. Riley,[2]

Author affiliations

[1] Maternal, Newborn, Child and Adolescent Health and Ageing Department, WHO, Geneva, Switzerland

[2] Department of Noncommunicable Diseases, WHO, Geneva, Switzerland

[3] Section of Health and Education, Education Sector, UNESCO, Paris, France

[4] WHO Country Office for Ghana, Accra, Ghana

[5] National Council on Drug Abuse, Kingston, Jamaica

[6] Family, Health Promotion and Life Course, Pan American Health Organization, Washington D.C., USA

For peer review only - http://bmjopen.bmj.com/site/about/guidelines.xhtml

[7] Noncommunicable Diseases and Mental Health Department, Pan American Health Organization, Washington D.C., USA

[8] Universal Health Coverage/Communicable and Noncommunicable Diseases, WHO Regional Office for Africa, Brazzaville, Congo

[9] Noncommunicable Diseases and Mental Health Department, WHO Regional Office for the Eastern Mediterranean, Cairo, Egypt

[10] Department of Health Science, Institute of Sport Science, University of Bern, Bern, Switzerland

[11] WHO Country Office for Morocco, Rabat, Morocco

[12] WHO Country Office for India, New Delhi, India

[13] Universal Health Coverage/Life Course, WHO Regional Office for Africa, Brazzaville, Congo

[14] WHO Regional Office for South-East Asia, New Delhi, India

[15] Department of School Education, Jaipur, India

[16] School of Health and Society and Early Start, University of Wollongong, Australia and Illawarra Health and Medical Research Institute, Wollongong, Australia

[17] Population Department, Ministry of Health, Rabat, Morocco

Corresponding author

Address correspondence to: Regina Guthold, Ph.D., Maternal, Newborn, Child and Adolescent Health and Ageing Department, WHO, 20, Avenue Appia, CH-1211 Geneva, Switzerland.

E-mail address: gutholdr@who.int

For peer review only

For peer review only - http://bmjopen.bmj.com/site/about/guidelines.xhtml

Word count

Abstract: 296

Main text: 3,910

Number of tables, figures, and references

Tables: 2

Figures: 1

References: 43

Keywords

Adolescent health, Global health, Physical activity, Randomized controlled trial, Schools, Health Promotion

For peer review only

For peer review only - http://bmjopen.bmj.com/site/about/guidelines.xhtml

ABSTRACT

Introduction

Comprehensive local data on adolescent health are often lacking, particularly in lower resource settings. Furthermore, there are knowledge gaps around which interventions are effective to support healthy behaviours. This study generates health information for students from cities in four low- and middle-income countries to plan, implement and subsequently evaluate a package of interventions to improve health outcomes.

Methods and analysis

We will conduct a cluster randomized controlled trial in schools in Fez, Morocco; Jaipur, India; Saint Catherine Parish, Jamaica; and Sekondi-Takoradi, Ghana. In each city, approximately 30 schools will be randomly selected and assigned to the control or intervention arm. Baseline data collection includes three components. First, a Global School Health Policies and Practices Survey (G-SHPPS) to be completed by principals of all selected schools. Second, a Global School-based Student Health Survey (GSHS) to be administered to a target sample of n=3,153 13-17-year-old students of randomly selected classes of these schools, including questions on alcohol, tobacco and drug use, diet, hygiene, mental health, physical activity, protective factors, sexual behaviors, violence and injury. Third, a study validating the GSHS physical activity questions against wrist-worn accelerometry in one randomly selected class in each control school (n approximately 300 students per city). Intervention schools will develop a suite of interventions using a participatory approach driven by students and involving parents/guardians, teachers and community stakeholders. Interventions will aim to change existing structures and policies

For peer review only - http://bmjopen.bmj.com/site/about/guidelines.xhtml

at schools to positively influence students' behavior, using collected data and guided by the framework for *Making Every School a Health Promoting School*. Outcomes will be assessed for differential change after a two-year follow-up.

Ethics and dissemination

The study was approved by ethics review boards of each site, and by WHO's Research Ethics Review Committee. Findings will be shared through open access publications and conferences.

Trial registration number

NCT04963426

For peer review only

For peer review only - http://bmjopen.bmj.com/site/about/guidelines.xhtml

STRENGTHS AND LIMITATIONS OF THIS STUDY

- This is the first known-study to generate and directly use adolescent health information to plan, implement and subsequently evaluate a package of interventions to improve health outcomes across four cities from different world regions, with a focus on underserved locations.
- The approach to develop interventions is data-informed, systematic and evidence-based, culturally adapted and flexible, and participatory, driven by adolescents, parents, and school- and community stakeholders.
- This study uses existing global tools for data collection, intervention planning and implementation. If effective, it can easily be replicated by other cities worldwide and scaled-up.
- The COVID-19 pandemic and related school closures have led to delays in study implementation and may impact outcomes.
- Internet connectivity may be poor in some study sites, and alternative methods for study components, such as electronic collection of school policy information, may need to be explored, potentially leading to delays in study implementation.

For peer review only - http://bmjopen.bmj.com/site/about/guidelines.xhtml

INTRODUCTION

Adolescence is a unique period in life with rapid physical, cognitive and psychosocial growth. It is an important time for laying foundations of future health, including, for example, establishing behaviors related to noncommunicable diseases (NCDs) such as tobacco and alcohol use, diet and physical activity, as well as behaviors linked to sexual or mental health (1). Implementing effective, evidence-based interventions could substantially reduce risk behaviors and support protective factors among adolescents and improve their future adult health (2, 3). However, several critical barriers exist to implementing such interventions for adolescents.

First, there is a lack of relevant and context-specific adolescent health information, particularly in low- and middle-income countries (LMICs), that is essential to inform intervention planning (4). Where data exist, they are primarily national-level estimates on specific health conditions that often mask variations and unequal progress by smaller geographical units (5, 6). Local data are needed to uncover and address these inequalities and develop the most relevant, effective, age-appropriate and context-specific interventions for adolescents. This includes data on policies and practices affecting adolescent's behavior, and on their health behaviors and protective factors. Collecting this information is of particular importance in usually neglected urban settings where little data exist, such as cities other than capitals (called 'secondary cities' hereafter).

Second, information on adolescent health is sometimes collected with questionnaires that may not have been validated for the populations in which they are used. Physical activity is one example where the most internationally used questions for adolescents have only been tested for validity in a few high-income countries (7, 8). To produce useful data to inform decision

For peer review only - http://bmjopen.bmj.com/site/about/guidelines.xhtml

making, it is essential for these questions to be tested more widely. Another reason to test adolescent physical activity questions is related to the change in recommended levels of physical activity for this age group. Most currently used questions are based on the 2010 WHO guidelines on physical activity that recommended adolescents to be physically active for at least 60 minutes *each* day (9). In 2020, these guidelines were updated and now state that adolescents should engage in at least *an average* of 60 minutes of activity per day throughout the week (10). Consequently, there is a need for adolescent physical activity questions to be tested against the updated guidelines.

Third, there are significant knowledge gaps about which interventions might be effective in targeting risk behaviors and supporting protective factors in adolescents (11). Available evidence is strongest for universal school-based interventions targeting multiple risk behaviors, however, most of the included studies were conducted in high-income countries, while evidence from LMICs is lacking (11).

Fourth, adolescents are too rarely involved in health promotion planning, decision-making and implementation. Yet, participatory approaches engaging adolescents are crucial for sustainable change in cognitions and behavior (12, 13) and thus warranted.

Global tools that help overcome the aforementioned barriers exist. The Global School Health Policies and Practices Survey (G-SHPPS) generates important school-level policy information (14). The Global School-based Student Health Survey (GSHS) collects data on students' health behaviors and protective factors (15). The Global Accelerated Action for the Health of Adolescents (Global AA-HA!) provides a systematic, participatory approach to understanding adolescent health needs based on data, prioritizing these needs in the local context and

For peer review only

For peer review only - http://bmjopen.bmj.com/site/about/guidelines.xhtml

planning, implementing, monitoring and evaluating appropriate interventions (16). The initiative *Making Every School a Health Promoting School* provides an evidence-based framework for the implementation of eight Global Standards to improve students' health (17) and promotes a whole-school approach that has demonstrated positive effects on health (18, 19, 20, 21). Both the Global AA-HA! and *Making Every School a Health Promoting School* emphasize the importance of students involvement in health promotion planning, decision-making and implementation in a manner that is empowering. Finally, photovoice, facilitates adolescent engagement by allowing them to pictorially take impressions of health facilitators and barriers in their schools and community to inform intervention planning (22, 23, 24). These available global tools will be used to achieve the goals of this study. The overarching aim is to generate health information for students of secondary cities from four LMICs of different world regions that will be directly used to plan, implement and evaluate a package of interventions for programme planning, policy, and structural change to improve students' health.

Specific objectives include: First, to assess health policies and practices currently in place in selected schools of the four cities. Second, to understand the levels of health risk and protective factors among school-going adolescents in these cities. Third, to validate currently used questions to assess students' physical activity behavior with wearable accelerometers. Fourth, to plan and evaluate a participatory intervention approach that focuses on changing existing structures, policies, and practices and on implementing programmes in and around schools to improve health outcomes for students.

For peer review only

METHODS AND ANALYSIS

Study design and locations

We will conduct a multi-site two-armed cluster randomized controlled trial using a nested cross-sectional design. The clusters are defined as schools and classes within schools. The study locations are spread across four world regions and include the cities of Fez, Morocco; Jaipur, India; Saint Catherine Parish, Jamaica; and Sekondi-Takoradi, Ghana.

In each city, approximately 30 schools will be selected and randomly assigned to either the intervention or control group. At baseline, all sampled schools will conduct a G-SHPPS to assess school policies and practices (14) and a GSHS to assess risk behaviors and protective factors among students (15). Additionally, in the control group of each city, we will conduct studies to validate GSHS physical activity questions against an accelerometer (Axivity AX3 (25)) to be worn by students on the wrist for eight days prior to the baseline survey.

Following the baseline surveys, the intervention schools will participate in data-to-action workshops to develop a package of interventions. Students, parents, teachers, local authorities and researchers will work together to find and prioritize policy and programme solutions to establish a unified action plan. Subsequently, these interventions will be implemented and study processes and outcomes will be assessed for differential change via repeat G-SHPPS and GSHS surveys after a two-year follow-up. We hypothesize that the prevalence of behavioral risk factors would be lower and the prevalence of protective factors higher among students in schools in the intervention group compared to the control group at follow-up. An overview of our study design is shown in Figure 1.

For peer review only - http://bmjopen.bmj.com/site/about/guidelines.xhtml

Study population and sampling

This study's target population is 13- to 17-year-old students. In each city, following standard G-SHPPS and GSHS sampling procedures (14, 15), approximately 30 schools will be randomly selected from a list of schools including every school in the city with eligible classes (classes that the target population usually attends). Selection will be done with probability proportional to the number of students in the eligible schools.

For the GSHS, in a second sampling stage, classes will be randomly selected from all eligible classes. All students of selected classes will be asked to participate in the survey.

The city sample size was calculated to test the hypothesis that the prevalence of behavioral risk factors would be lower among students in the schools of the intervention group versus those in the control group at follow-up using G*Power 3.1 (26). With most outcome variables (prevalence of behavioral risk and protective factors) being binary, logistic regression was selected as the statistical test, the required power level was set at 80%, and the significance level pre-specified at α=0.05. The to-be-detected effect was specified at an Odds Ratio = 0.75, which represents the average of pooled effect sizes from a recent meta-analysis (11) considering universal school interventions across a range of behaviors, similar to the ones assessed in our study.

The initial sample size calculation resulted in n=473, which was inflated assuming a design effect of 1.5 and an expected response rate of 90%: 473*1.5/0.9 = 788. Finally, this sample size was quadrupled aiming at reporting for two age groups within both the intervention and control arm: 13-15 and 16-17 years, resulting in a target sample size of n=3,153.

For peer review only

For peer review only - http://bmjopen.bmj.com/site/about/guidelines.xhtml

Participation in the G-SHPPS and GSHS is voluntary. For the GSHS, in line with local policies, consent will be obtained from the students themselves and/or their parents/guardians (see Appendix for consent forms).

Data collection and measures

G-SHPPS and GSHS

The G-SHPPS questionnaire (2021 version) will assess school policies and practices (14) across seven broad topics (Table 1 and Appendix). Exemplary process indicators are presented under the topics in Table 1. School principals of the participating schools will be asked to electronically complete the questionnaire.

Table 1. Questionnaire modules of the Global School Health Policies and Practices Survey (G-SHPPS) and sample indicators.

G-SHPPS module	Sample process indicators (Percentage of schools that…)
School Health Services	• routinely provide as part of the health services offered to students counselling to prevent violence (including intimate partner, violence, sexual violence, gender-based violence, bullying, and gang violence).<br>• routinely provide as part of the health services offered to students administration of recommended immunizations.<br>• routinely provide as part of the health services offered to students support for management of overweight and obesity.
School Physical environment	• provide soap and water at handwashing facilities for students.<br>• have improved sanitation facilities for students.
Food and Nutrition Services	• make sugar-sweetened carbonated soft drinks and other sugar-sweetened drinks available to students through the vending machines, stores, canteens, or snack bars on school premises.<br>• routinely make fruits and vegetables available to students through the school's food and nutrition services.<br>• price healthy foods and beverages in the vending machine, school store, canteen, or snack bar on school premises lower than unhealthy options.
Health Education	• teach health education.<br>• teach sexual and reproductive health and HIV transmission, prevention, and treatment to students.<br>• teach violence and bullying prevention to students.<br>• teach decision-making, problem-solving, goal-setting, and refusal skills to students to help them avoid or reduce health risks.
Physical education	• teach physical education.<br>• teach students as part of physical education the value of physical activity for health, enjoyment, challenge, self-expression, and/or social interaction.<br>• regularly provide recess or other physical activity breaks to students during the school day.

For peer review only - http://bmjopen.bmj.com/site/about/guidelines.xhtml

School Governance and Leadership	• have an official council, committee, or team responsible for implementing health promoting school policies, programs, and activities.<br>• regularly involve and engage parents/caregivers/families and students in developing health promoting school policies.
School Policies and Resources	• have a policy specifically about becoming or continuing to be a Health Promoting School.<br>• have a policy prohibiting use of at least some tobacco or nicotine products, alcohol, and Illicit drugs among students on school premises.<br>• regularly monitor, evaluate, or assess the quality of their health promoting policies, programs, or activities.

Students' health behaviors and protective factors will be assessed using the GSHS questionnaire (2021 version) (15) (see Appendix). The GSHS is a paper-based, self-administered questionnaire with generic answer sheets that students complete during one classroom period.

The GSHS includes ten core questionnaire modules. Table 2 presents these modules with exemplary outcome indicators. Each city will be encouraged to include all modules. However, up to four modules may be excluded if questions of a specific module are deemed too sensitive. Cities may add context-specific questions, not exceeding a total of 80 questions.

Anthropometric measurements of height and weight are taken prior to survey administration. Students receive their measurement results and are asked to subsequently record them on the questionnaire.

Inclusion of the physical activity module will be mandatory to allow for validity testing of some of these questions. Additionally, a new question will be included for validation against the updated WHO physical activity guidelines (10) reading *"During the past 7 days, did you do at least an average of 60 minutes per day of physical activity across the 7 days?"*.

Participating cities will also be encouraged to include the standard GSHS questions on attending school from home during the pandemic, missing classes or school without permission, and on school attendance under the influence of alcohol and of drugs.

For peer review only - http://bmjopen.bmj.com/site/about/guidelines.xhtml

244 Once the questionnaire has been finalized for each city, the same questionnaire will be
245 anonymously administered to all students.

246

247 **Table 2.** Questionnaire modules of the Global School-based Student Health Survey (GSHS) and
248 sample outcome indicators.

GSHS module	Sample outcome indicators, dependent on the city's choice of modules (Percentage of students who…)
Alcohol use	• currently drank alcohol (at least one drink of alcohol on at least one day during the 30 days before the survey).<br>• ever drank so much alcohol that they were really drunk one or more times during their life.<br>• had their first drink alcohol other than a few sips before age 14 years , among students who ever had a drink of alcohol other than a few sips.
Dietary behaviors	• were underweight (<-2SD from median for BMI by age and sex) (measured).<br>• were overweight (>+1SD from median for BMI by age and sex) (measured).<br>• were obese (>+2SD from median for BMI by age and sex) (measured).<br>• usually drank carbonated soft drinks one or more times per day during the 30 days before the survey.
Drug use	• ever used cannabis one or more times during their life.<br>• used drugs before age 14 years for the first time, among students who ever used drugs.
Hygiene	• usually cleaned or brushed their teeth less than one time per day during the 30 days before the survey.<br>• never or rarely washed their hands after using the toilet or latrine during the 30 days before the survey.
Mental health	• seriously considered attempting suicide during the 12 months before the survey.<br>• attempted suicide one or more times during the 12 months before the survey.<br>• did not have any close friends.
Physical activity	• were physically active for a total of at least 60 minutes per day on all 7 days during the 7 days before the survey.<br>• attended physical education classes on three or more days each week during this school year.<br>• spent three or more hours per day during a typical or usual day sitting or lying down (doing such things as watching television, playing computer games, talking with friends, using their mobile phone, traveling in a motor vehicle, napping, or doing other activities sitting or lying down) when not in school or doing homework or sleeping at night.
Protective factors	• missed classes or school without permission on one or more days during the 30 days before the survey.<br>• reported that their parents or guardians most of the time or always understood their problems and worries during the 30 days before the survey.<br>• reported that their parents or guardians most of the time or always really knew what they were doing with their free time during the 30 days before the survey.
Sexual behaviors	• ever had sexual intercourse.<br>• had sexual intercourse before age 14 years for the first time, among students who ever had sexual intercourse.<br>• used a condom during last sexual intercourse, among students who ever had sexual intercourse.
Tobacco use	• currently used any tobacco products (used any tobacco products on at least 1 day during the 30 days before the survey).<br>• currently smoked cigarettes (smoked cigarettes on at least 1 day during the 30 days before the survey).<br>• currently used electronic cigarettes (used e-cigarettes on at least 1 day during the 30 days before the survey).
Violence and unintentional injury	• were in a physical fight one or more times during the 12 months before the survey.<br>• were seriously injured one or more times during the 12 months before the survey.<br>• were bullied during the 12 months before the survey.

249

250 *Physical activity validation study*

For peer review only - http://bmjopen.bmj.com/site/about/guidelines.xhtml

In addition to the GSHS self-reported physical activity, activity data will be collected using the Axivity AX3 accelerometer. This device has previously been used in large-scale population-based (27, 28) and school-based studies (29). The Axivity AX3 records movement, and reports raw acceleration signals in real time, allowing for transparent analysis (30).

From each school in the control group, one class will be randomly selected to participate in testing the validity of questions currently used in the GSHS to assess student's physical activity, sedentary behavior and sleep, and the new question that was added to assess WHO's 2020 physical activity guidelines (10). Every student in the selected classes will be asked to participate (approximately 300 students per city).

Participation in the physical activity validation study is voluntary. Participating students will be asked to provide written assent, and their parents or guardians will be asked to provide written consent (see Appendix).

In the week prior to the GSHS baseline survey, selected students will wear the Axivity AX3 on their non-dominant wrist continuously for eight consecutive days (31). Trained personnel will instruct students to wear the waterproof device and to participate in all their normal activities. After having worn the accelerometer, students will be asked to participate in the GSHS survey at baseline. Axivity AX3 data will then be compared to data collected with the GSHS physical activity module and the added physical activity question.

Interventions

This study's intervention approach is embedded in the framework of the Global Standards for Health Promoting Schools and its concept of a whole-school approach to promoting health (18).

For peer review only - http://bmjopen.bmj.com/site/about/guidelines.xhtml

Schools in the intervention arm will implement health promotion strategies to address the most critical health risk behaviors and protective factors, considering the eight Global Standards including government policies and resources; school policies and resources; school governance and leadership; school and community partnerships; school curriculum; school social-emotional environment; school physical environment; and school health services.

Following the baseline surveys, students in the intervention arm will receive instructions to participate in photovoice (22). This is an established (23, 24) qualitative participatory method through which adolescents can pictorially take impressions of health facilitators and barriers in their school and community. Pictures will be uploaded onto a secure platform, reviewed and sorted by trained personnel, ensuring that no inappropriate photos are included.

To develop the interventions, data-to-action workshops will be held in each city (see Appendix for agenda). They will be facilitated by the local study coordinator and WHO and UNESCO personnel and include a minimum of three nominated students from each intervention school, teachers, parents, and up to five local authorities and researchers. The nomination of students for the workshops will be based on the student's interest in participating and ensure gender balance.

Data-to-action workshops will use the data collected during the baseline surveys, the photovoice pictures, and follow the Global AA-HA! approach and its three key steps (16): First, recognizing that the health needs are different in each city, a needs assessment will be done, using the GSHS data and the photovoice pictures. To facilitate this, for the workshop, data will be visualized in an easy-to-understand summary format, including fact sheets and infographics. Workshop participants will review the site-specific results to identify which conditions, health

For peer review only - http://bmjopen.bmj.com/site/about/guidelines.xhtml

risks and determinants need to be addressed most urgently. Second, considering that the situation adolescents live in is different in each setting, in a landscape analysis, results from the local G-SHPPS will be reviewed, along with other existing programmes, policies and legislation. Similar to step one, results will be summarized in an easy-to-understand format and used to identify gaps and potential areas for improvement. Step two will also include a review of evidence-based interventions of the Global AA-HA! and a meta-analysis (11), prepared in a format that will speak to workshop participants. Third, specific actions will be identified to address the issues specific to each city, considering the outcomes of steps one and two. Identified actions will be prioritized, considering availability of resources, effectiveness, feasibility, capacity to implement, cultural appropriateness, and focus on structural and systems-oriented changes.

This participatory approach will ensure that – while considering the adolescents' health needs and each city's situation – the selected interventions will be based on evidence, be feasible, appropriate, acceptable and aligned with local priorities. For example, a set of interventions to decrease tobacco use might be selected for a city with a high prevalence of tobacco use among students. These might include individual- and interpersonal-level interventions, but also school policy changes such as the introduction of smoke-free school policies that would ultimately lead to sustained behavior change.

The workshop duration will be three days, and include students, parents and teachers for at least two hours per day. To ensure that adolescents are meaningfully engaged, existing guidelines on adolescent participation and civic engagement will be followed (32). Breakout sessions will be organized during the workshops for which the different stakeholders will be

For peer review only - http://bmjopen.bmj.com/site/about/guidelines.xhtml

separated, to ensure that each stakeholder group gets their own space to express their views. The different views will be brought back to the plenary and integrated as appropriate. Students will also get the opportunity to comment on and add to actions proposed by other stakeholders.

Each city will develop an implementation plan based on the prioritized interventions. These will be classified by topic and implementation level, including structural, environmental, organizational, community, interpersonal and individual level.

Monitoring and evaluation

As per the Global AA-HA! (16), each city's implementation plan will include process and outcome monitoring through data collected with the G-SHPPS and the GSHS. Progress in the implementation of interventions will be monitored on a periodic basis. For each agreed upon activity/intervention in the implementation plan, monthly reporting will include whether the activity is not started, in planning, in progress or implemented, with a section for next steps and who is responsible.

Follow-up G-SHPPS and GSHS surveys (the same as at baseline) will occur two years after the baseline surveys in all selected schools. For the GSHS, new classes from these schools will be randomly selected.

Data management and analysis plan

For peer review only - http://bmjopen.bmj.com/site/about/guidelines.xhtml

337 During electronic data collection through DataForm (LimeSurvey) (33), G-SHPPS data are uploaded to a secure server that will only be accessible by the city and WHO. Summary results of key indicators will be produced for each city.

340 GSHS data are collected on computer scannable answer sheets with no personal identifying information. Data will be processed using standard cleaning and generic data analysis programmes. A city-specific database will be generated and results will be displayed on a standard fact sheet including information on response rates, a summary of methods, and weighted prevalence estimates with confidence intervals (CIs) (15).

345 The Axivity AX3 data will be downloaded on-site and uploaded to a secure WHO server with unique identifiers, with no personal information. The unique identifier will be used to link these data to the student's GSHS physical activity data. Time spent in total physical activity, moderate- to vigorous-intensity physical activity, and in sedentary behaviour will be computed from the Axivity AX3 data for each student, using standard methods (34, 35). Agreement between the Axivity AX3 and the questionnaire data will be assessed using approaches consistent with previous studies (31, 36).

352 For GSHS and G-SHPPS follow-up surveys, the same data analysis procedures as at baseline will be used.

354 To assess effectiveness of the interventions, comparative analysis of changes of policies and programmes and of students' health behaviors between the intervention and control group will be undertaken. With most outcome variables being binary, most intervention effects will be estimated through adjusted odds ratios with 95% CIs, using random effects logistic regression to adjust for within-school clustering. Additionally, a score across multiple risk and protective

For peer review only - http://bmjopen.bmj.com/site/about/guidelines.xhtml

behaviors will be computed for each city (depending on the selection of questionnaire modules in each city), and effectiveness of the intervention will also be tested using this score (37).

There will be no imputation for missing data.

The analysis will account for potential spillover effects as much as possible. Spillover effects are defined as benefits provided by interventions that extend beyond direct recipients and impact people in close physical or social proximity who did not directly receive the intervention themselves, namely students in the control arm (38).

Dissemination

Following GSHS and G-SHPPS policy, data will be held at the country level, with a copy at WHO, for an initial two-year period. During this time, each site will be encouraged to produce any report and publication they desire. After this period, datasets will be made publicly available, including in the WHO NCD microdata repository (39). GSHS and G-SHPPS results will be presented in a summary report, including user-friendly infographics, for sharing with the participating students, their parents, teachers and communities. Data and results from the physical activity validation study will be stored on a WHO server and will be made available to interested researchers upon request after the initial two-year period.

Patient and Public Involvement Statement

Relevant local stakeholders will be engaged at all project stages to ensure that this research builds local capacity and is responsive to the health needs and priorities of the students in each city (40). Besides representatives of the national or local health and education authorities and

For peer review only - http://bmjopen.bmj.com/site/about/guidelines.xhtml

local researchers, the study committees include community and school stakeholders. Student participants, parents/guardians, teachers and community leaders will lead the use of the collected survey data to identify and implement interventions. Interventions will additionally be shaped by pictures that students provide of what they perceive as facilitators and barriers to their own health behavior.

DISCUSSION

This study will generate reliable and granular data for students of selected secondary cities for immediate action, and contributing to the knowledge base on what interventions might be effective to improve student health.

In applying a design that has been previously used for similar studies (11, 19, 20) and in using existing global tools and frameworks (16, 18), robustness and consistency of findings will be maximized. For example, with the use of the GSHS questionnaire (15), cities will be able to compare their results to those of previous national surveys in their country. In the intervention planning phase, our approach will also build on existing policies and practices which will be reviewed and used as a foundation for actions.

Our approach to data collection is comprehensive and fills important gaps. The selection of secondary cities ensures that information will be generated for settings that are generally less likely to be the focus and for which greatest knowledge gaps exist. The collection of data not only at the individual level, but also at school-level will allow for better understanding of potential linkages between contextual factors and individual behaviors, which is important information for planning and implementing of actions. Furthermore, this study will strengthen

For peer review only - http://bmjopen.bmj.com/site/about/guidelines.xhtml

403 the global surveillance of physical activity and sedentary behaviors. To our knowledge, it is the
404 first study to validate survey questions on these behaviors against accelerometry across diverse
405 LMIC contexts.

406 In preparation for this study, we also faced challenges. The COVID-19 pandemic led to delays.
407 Due to long periods of school closures in most of the participating cities, travel restrictions and
408 involvement of relevant staff in the pandemic response, planned baseline field work was
409 postponed multiple times. Other potential challenges as we start to implement may include
410 schools not being able to fill the electronic G-SHPPS questionnaire due to lack of computer
411 literacy, resources or connectivity. In these cases, respondents will record answers on paper,
412 and the information will be entered in the electronic system subsequently. Cities may also face
413 barriers to the implementation of prioritized interventions that are beyond their control, such
414 as higher-level policies that cannot be changed. To avoid such situations, potential barriers and
415 scenarios will be discussed during the data-to-action workshops. Finally, during the two-year
416 follow-up period, there might be turnover of relevant stakeholders, such as teachers or
417 community personnel. Therefore, institutional memory and capacity is developed involving
418 several stakeholders from each participating school and community.

419 This study aims at increasing the potential of the prioritized interventions to be effective,
420 feasible, accepted, easy to be implemented and monitored, and sustainable. This can be
421 achieved through several mechanisms which we apply. They include the locally-based data-
422 informed identification of issues to be addressed, the community participatory approach
423 guided by adolescents themselves, the use of existing evidence-based models, the focus on
424 sustainable policy and system approaches in addition to programming, and the multiple

For peer review only - http://bmjopen.bmj.com/site/about/guidelines.xhtml

behavior, multi-disciplinary approach (41, 42, 43). Process evaluation will inform on the progress and outputs of specific intervention actions, whereas outcome evaluation will inform on the efficacy. This ensures lessons learned across intervention actions and across the initiative to inform translation and future dissemination.

Ethics

The master protocol and the four site-specific protocols have been approved by the WHO Research Ethics Review Committee (ERC.0003397). The four site-specific protocols have also been approved by local Ethics Committees. Following standard G-SHPPS procedures, permissions from all schools to take part in the study will be obtained prior to field work. For the GSHS, following standard procedures previously used in over 100 countries, all parents/guardians will be notified about the study, and parental/guardian consent will be obtained following local laws and policies. For the physical activity validation study, all parents/guardians will be notified and parental/guardian consent and/or student assent will be obtained in each city, adhering to local laws and policies.

Trial status

This trial began recruitment in January 2022. Baseline data collection started in the first city (Jaipur; India) in August 2022 and is expected to be finalized in all four cities in February 2023. After the two-year intervention period and the follow-up assessments, the study completion date is expected in January 2026.

For peer review only

Acknowledgements

We thank Prof Susan Sawyer and Prof Antony Morgan for their thorough review of the study protocol.

Competing interests

The authors declare that they do not have competing interests.

Funding

This study is funded by Fondation Botnar, Activity REG-19-015. The funder did not play a role in the design of this study.

Contributors

RG wrote the first draft of this paper. RG and LMR conceptualized the study and developed the methods. LK, LB and SAL contributed to the development of the methods and preparation for sampling and data collection. PA contributed to the development of Table 2 and the methods for the intervention. AO contributed to the development of the methods for the physical activity validation study. CRN, SH, JJ and VB contributed to the development of the methods for the intervention. JA, UA, SC, RBC, CD, HF, HH, PJ, PK, SMM, RM, DO, TO and FZT coordinate site-specific implementation and provided input on the manuscript from a site-specific perspective. All authors have reviewed the paper and approved its final version.

Patient and public involvement

For peer review only - http://bmjopen.bmj.com/site/about/guidelines.xhtml

Patients and/or the public were involved in the design, or conduct, or reporting, or dissemination plans of this research. Refer to the Methods section for further details.

Patient consent for publication

Not applicable.

Authors' information

RG, LK, LB, JA, VB, CD, HF, HH, PJ, PK, SAL, SMM, RM and LMR are members of the World Health Organization. SC, RBC and DO are members of the Pan American Health Organization. The authors alone are responsible for the views expressed in this publication and they do not necessarily represent the decisions, policy, or views of the World Health Organization or the Pan American Health Organization.

Figure title and legend

Figure title

Figure 1. Overview of the study design.

Figure legend

[a] *GSHS - Global School-based Student Health Survey*

[b] *G-SHPPS - Global School Health Policies and Practices Survey*

For peer review only - http://bmjopen.bmj.com/site/about/guidelines.xhtml

REFERENCES

1. World Health Organization. Tackling NCDs. Best buys. Geneva, Switzerland: World Health Organization; 2017.
2. Watkins D, Hale J, Hutchinson B, Kataria I, Kontis V, Nugent R. Investing in non-communicable disease risk factor control among adolescents worldwide: a modelling study. BMJ global health. 2019;4(2):e001335.
3. Ginis KA, Nigg CR, Smith AL. Peer-delivered physical activity interventions: an overlooked opportunity for physical activity promotion. Translational behavioral medicine. 2013;3(4):434-43.
4. Azzopardi P, Kennedy E, Patton G. Data and indicators to measure adolescent health, social development and well-being. Florence, Italy: UNICEF Office of Research - Innocenti; 2017.
5. Neal S, Ruktanonchai C, Chandra-Mouli V, Matthews Z, Tatem AJ. Mapping adolescent first births within three east African countries using data from Demographic and Health Surveys: exploring geospatial methods to inform policy. Reproductive health. 2016;13(1):98.
6. Liang M, Simelane S, Fortuny Fillo G, Chalasani S, Weny K, Salazar Canelos P, et al. The State of Adolescent Sexual and Reproductive Health. The Journal of adolescent health : official publication of the Society for Adolescent Medicine. 2019;65(6s):S3-s15.
7. Ridgers ND, Timperio A, Crawford D, Salmon J. Validity of a brief self-report instrument for assessing compliance with physical activity guidelines amongst adolescents. Journal of science and medicine in sport. 2012;15(2):136-41.
8. Prochaska JJ, Sallis JF, Long B. A physical activity screening measure for use with adolescents in primary care. Archives of pediatrics & adolescent medicine. 2001;155(5):554-9.
9. World Health Organization. Global recommendations on physical activity for health. Geneva, Switzerland: World Health Organization; 2010.
10. World Health Organization. WHO guidelines on physical activity and sedentary behaviour. Geneva, Switzerland: World Health Organization; 2020.
11. MacArthur G, Caldwell DM, Redmore J, Watkins SH, Kipping R, White J, et al. Individual-, family-, and school-level interventions targeting multiple risk behaviours in young people. Cochrane Database Syst Rev. 2018;10(10):CD009927-CD.
12. Greene K. The theory of active involvement: processes underlying interventions that engage adolescents in message planning and/or production. Health communication. 2013;28(7):644-56.
13. Larsson I, Staland-Nyman C, Svedberg P, Nygren JM, Carlsson IM. Children and young people's participation in developing interventions in health and well-being: a scoping review. BMC health services research. 2018;18(1):507.
14. World Health Organization. Global School Health Policies and Practices Survey Geneva, Switzerland: World Health Organization; 2021 [Available from: https://www.who.int/teams/noncommunicable-diseases/surveillance/systems-tools/global-school-health-policies-and-practices-survey.
15. World Health Organization. Global School-based Student Health Survey (GSHS) 2021 [Available from: https://www.who.int/teams/noncommunicable-diseases/surveillance/systems-tools/global-school-based-student-health-survey.
16. World Health Organization. Global Accelerated Action for the Health of Adolescents (AA-HA!): guidance to support country implementation. Geneva, Switzerland; 2017.
17. World Health Organization; United Nations Educational; Scientific and Cultural Organization. Making every school a health-promoting school: implementation guidance. Geneva, Switzerland: World Health Organization; United Nations Educational; Scientific and Cultural Organization; 2021.
18. World Health Organization; United Nations Educational; Scientific and Cultural Organization. Making every school a health-promoting school: global standards and indicators for health-promoting

For peer review only - http://bmjopen.bmj.com/site/about/guidelines.xhtml

schools and systems. Geneva, Switzerland: World Health Organization and United Nations Educational, Scientific and Cultural Organization; 2021. Contract No.: 21 November.
19. Shinde S, Weiss HA, Varghese B, Khandeparkar P, Pereira B, Sharma A, et al. Promoting school climate and health outcomes with the SEHER multi-component secondary school intervention in Bihar, India: a cluster-randomised controlled trial. The Lancet. 2018;392(10163):2465-77.
20. Patton GC, Bond L, Carlin JB, Thomas L, Butler H, Glover S, et al. Promoting social inclusion in schools: a group-randomized trial of effects on student health risk behavior and well-being. Am J Public Health. 2006;96(9):1582-7.
21. Langford R, Bonell C, Jones H, Pouliou T, Murphy S, Waters E, et al. The World Health Organization's Health Promoting Schools framework: a Cochrane systematic review and meta-analysis. BMC Public Health. 2015;15(1):130.
22. PhotoVoice. PhotoVoice - Ethical photography for social change 2021 [Available from: https://photovoice.org/.
23. Fountain S, Hale R, Spencer N, Morgan J, James L, Stewart MK. A 10-Year Systematic Review of Photovoice Projects With Youth in the United States. Health promotion practice. 2021;22(6):767-77.
24. Catalani C, Minkler M. Photovoice: a review of the literature in health and public health. Health education & behavior : the official publication of the Society for Public Health Education. 2010;37(3):424-51.
25. Axivity. Axivity AX3 Newcastle, United Kingdom: Axivity; 2021 [Available from: https://axivity.com/product/ax3.
26. Faul F, Erdfelder E, Buchner A, Lang AG. Statistical power analyses using G*Power 3.1: tests for correlation and regression analyses. Behavior research methods. 2009;41(4):1149-60.
27. Doherty A, Jackson D, Hammerla N, Plötz T, Olivier P, Granat MH, et al. Large Scale Population Assessment of Physical Activity Using Wrist Worn Accelerometers: The UK Biobank Study. PloS one. 2017;12(2):e0169649.
28. Westgate K, Ridgway C, Rennie K, Strain T, Wijndaele K, Brage S. Feasibility of incorporating objective measures of physical activity in the STEPS program. A pilot study in Malawi. Cambridge, United Kingdom: MRC Epidemiology Unit, University of Cambridge; 2020.
29. Bonnesen CT, Toftager M, Madsen KR, Wehner SK, Jensen MP, Rosing JA, et al. Study protocol of the Healthy High School study: a school-based intervention to improve well-being among high school students in Denmark. BMC public health. 2020;20(1):95.
30. Troiano RP, McClain JJ, Brychta RJ, Chen KY. Evolution of accelerometer methods for physical activity research. British journal of sports medicine. 2014;48(13):1019-23.
31. Rääsk T, Mäestu J, Lätt E, Jürimäe J, Jürimäe T, Vainik U, et al. Comparison of IPAQ-SF and Two Other Physical Activity Questionnaires with Accelerometer in Adolescent Boys. PloS one. 2017;12(1):e0169527.
32. UNICEF. Engaged and heard! Guidelines on adolescent participation and civic engagement. New York, USA: UNICEF; 2020.
33. Lime Survey. Professional online surveys with LimeSurvey 2020 [Available from: https://www.limesurvey.org/.
34. Stewart T, Narayanan A, Hedayatrad L, Neville J, Mackay L, Duncan S. A Dual-Accelerometer System for Classifying Physical Activity in Children and Adults. Medicine and science in sports and exercise. 2018;50(12):2595-602.
35. Blythe A, Crouter SE, LaMunion SR. Validity of consumer-based physical activity monitors for estimating energy expenditure in youth. University of Tennessee Honors Thesis Project; 2017.
36. Bland JM, Altman DG. Statistical methods for assessing agreement between two methods of clinical measurement. Lancet (London, England). 1986;1(8476):307-10.

For peer review only - http://bmjopen.bmj.com/site/about/guidelines.xhtml

37. Prochaska JJ, Velicer WF, Nigg CR, Prochaska JO. Methods of quantifying change in multiple risk factor interventions. Preventive medicine. 2008;46(3):260-5.
38. Benjamin-Chung J, Arnold BF, Berger D, Luby SP, Miguel E, Colford JM, Jr., et al. Spillover effects in epidemiology: parameters, study designs and methodological considerations. International journal of epidemiology. 2018;47(1):332-47.
39. World Health Organization. NCD Microdata Repository: World Health Organization; 2021 [Available from: https://extranet.who.int/ncdsmicrodata/index.php/home.
40. Countil for International Organizations of Medical Sciences (CIOMS). International Ethical Guidelines for Health-related Research Involving Humans. Geneva, Switzerland; 2016.
41. Thompson MA, Nigg CR. Effect of an adolescent peer-led health curriculum on peer educators and participants. Health Education Journal. 2020;80(3):337-50.
42. Nigg C, Geller K, Adams P, Hamada M, Hwang P, Chung R. Successful dissemination of Fun 5 - a physical activity and nutrition program for children. Translational behavioral medicine. 2012;2(3):276-85.
43. Braun KL, Nigg CR, Fialkowski MK, Butel J, Hollyer JR, Barber LR, et al. Using the ANGELO model to develop the children's healthy living program multilevel intervention to promote obesity preventing behaviors for young children in the U.S.-affiliated Pacific Region. Childhood obesity (Print). 2014;10(6):474-81.

For peer review only

For peer review only - http://bmjopen.bmj.com/site/about/guidelines.xhtml

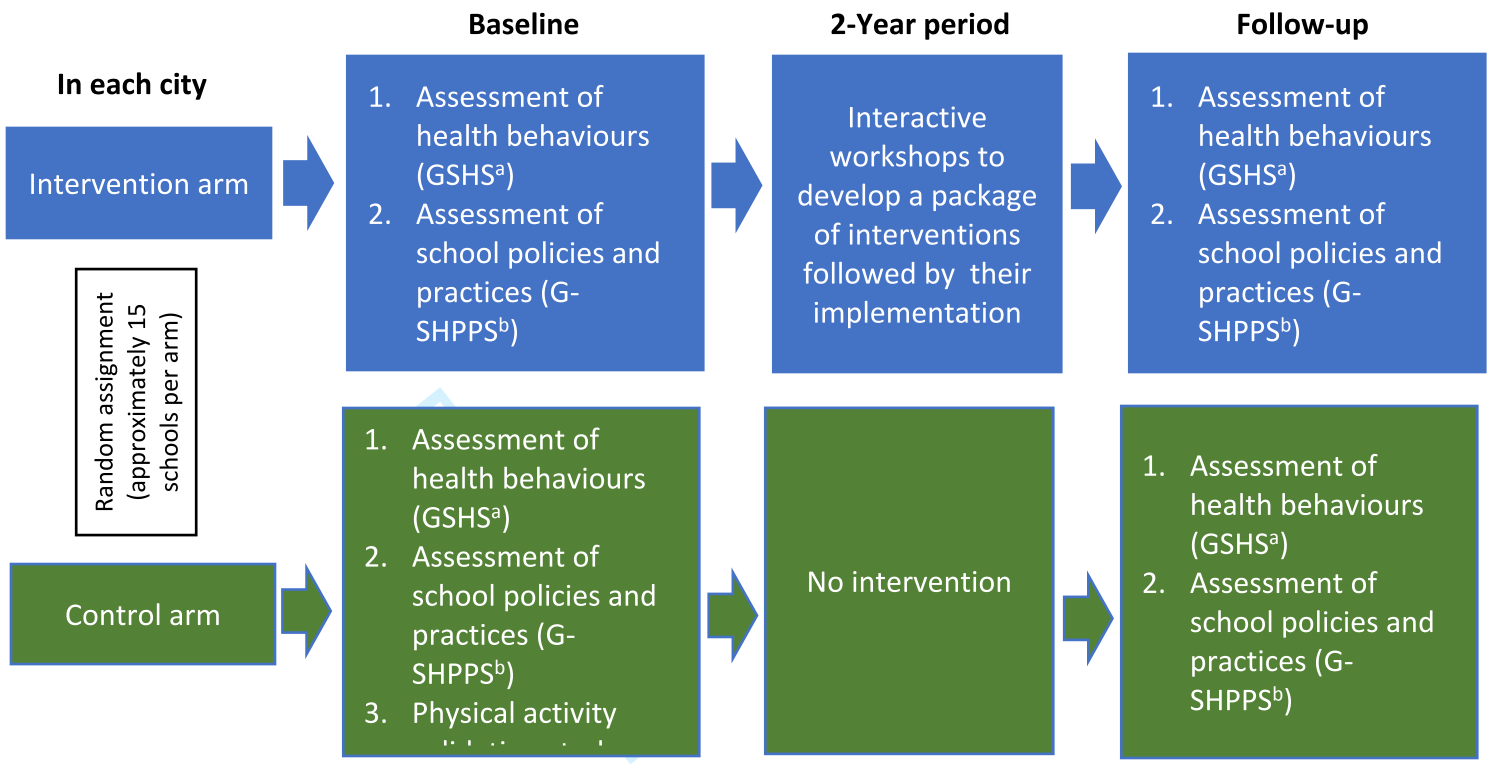

[a] GSHS - Global School-based Student Health Survey
[b] G-SHPPS - Global School Health Policies and Practices Survey

Figure 1. Overview of the study design.

For peer review only

Appendix

G-SHPPS questionnaire

GSHS questionnaire

Notification, permission and assent forms

Data-to-action workshop agenda

For peer review only

Global School Health Policies and Practices Survey (G-SHPPS)

2021

This questionnaire will be used to assess school health policies and practices across our country. Your cooperation is essential for making the results of this survey comprehensive, accurate, and timely. Your answers will be kept confidential. Individual school results will not be reported.

INSTRUCTIONS

1. This questionnaire should be completed by the principal or headteacher (or the person acting in that capacity) and concerns only activities that occur in this school and local community. Please feel free to consult with other people (such as a health education teacher or school nurse) if you are not sure of an answer.

2. For the purposes of this questionnaire, a “policy” or “school policy” is any **written** law, rule, regulation, administrative order, guideline, standard, or similar kind of mandate issued or created **by this school or a local, state, district, or federal agency or organization with authority over this school**. Schools may sometimes grant policy exceptions or waivers, but please answer each question based on what is considered the general policy and standard practice in this school.

3. For the purposes of this questionnaire, “health” is defined as a state of complete physical, mental, and social well-being and not merely the absence of disease or infirmity.

4. Follow the instructions for each question. If you are unsure about the meaning of a word or phrase that is bolded with an asterisk you will find a definition for it at the end of that question.

5. You may move forward and backward through the questionnaire by using the navigation buttons in the questionnaire. Do not use the forward and backward arrows on your browser.

6. If you need to stop responding and resume later, your responses will be saved. Just press the **Resume later** link at the bottom of the page before closing your browser. To resume please use the same URL or token provided initially to finalize your questionnaire.

7. Once you are comfortable with all your responses, click on the SUBMIT button at the end of the questionnaire.

8. Please answer every question.

9. If you have any questions about this survey, please contact the person identified on the email you were sent about this survey.

THANK YOU FOR YOUR ASSISTANCE.

For peer review only - http://bmjopen.bmj.com/site/about/guidelines.xhtml

Table of Contents

	Page
**1. School Overview**	3
**2. School Health Services**	4
School health workers	4
Health services for students	5
Health information management	9
School entry requirements	9
**3. School Physical Environment**	10
Water, sanitation, and hygiene	10
Injury prevention and safety	11
Sun safety	11
Pest control	12
**4. Food and Nutrition Services**	13
Food preparation	14
Other foods and beverages available at school	15
Food and nutrition services environment	16
**5. Health Education**	17
Content of health education	18
**6. Physical Education**	20
Content of physical education	21
Facilities and equipment	21
Physical activity or recreation clubs and competitive sports	22
**7. School Governance and Leadership**	23
School health councils, committees, or teams	23
Community partnerships	24
**8. School Policies and Resources**	25
Overall curriculum	25
Professional development for teachers	26
Bullying and violence prevention	26
Tobacco use prevention	27
Alcohol use prevention	27
Illicit drug use prevention	28
Crisis preparedness and emergency response	28
Eye health	29
Pregnancy students	29

For peer review only - http://bmjopen.bmj.com/site/about/guidelines.xhtml

1. School Overview

1-01. Who attends this school?

A. All genders
B. Only males
C. Only females

1-02. How many students are enrolled in this school? _____

1-03. How many full-time teachers provide instruction to students in this school? _____

1-04. Are the following educational levels taught in this school? *(Mark yes or no for each level.)*

	Yes	No
A. Primary education (**ISCED 1**[1])	O	O
B. Lower secondary education (ISCED 2)	O	O
C. Upper secondary education (ISCED 3)	O	O

1-05. Is this a boarding school where students both learn and live?

A. Yes
B. No

1-06. How would you classify this school?

A. Government or public school
B. Non-government, religious, or private school
C. None of the above

1-07. Is this school located in a mostly rural or mostly urban setting?

A. Mostly rural
B. Mostly urban

1-08. Does this school have internet service reliable enough that it can be routinely used for student instruction?

A. Yes
B. No

[1] A widely-used global reference classification for education systems that is maintained and periodically revised by the UNESCO Institute for Statistics in consultation with Member States and other international and regional organizations. The International Standard for Classification of Education (ISCED) allows comparison of education systems across countries.

For peer review only - http://bmjopen.bmj.com/site/about/guidelines.xhtml

2. School Health Services

2-01. Which of the following statements **best** describes how **health services**[2] are regularly provided to students in this school?

A. Health services are provided both on school premises and at separate facilities (not on school premises) through a formal agreement with this school
B. Health services are provided only on school premises
C. Health services are provided only at separate facilities (not on school premises) through a formal agreement with this school to provide health services
D. Health services are only provided on an emergency basis **(Go to 2-15)**
E. I do not know **(Go to 2-15)**

2-02. Which of the following statements best describes how school policy addresses health services for students?

A. School policy provides a specific plan or guidelines for implementing health services for students
B. Health services are mentioned in a school policy, but there is no specific plan or guideline for implementing health services for students
C. Health services for students are not addressed in any school policy
D. I do not know

2-03. Does this school have someone officially responsible for managing or coordinating this school's health services?

A. Yes
B. No
C. I do not know

2-04. Does this school have the supplies needed for teachers and other school staff to apply **standard or universal precautions**[3], including disposable gloves and bandages?

A. Yes
B. No
C. I do not know

School health workers

2-05. Do the following types of **health workers**[4] provide health services to students in this school? *(Mark yes or no or I do not know for each type of health worker.)*

	Yes	No	I do not know
A. Nurses	O	O	O
B. Physicians	O	O	O
C. Psychologists, counsellors, or social workers	O	O	O
D. Dentists or dental hygienists	O	O	O
E. Nutritionists	O	O	O

---

[2] Services provided by a health worker to students enrolled in primary or secondary education, either on school premises or in a facility not on school premises that has a formal agreement with the school to provide health services to the school's students.
[3] A method of infection control in which all human blood, certain body fluids, and fresh tissues and cells of human origin are handled as if they are known to be infected with Human Immunodeficiency Virus (HIV), Hepatitis B Virus (HBV), and/or other blood-borne pathogens.
[4] A health worker is a person whose main function is to deliver health promotion, prevention, care, and/or treatment services to students, such as a nurse or clinical psychologist, but not a teacher.

2-06. On average, how many days per week is a health worker available to students in this school?

A. Less than 1 day
B. 1 day
C. 2 days
D. 3 days
E. 4 days
F. 5 or more days
G. I do not know

2-07. Does this school provide professional development opportunities to health workers to improve the quality of health services for students?

A. Yes
B. No
C. I do not know

Health services for students

2-08. As part of the health services offered to students, are the following **preventive interventions**[5] routinely provided? *(Mark yes or no or I do not know for each preventive intervention.)*

	Yes	No	I do not know
A. Routine preventive health check-ups	O	O	O
B. Administration of recommended immunizations (such as **diphtheria, HPV, tetanus, measles, and rubella**[6])	O	O	O
C. **Micronutrient**[7] supplements (such as iron, iodine, zinc, or Vitamin A)	O	O	O

2-09. Does this school provide information to students, parents, caregivers, and families about the value and importance of receiving routine immunizations to prevent infectious diseases?

A. Yes
B. No
C. I do not know

2-10. As part of the health services offered to students, are the following **screenings**[8] routinely provided for at least most students? *(Mark yes or no or I do not know for each screening.)*

	Yes	No	I do not know
A. Eye and vision	O	O	O
B. Ear and hearing	O	O	O
C. Oral health	O	O	O
D. Nutrition (such as for **anaemia, malnutrition, and obesity**[9]**)**	O	O	O

Note: If A, B, C, and D are all No or I do not know – Go to 2-12.

[5] A health intervention to prevent illness, disease, or injury.
[6] Diphtheria – A serious bacterial infection of the nose and throat. HPV – A sexually transmitted infection caused by the human papillomavirus that may lead to genital warts or cancer. Tetanus – A bacterial infection that causes painful muscle contractions particularly in the jaw and neck. Measles – A highly contagious viral disease characterized by a high fever and rash. Rubella – A viral disease characterized by a low fever, sore throat, and rash.
[7] Vitamins and minerals vital to healthy development, disease prevention, and well-being.
[8] Medical tests to check for diseases and health conditions before there are any signs or symptoms.
[9] Anaemia – A condition marked by a lack of red blood cells or of hemoglobin in the blood. Malnutrition – Lack of proper nutrition caused by not having enough to eat, not eating enough of the right things, or another disease. Obesity – A disorder involving excess body fat that increases the risk of other health problems.

For peer review only - http://bmjopen.bmj.com/site/about/guidelines.xhtml

2-11. Do the following actions typically occur when a student's screening indicates a potential problem? *(Mark yes or no or I do not know for each action.)*

	Yes	No	I do not know
A. The student is notified	O	O	O
B. The student's parents or caregiver are notified	O	O	O
C. The student's teachers are notified	O	O	O
D. A referral is provided for an appropriate health worker	O	O	O

2-12. As part of the health services offered to students, are the following **health promotion**[10] activities routinely provided? *(Mark yes or no or I do not know for each health promotion activity.)*

	Yes	No	I do not know
A. Timely care-seeking from an appropriate provider	O	O	O
B. Increased health literacy	O	O	O
C. Improved personal hygiene and handwashing with soap	O	O	O
D. Improved oral health care	O	O	O
E. Reduced consumption of sugar and **sugar-sweetened**[11] beverages	O	O	O
F. Increased physical activity and limited **sedentary behaviour**[12]	O	O	O
G. Appropriate use of electronic devices	O	O	O
H. Attainment of adequate sleep	O	O	O
I. Appropriate sun exposure for the context	O	O	O
J. Menstrual hygiene management	O	O	O

Note: Ask J only if females attend this school.

review only

[10] The process of enabling individuals to increase control over, and to improve, their health. Health promotion can happen formally or informally, in a group or one-on-one, and in a clinical setting.

[11] Sugar-sweetened beverages include carbonated soft drinks, sports drinks, energy drinks,100% fruit juices, fruit drinks that are not 100% juice, sugar-sweetened flavoured milks, and sugar-sweetened teas, coffees, or flavoured waters.

[12] Activities occurring in a reclining, seated, or lying position requiring very low energy expenditure.

For peer review only - http://bmjopen.bmj.com/site/about/guidelines.xhtml

2-13. As part of the health services offered to students, are the following health services in each health area routinely provided to students? Health services may include a clinical assessment and subsequent care and support or referral to another facility for care. *(Mark yes or no or I do not know for each health service.)*

	Yes	No	I do not know
**General/cross cutting**			
A. Provision of first aid	O	O	O
B. Administration of over-the-counter and prescribed medications	O	O	O
C. Control and management of pain (such as from a headache or toothache)	O	O	O
D. Management of non-specific symptoms (such as diarrhoea or fever)	O	O	O
**Positive health development**			
E. Identification of developmental difficulties and disabilities	O	O	O
F. Counselling related to physical and psychosocial development (such as puberty, skin changes, body image, or child marriage)	O	O	O
**Unintentional injury**			
G. Management of common childhood injuries (such as fractures or wounds)	O	O	O
H. Management of burns	O	O	O
I. Management of non-fatal drowning and related complications	O	O	O
**Violence**			
J. Counselling to prevent violence (including intimate partner violence, sexual violence, gender-based violence, bullying, and gang violence)	O	O	O
K. Support for victims of violence	O	O	O
**Sexual and reproductive health, including HIV**			
L. Contraceptive counselling	O	O	O
M. Counselling on prevention of HIV or sexually-transmitted infections	O	O	O
N. **HIV pre-exposure and/or post-exposure prophylaxis**[13]	O	O	O
O. HIV testing services	O	O	O
P. Management of sexually-transmitted infections	O	O	O
Q. Management of pregnancy (including the option to continue or return to school)	O	O	O

Note: Ask Q only if females attend this school.

[13] Medicine taken to prevent getting HIV either before or after high risk behaviour.

For peer review only - http://bmjopen.bmj.com/site/about/guidelines.xhtml

2-14. As part of the health services offered to students, are the following health services in each health area routinely provided to students? Health services may include a clinical assessment and subsequent care and support or referral to another facility for care. *(Mark yes or no or I do not know for each health service.)*

	Yes	No	I do not know
**Communicable disease**			
A. Management of common infections (such as ear, eye, dental, skin, throat, or urinary tract infections)	O	O	O
B. Management of other infectious diseases (such as **cholera, dengue, dysentery, helminths, tuberculosis, or malaria**[14*])	O	O	O
C. Support for chronic care of HIV-infected children	O	O	O
**Noncommunicable disease, physical disability, and nutrition**			
D. Management of **anaemia**[15] (such as iron supplementation)	O	O	O
E. Support for management of overweight and **obesity**[16]	O	O	O
F. Management of asthma	O	O	O
G. Management of other chronic conditions (such as developmental disabilities, diabetes, heart disease, or seizures)	O	O	O
H. Rehabilitation, assistive technology, and other services for injured or disabled children	O	O	O
**Mental health, substance use, and self-harm**			
I. Assessment of risk factors and health behaviours (such as tobacco use, drug use, or physical inactivity)	O	O	O
J. Counselling on tobacco, alcohol, and other substance use	O	O	O
K. Short-term counselling or crisis intervention focused on mental health or situational concerns (such as grief or difficult transitions)	O	O	O
L. Management of common behavioural disorders in children (such as ADHD)	O	O	O
M. Management of emotional, anxiety, and depressive disorders	O	O	O
N. Management of eating disorders (such as **anorexia or bulimia**[17])	O	O	O
O. Stress management	O	O	O
P. Management of self-harm and/or suicide risk	O	O	O
Q. Management of **somatoform disorders**[18] and other psychosomatic conditions	O	O	O
R. Management of psychotic disorders	O	O	O
S. Management of harmful use of, dependence on, or withdrawal from a substance (such as alcohol or illicit drugs)	O	O	O

only

[14] Cholera – A bacterial disease causing acute diarrhoea and dehydration. Dengue – A viral disease spread through the bite of a certain type of infected mosquito. Dysentery – A bacterial disease causing inflammation of the intestines and bloody diarrhoea. Helminths – A parasitic worm that causes disease. Tuberculosis – A bacterial infection that mainly effects the lungs. Malaria – A serious and sometimes fatal disease caused by a parasite that infects a certain type of mosquito that feeds on humans.

[15] Anaemia – A condition marked by a lack of red blood cells or of hemoglobin in the blood.

[16] Obesity – A disorder involving excess body fat that increases the risk of other health problems.

[17] Anorexia – An eating disorder characterized by a very low body weight, an intense fear of gaining weight, and a distorted perception of weight. Bulimia - An eating disorder marked by binging followed by methods to avoid weight gain.

[18] Physical symptoms that suggest illness or injury, but which cannot be explained fully by a general medical condition or by the direct effect of a substance.

For peer review only - http://bmjopen.bmj.com/site/about/guidelines.xhtml

Health information management

2-15. Are the following types of health information recorded or kept on file in hard copy or electronic format for students in this school? *(Mark yes or no or I do not know for each type of health information.)*

	Yes	No	I do not know
A. Emergency contact information	O	O	O
B. Physical health history	O	O	O
C. Mental health history	O	O	O
D. Screening results	O	O	O
E. Vaccination history	O	O	O
F. Food allergy or other allergy information	O	O	O
G. Disabilities or special learning needs	O	O	O

2-16. Are the following types of data routinely collected from all students that attend this school? *(Mark yes or no or I do not know for each type of data.)*

	Yes	No	I do not know
A. Student health risk behaviours	O	O	O
B. Student injuries or illnesses that occur at school	O	O	O
C. Student use of school health services	O	O	O
D. Student perception of the quality of school health services	O	O	O
E. Reasons why students are absent from school	O	O	O

Note: If A, B, C, D, and E are all No or I do not know - Go to 2-18.

2-17. Does this school have an established process for routinely reviewing these data collected from students to help develop and implement school policies, programs, or activities?

A. Yes
B. No
C. I do not know

2-18. Does this school report any **notifiable diseases**[19] or infectious disease outbreaks that occur at school among students to the federal, state, or local ministry of health?

A. Yes
B. No
C. I do not know

School entry requirements

2-19. Does this school have a policy requiring students to be in compliance with the **National Immunization Schedule**[20] prior to enrollment in this school?

A. Yes
B. No
C. Not applicable. This country does not have a National Immunization Schedule.
D. I do not know

2-20. Does this school require students to have a preventive health check-up prior to enrollment in this school?

A. Yes
B. No
C. I do not know

[19] A notifiable disease is any disease that is required by law to be reported to government authorities.
[20] A National Immunization Schedule is a list of vaccinations, including the timing of all doses, which may be either recommended or compulsory, depending on the country

For peer review only - http://bmjopen.bmj.com/site/about/guidelines.xhtml

3. School Physical Environment

Water, sanitation, and hygiene

3-01. Which of the following statements **best** describes the **current** service level of this school's handwashing facilities for students?

A. Soap and water are available at handwashing facilities for students
B. Only water, but no soap, is available at handwashing facilities for students
C. No water is available at handwashing facilities for students
D. No handwashing facilities for students are available
E. I do not know

3-02. Which of the following statements **best** describes the **current** service level of this school's drinking water for students?

A. Drinking water for students is available from an **improved source**[21]
B. Drinking water for students comes from an improved source, but is not available
C. Drinking water for students is available from an **unimproved source**[22]
D. No drinking water for students is available
E. I do not know

3-03. Which of the following statements **best** describes the **current** service level of this school's sanitation facilities (such as toilets or latrines) for students?

A. Students have access to **improved sanitation facilities**[23] that are single sex, functional, and private
B. Students have access to improved sanitation facilities, but they are not single sex, they are not functional, or they are not private
C. Students only have access to **unimproved sanitation facilities**[24]
D. No sanitation facilities for students are available **(Go to 3-06)**
E. I do not know **(Go to 3-06)**

3-04. Does this school have sanitation facilities (such as toilets or latrines) that are accessible to students with physical disabilities?

A. Yes
B. No
C. I do not know

3-05. Are the school's sanitation facilities (such as toilets or latrines) usually cleaned daily when this school is in session?

A. Yes
B. No
C. I do not know

[21] Improved drinking water sources includes sources that, by nature of their construction or through active intervention, are protected from outside contamination, particularly fecal matter. These include piped water in a dwelling, plot, or yard; public standpipe; borehole; protected dug well; protected spring; and rainwater collection.
[22] Unimproved drinking water sources include unprotected dug well, unprotected spring, cart with small tank/drum, tanker truck, and surface water (river, dam, lake, pond, stream, canal, and irrigation channels).
[23] Improved sanitation facilities include a connection to a public sewer system, connection to a septic system, pour-flush latrine, and a ventilated improved pit latrine.
[24] Unimproved sanitation facilities include a public or shared latrine, an open pit latrine, and a bucket latrine.

For peer review only - http://bmjopen.bmj.com/site/about/guidelines.xhtml

3-06. Is garbage usually removed daily from the school premises when this school is in session?

A. Yes
B. No
C. I do not know

3-07. Does this school provide sanitary napkins or other menstrual supplies to students?

A. Yes, for free
B. Yes, for a fee
C. No
D. I do not know

Note: Ask this question only if females attend this school.

Injury prevention and safety

3-08. Are the following places or types of equipment routinely inspected for safety issues and hazards? *(Mark yes or no or I do not know for each place or type of equipment or mark not applicable (N/A) if this school does not have the place or type of equipment.)*

	Yes	No	I do not know	N/A
A. Playground equipment	O	O	O	O
B. Sports facilities	O	O	O	O
C. Fire extinguishers or other equipment to extinguish a fire (such as buckets of sand)	O	O	O	O
D. School structures and buildings	O	O	O	O
E. School grounds	O	O	O	O
F. Water source	O	O	O	O

3-09. Does this school have the following features to promote safety among students, including students with disabilities? *(Mark yes or no or I do not know for each feature.)*

	Yes	No	I do not know
A. Handrails on stairs	O	O	O
B. Ramps to facilitate change of level	O	O	O
C. Assistive devices (such as wheelchairs, wider doorways, specialized software, or adaptive switches or knobs)	O	O	O
D. Signage or paint to address vision impairments	O	O	O

Sun safety

3-10. Does this school use the following strategies to reduce sun exposure among students? *(Mark yes or no or I do not know for each strategy.)*

	Yes	No	I do not know
A. Schedule outdoor activities to avoid peak sun intensity	O	O	O
B. Provide shade areas	O	O	O
C. Encourage students to wear hats, other head coverings, or protective clothing when they are outside	O	O	O
D. Encourage students to wear sunscreen or other skin protection products when they are outside	O	O	O

For peer review only - http://bmjopen.bmj.com/site/about/guidelines.xhtml

Pest control

3-11. Are the school grounds where students play or exercise surrounded by a fence to keep out stray or roaming animals?

A. Yes
B. No
C. Not applicable. Stray or roaming animals are not a problem on school grounds
D. I do not know

3-12. Are procedures routinely implemented to control common pests (such as rodents, insects, or snakes) on school grounds?

A. Yes
B. No
C. I do not know

For peer review only

For peer review only - http://bmjopen.bmj.com/site/about/guidelines.xhtml

4. Food and Nutrition Services

4-01. Does this school provide **food and nutrition services**[25] to students?

A. Yes
B. No **(Go to 4-13)**
C. I do not know **(Go to 4-13)**

4-02. Which of the following statements **best** describes how school policy addresses food and nutrition services for students?

A. School policy provides a specific plan or guidelines for implementing food and nutrition services for students
B. Food and nutrition services are mentioned in a school policy, but there is no specific plan or guideline for implementing food and nutrition services for students
C. Food and nutrition services for students are not addressed in any school policy
D. I do not know

4-03. Does this school have someone officially responsible for managing or coordinating this school's food and nutrition services?

A. Yes
B. No
C. I do not know

4-04. Are the following meals or snacks routinely made available to students as part of this school's food and nutrition services? *(Mark yes or no or I do not know for each meal or snack.)*

	Yes	No	I do not know
A. Breakfast	O	O	O
B. Lunch	O	O	O
C. Evening meal	O	O	O
D. Snack(s)	O	O	O
E. Take-home rations	O	O	O

4-05. Which of the following statements **best** describes which students receive free or deeply discounted food as part of this school's food and nutrition services?

A. All students receive free or deeply discounted food
B. Some students receive free or deeply discounted food as determined by some kind of **social means testing**[26]* or other quantitative criteria
C. No students receive free or deeply discounted food
D. I do not know

4-06. Does this school routinely conduct the following activities as part of its food and nutrition services for students? *(Mark yes or no or I do not know for each activity.)*

	Yes	No	I do not know
A. Collect suggestions from students, families, teachers, or other school staff on nutritious food and beverage preferences	O	O	O
B. Provide information to students or families on the nutrition and caloric content of foods and beverages	O	O	O
C. Work with local businesses to enhance the quality or variety of foods offered as part of this school's food and nutrition services	O	O	O

[25] The provision of food support either in the form of actual food or cash/vouchers/stipends for students to buy food available on school grounds.
[26] Assessment using set criteria to identify students who should receive school meals at no cost or at a deeply discounted price.

For peer review only - http://bmjopen.bmj.com/site/about/guidelines.xhtml

4-07. Are the following foods and beverages routinely made available to students through the school's food and nutrition services. *(Mark yes or no or I do not know for each food or beverage.)*

	Yes	No	I do not know
A. Fruit	O	O	O
B. Vegetables	O	O	O
C. Milk or milk products	O	O	O
D. Foods high in fiber such as whole grains, legumes, or nuts	O	O	O
E. Foods high in sugar including cookies, pastries, or other baked goods	O	O	O
F. Salty foods such as chips or crackers	O	O	O
G. Chocolate or other candy	O	O	O
H. Fried foods or other foods high in unhealthy fats	O	O	O
I. Sugar-sweetened carbonated soft drinks	O	O	O
J. Sports and energy drinks	O	O	O
K. 100% fruit or vegetable juices	O	O	O
L. Fruit or vegetable drinks that are not 100% juice	O	O	O
M. Sugar-sweetened flavoured milks	O	O	O
N. Sugar-sweetened teas, coffees, or flavoured waters	O	O	O
O. Water	O	O	O

Food preparation

4-08. Does this school have a policy describing how food served as part of the food and nutrition services should be prepared (in the school or elsewhere) to maximize its nutritional quality?

A. Yes
B. No **(Go to 4-13)**
C. I do not know **(Go to 4-13)**

4-09. Are the following nutrition objectives specifically addressed by this policy? *(Mark yes or no or I do not know for each nutrition objective.)*

	Yes	No	I do not know
A. Reduce saturated or trans-fat intake	O	O	O
B. Reduce salt intake	O	O	O
C. Reduce sugar intake	O	O	O
D. Increase fruit and vegetable consumption	O	O	O
E. Increase consumption of whole grains, legumes, and nuts	O	O	O
F. Increase **micronutrient**[27] intake	O	O	O

4-10. Are any foods served as part of this school's food and nutrition services fortified with **micronutrients**[27] (such as iron, iodine, zinc, or Vitamin A)?

A. Yes
B. No
C. I do not know

4-11. Are the foods that are part of this school's food and nutrition services prepared in the following locations? *(Mark yes or no or I do not know for each location.)*

	Yes	No	I do not know
A. On school grounds	O	O	O
B. Off-site in a centralized (not private) facility	O	O	O
C. Off-site in a private facility, such as a caterer	O	O	O

[27] Vitamins and minerals vital to healthy development, disease prevention, and well-being.

For peer review only - http://bmjopen.bmj.com/site/about/guidelines.xhtml

4-12. Does this school have a policy requiring food preparation staff (on school grounds or off-site) to follow safe food handling practices, such as **WHO's 5 Keys to Safer Food**[28]?

A. Yes
B. No
C. I do not know

Other foods and beverages available at school

4-13. May students purchase food or beverages from a vending machine, school store, canteen, or snack bar on school premises?

A. Yes
B. No **(Go to 4-17)**
C. I do not know **(Go to 4-17)**

4-14. Are healthy foods and beverages in the vending machine, school store, canteen, or snack bar on school premises priced lower than unhealthy options to encourage students to purchase the healthy ones?

A. Yes
B. No
C. I do not know

4-15. Does this school have a policy banning the sale of **sugar-sweetened beverages**[29] to students?

A. Yes
B. No
C. I do not know

4-16. Are the following foods and beverages routinely made available to students through the vending machines, stores, canteens, or snack bars on school premises. *(Mark yes or no or I do not know for each food or beverage.)*

	Yes	No	I do not know
A. Fruit	O	O	O
B. Vegetables	O	O	O
C. Milk or milk products	O	O	O
D. Foods high in fiber such as whole grains, legumes, or nuts	O	O	O
E. Foods high in sugar including cookies, pastries, or other baked goods	O	O	O
F. Salty foods such as chips or crackers	O	O	O
G. Chocolate or other candy	O	O	O
H. Fried foods or other foods high in unhealthy fats	O	O	O
I. Sugar-sweetened carbonated soft drinks	O	O	O
J. Sports and energy drinks	O	O	O
K. 100% fruit or vegetable juices	O	O	O
L. Fruit or vegetable drinks that are not 100% juice	O	O	O
M. Sugar-sweetened flavoured milks	O	O	O
N. Sugar-sweetened teas, coffees, or flavoured waters	O	O	O
O. Water	O	O	O

[28] WHO's 5 Keys to Safer Food are clean, separate raw and cooked, cook thoroughly, keep food at safe temperatures, and use safe water and raw materials.

[29] Sugar-sweetened beverages include carbonated soft drinks, sports drinks, energy drinks, 100% fruit juices, fruit drinks that are not 100% juice, sugar-sweetened flavoured milks, and sugar-sweetened teas, coffees, or flavoured waters.

For peer review only - http://bmjopen.bmj.com/site/about/guidelines.xhtml

Food and nutrition services environment

4-17. Does this school have a policy that students may bring food from home to eat during the school day?

A. Yes
B. No
C. I do not know

4-18. Does this school have a policy that students may bring water from home to drink during the school day?

A. Yes
B. No
C. I do not know

4-19. Where do most students **usually** eat meals during the school day?

A. In their regular classroom
B. In a dedicated eating space, such as a school cafeteria, dining hall, or other special room besides their regular classroom
C. Outside on school grounds
D. Somewhere else
E. Students do not eat meals during the school day
F. I do not know

4-20. Does this school have a policy prohibiting advertisements or promotions, including sponsorships, for candy, **sugar-sweetened beverages**[30], or other unhealthy foods and beverages on school premises?

A. Yes
B. No
C. I do not know

For peer review only

[30] Sugar-sweetened beverages include carbonated soft drinks, sports drinks, energy drinks, 100% fruit juices, fruit drinks that are not 100% juice, sugar-sweetened flavoured milks, and sugar-sweetened teas, coffees, or flavoured waters.

For peer review only - http://bmjopen.bmj.com/site/about/guidelines.xhtml

5. Health Education

5-01. Does this school teach **health education**[31]?

A. Yes
B. No **(Go to 6-01)**
C. I do not know **(Go to 6-01)**

5-02. Which of the following statements **best** describes how health education instruction occurs in this school?

A. Health education instruction occurs only in a regular classroom setting
B. Health education instruction occurs only through extra-curricular activities (such as after school, during lunch, or via clubs or school assemblies)
C. Health education instruction occurs both in a regular classroom setting and through extra-curricular activities
D. I do not know

5-03. Do students receive a grade for health education?

A. Yes
B. No
C. I do not know

5-04. Does this school have someone officially responsible for managing or coordinating this school's health education?

A. Yes
B. No
C. I do not know

5-05. Who provides **most** of the health education instruction in this school?

A. A health education teacher or specialist
B. A nurse or some other kind of health worker
C. Someone else
D. I do not know

5-06. Are the following materials provided to teachers in this school to guide health education instruction? *(Mark yes or no or I do not know for each material.)*

	Yes	No	I do not know
A. Learning outcomes or objectives	O	O	O
B. A planned progression of lesson plans or learning strategies and experiences	O	O	O
C. Teaching and learning resources and content	O	O	O
D. Assessment tools	O	O	O

[31] Any combination of learning experiences designed to help students improve their health by increasing their knowledge, influencing motivation, and improving health literacy.

For peer review only - http://bmjopen.bmj.com/site/about/guidelines.xhtml

5-07. Which of the following statements **best** describes how school policy addresses teaching **skills-based, participatory health education**[32]?

A. School policy provides a specific plan or guidance on teaching skills-based participatory health education
B. School policy mentions health education, but not teaching skills-based participatory health education
C. School policy does not mention health education
D. I do not know

5-08. Does this school routinely provide professional development opportunities for teachers to improve the quality of health education instruction?

A. Yes
B. No
C. I do not know

Content of health education

5-09. Are the following skills taught to students in this school to help them avoid or reduce health risks? *(Mark yes or no or I do not know for each skill.)*

	Yes	No	I do not know
A. How to access high quality health information, products, or services	O	O	O
B. Interpersonal communication	O	O	O
C. Decision-making	O	O	O
D. Problem-solving	O	O	O
E. Goal-setting	O	O	O
F. Refusal	O	O	O
F. Coping or stress management	O	O	O
G. Hand washing with soap	O	O	O
H. Tooth brushing with fluoride toothpaste	O	O	O
I. Using the internet and social media safely	O	O	O
J. Advocating for personal, family, or community health and wellbeing	O	O	O

[32] Skills-based, participatory health education includes strategies and techniques focused on skill development that encourage students to become actively involved in their learning process.

For peer review only - http://bmjopen.bmj.com/site/about/guidelines.xhtml

5-10. Are the following health topics taught to students in this school? *(Mark yes or no or I do not know for each topic.)*

	Yes	No	I do not know
A. Physical activity and fitness	O	O	O
B. Nutrition and dietary behaviour	O	O	O
C. **Sexual and reproductive health**[33]	O	O	O
D. HIV transmission, prevention, and treatment	O	O	O
E. Road safety	O	O	O
F. Violence and bullying prevention	O	O	O
G. Suicide prevention	O	O	O
H. Emotional and mental health	O	O	O
I. Tobacco and nicotine use prevention	O	O	O
J. Alcohol use prevention	O	O	O
K. Illicit drug use prevention	O	O	O
L. Infectious disease (such as the cold, flu, or COVID-19) prevention	O	O	O
M. Non-communicable disease (such as diabetes, cancer, or obesity) prevention	O	O	O
N. Oral health and prevention of oral disease	O	O	O
O. Healthy sleep	O	O	O
P. Personal hygiene, including handwashing	O	O	O
Q. Environmental health (such as the importance of clean air or water)	O	O	O
R. Sustainable development and consumption	O	O	O
S. Natural disaster preparedness and response	O	O	O
T. First aid	O	O	O
U. Immunizations (such as the HPV or COVID-19 vaccinations)	O	O	O
V. Safe sun exposure	O	O	O
W. Equity, inclusion, diversity, and human rights	O	O	O
X. Gender inequality and social norms	O	O	O

iew only

[33] Includes instruction on human growth and development, family life, reproduction, condoms and contraception, pregnancy, sexual behaviour, sexual abuse, and transmission and prevention of sexually transmitted infections.

For peer review only - http://bmjopen.bmj.com/site/about/guidelines.xhtml

6. Physical Education

Note: Ask this question if only males or only females attend this school.
6-01. Does this school teach **physical education**[34]?

A. Yes
B. No **(Go to 6-16)**
C. I do not know **(Go to 6-16)**

Note: Ask this question if both males and females attend this school.
6-02. Who is taught **physical education**[34] in this school?

A. All genders
B. Only males
C. Only females
D. No one **(Go to 6-16)**
E. I do not know **(Go to 6-16)**

6-03. Does this school provide adapted physical education for students with disabilities?

A. Yes
B. No
C. I do not know

6-04. Do students receive a grade for physical education?

A. Yes
B. No
C. I do not know

6-05. Can students in this school be excused from physical education for an extended period for the following reasons? *(Mark yes or no or I do not know for each reason.)*

	Yes	No	I do not know
A. Cultural	O	O	O
B. Gender	O	O	O
C. Academic achievement	O	O	O
D. Academic struggles or failure	O	O	O
E. Participation in other school activities	O	O	O
F. Poor physical health	O	O	O
G. Menstruation	O	O	O

Note: Ask G only if females attend this school.

6-06. Who provides **most** of the physical education instruction in this school?

A. A physical education teacher or specialist
B. A nurse or some other kind of health worker
C. Someone else
D. I do not know

[34] Class time spent teaching a physical education curriculum. Does not include instruction on physical activity topics in health education or any other subject.

For peer review only - http://bmjopen.bmj.com/site/about/guidelines.xhtml

6-07. Are the following materials provided to teachers in this school to guide physical education? *(Mark yes or no or I do not know for each type of material.)*

	Yes	No	I do not know
A. Learning outcomes or objectives	O	O	O
B. A planned progression of lesson plans or learning strategies and experiences	O	O	O
C. Teaching and learning resources and content	O	O	O
D. Assessment tools	O	O	O

6-08. Which of the following statements **best** describes how school policy addresses physical education for students?

A. School policy provides a specific plan or guidelines for implementing physical education
B. Physical education is mentioned in a school policy, but there is no specific plan or guideline for implementing physical education
C. Physical education is not addressed in any school policy
D. I do not know

6-09. Does this school routinely provide professional development opportunities for teachers to improve the quality of physical education instruction?

A. Yes
B. No
C. I do not know

Content of physical education

6-10. Are the following topics taught to students in this school? *(Mark yes or no or I do not know for each topic.)*

	Yes	No	I do not know
A. Movement concepts and skills (including motor skills such as walking or skipping and manipulative skills such as throwing, catching, or kicking)	O	O	O
B. The importance of life-long participation in physical activity	O	O	O
C. Developing an individualized physical activity plan, including frequency, intensity, and duration	O	O	O
D. The value and importance of fair play	O	O	O
E. Preventing injury during physical activity	O	O	O
F. The value of physical activity for health, enjoyment, challenge, self-expression, and/or social interaction	O	O	O
G. Strength training	O	O	O
H. Group or team sports or activities	O	O	O
I. Individual or paired sports or activities	O	O	O
J. The importance of endurance (aerobic) exercise for overall health and fitness	O	O	O
K. Flexibility or range of movement	O	O	O

Facilities and equipment

6-11. Does this school have a safe and clean **indoor** space for physical education class?

A. Yes
B. No
C. I do not know

For peer review only - http://bmjopen.bmj.com/site/about/guidelines.xhtml

6-12. Does this school have a safe and clean **outdoor** space for physical education class?

A. Yes
B. No **(Go to 6-16)**
C. I do not know **(Go to 6-16)**

6-13. Does this school allow community members to access this outdoor space for physical activity when school is not in session?

A. Yes
B. No
C. I do not know

6-14. Does this school provide a place where males and females can separately and privately change clothes before and after physical education?

A. Yes
B. No
C. I do not know

Note: Ask this question if both males and females attend this school.

6-15. Does this school have equipment (such as skipping ropes or balls) for use during physical education?

A. Yes
B. No
C. I do not know

Physical activity or recreation clubs and competitive sports

6-16. Does this school regularly provide recess or other physical activity breaks to students during the school day?

A. Yes
B. No
C. I do not know

6-17. Does this school offer **non-competitive physical activity or recreation clubs**[35] for students?

A. Yes
B. No
C. I do not know

6-18. Does this school offer school-sponsored sports teams that compete against teams from other schools?

A. Yes, for males only
B. Yes, for females only
C. Yes, for all genders
D. No
E. I do not know

Note: Ask this question if both males and females attend this school.

6-19. Does this school offer school-sponsored sports teams that compete against teams from other schools?

A. Yes
B. No
C. I do not know

Note: Ask this question if only males or only females attend this school.

[35] Any non-competitive physical activity program that is voluntary for students, in which students are given an equal opportunity to participate regardless of physical ability, and in which students have the opportunity to be involved in the planning, organization, and administration of the program, under the supervision of a qualified adult.

For peer review only - http://bmjopen.bmj.com/site/about/guidelines.xhtml

7. School Governance and Leadership

School health councils, committees, or teams

7-01. Does this school have an official council, committee, or team responsible for implementing health promoting school policies, programs, and activities?

A. Yes
B. No **(Go to 7-05)**
C. I do not know **(Go to 7-05)**

7-02. How often each year does this council, committee, or team routinely meet to address health promoting school policies, programs, and activities?

A. Weekly or more often
B. Monthly
C. Quarterly
D. Twice a year
E. Once a year or less often
F. I do not know

7-03. Are the following groups formally represented on this council, committee, or team? *(Mark yes or no or I do not know for each group.)*

	Yes	No	I do not know
A. School administrators	O	O	O
B. Teachers	O	O	O
C. Other school staff	O	O	O
D. Students	O	O	O
E. Parents, caregivers, or families	O	O	O
F. Government officials	O	O	O
G. Local businesses	O	O	O
H. Health workers (such as doctors or nurses)	O	O	O
I. Religious leaders	O	O	O

7-04. Does this council, committee, or team routinely conduct the following activities? *(Mark yes or no or I do not know for each activity.)*

	Yes	No	I do not know
A. Identify student health needs based on a review of relevant data	O	O	O
B. Recommend new or revised health promoting policies, programs, or activities to school administrators	O	O	O
C. Seek funding or leverage resources to support health promoting policies, programs, or activities for students or school staff	O	O	O
D. Communicate the importance of health promoting policies, programs, or activities to the school or community	O	O	O
E. Review health-related curricula or instructional materials	O	O	O

For peer review only - http://bmjopen.bmj.com/site/about/guidelines.xhtml

Community partnerships

7-05. Are the following groups regularly involved and engaged in **developing** health promoting school policies? *(Mark yes or no or I do not know for each group.)*

	Yes	No	I do not know
A. School administrators	O	O	O
B. Teachers	O	O	O
C. Other school staff	O	O	O
D. Students	O	O	O
E. Parents, caregivers, or families	O	O	O
F. Government officials	O	O	O
G. Local businesses	O	O	O
H. Health workers (such as doctors or nurses)	O	O	O
I. Religious leaders	O	O	O

7-06. Do the following groups regularly **receive copies of or information about** updated or new health promoting school policies? *(Mark yes or no or I do not know for each group.)*

	Yes	No	I do not know
A. School administrators	O	O	O
B. Teachers	O	O	O
C. Other school staff	O	O	O
D. Students	O	O	O
E. Parents, caregivers, or families	O	O	O
F. Government officials	O	O	O
G. Local businesses	O	O	O
H. Health workers (such as doctors or nurses)	O	O	O
I. Religious leaders	O	O	O

For peer review only - http://bmjopen.bmj.com/site/about/guidelines.xhtml

8. School Policies and Resources

8-01. Which of the following statements **best** describes how overall student health is promoted in school policy?

A. School policy provides a specific plan or guidance for promoting overall student health
B. Overall student health is mentioned in school policy, but without a specific plan or guidance on how to promote it
C. Overall student health is not addressed in any school policy
D. I do not know

8-02. Which of the following statements **best** describes the availability of resources in this school's budget to improve overall student health?

A. The school budget contains adequate resources to improve overall student health
B. The school budget contains some resources for improvement of overall student health, but not enough to do what is needed
C. The school budget does not contain any resources for improvement of overall student health
D. I do not know

8-03. Which of the following statements **best** describes how the health of teachers and other school staff is promoted in school policy?

A. School policy provides a specific plan or guidance for promoting the health of teachers and other school staff
B. The health of teachers and other school staff is mentioned in school policy, but without a specific plan or guidance on how to promote it
C. The health of teachers and other school staff is not addressed in any school policy
D. I do not know

8-04. Does this school regularly monitor, evaluate, or assess the quality of its health promoting policies, programs, or activities?

A. Yes
B. No
C. I do not know

The concept of a Health Promoting School embodies a whole-school approach to promoting health and educational attainment by capitalizing on the organizational potential of schools to foster the physical, social–emotional, and psychological conditions for health and positive educational outcomes. A Health Promoting School constantly strengthens its capacity as a safe and healthy setting for living, learning, and working. Other terms used to describe Health Promoting Schools are "comprehensive school health," "healthy school communities," and "school health education."

8-05. Does this school have a policy specifically about becoming or continuing to be a Health Promoting School?

A. Yes
B. No
C. I do not know

Overall curriculum

8-06. Does this school support **skills-based, participatory teaching methods**[36]?

A. Yes
B. No
C. I do not know

[36] Skills-based, participatory teaching methods are strategies and techniques focused on skill development that encourage students to become actively involved in their learning process.

For peer review only - http://bmjopen.bmj.com/site/about/guidelines.xhtml

8-07. Does this school allow teachers to make curriculum adaptations as appropriate to address the learning needs of students with disabilities?

A. Yes
B. No
C. I do not know

Professional development for teachers

8-08. Does this school routinely provide professional development opportunities for teachers on the following topics? *(Mark yes or no or I do not know for each topic.)*

	Yes	No	I do not know
A. The link between health and learning	O	O	O
B. Skills-based, participatory teaching methods	O	O	O
C. **Positive classroom management techniques**[37]	O	O	O
D. How to be a health promoting school	O	O	O
E. How to assess the health-related needs of students	O	O	O
F. How to deliver health-related content that best meets the needs of students	O	O	O
G. Standards for a healthy and safe learning environment	O	O	O
H. Child and adolescent development	O	O	O

Bullying and violence prevention

8-09. Does this school have a policy specifically prohibiting the following types of violence? *(Mark yes or no or I do not know for each type of violence.)*

	Yes	No	I do not know
A. Bullying among students	O	O	O
B. Fighting among students	O	O	O
C. Corporal punishment of students by teachers or other school staff	O	O	O
D. Physical, emotional, or sexual abuse of students by teachers or other school staff	O	O	O

8-10. Does this school have specific procedures in place for how a student can safely report any type of violence (such as bullying; fighting; corporal punishment; or physical, emotional, or sexual abuse)?

A. Yes
B. No
C. I do not know

8-11. Does this school have a policy describing how to respond when any type of violence (such as bullying; fighting; corporal punishment; or physical, emotional, or sexual abuse) occurs?

A. Yes
B. No
C. I do not know

[37] Positive classroom management techniques are focused on supporting and involving students to help them learn rather than focusing on bad behaviour.

For peer review only - http://bmjopen.bmj.com/site/about/guidelines.xhtml

8-12. Does this school collect data about incidences of violence (such as bullying; fighting; corporal punishment; or physical, emotional, or sexual abuse) including when, where, or how it occurred or who was involved?

A. Yes
B. No
C. I do not know

Tobacco use prevention

8-13. Does this school have a policy prohibiting use of at least some tobacco or nicotine products (such as cigarettes, other forms of smoked tobacco products, smokeless tobacco products, or electronic cigarettes) among the following groups on school premises? *(Mark yes or no or I do not know for each group.)*

	Yes	No	I do not know
A. Students	O	O	O
B. Teachers and other school staff	O	O	O
C. Visitors to the school	O	O	O

8-14. Does this school take the following actions to help reduce use of tobacco or nicotine products among students? *(Mark yes or no or I do not know for each action.)*

	Yes	No	I do not know
A. Implement policies on how to respond when students are caught using a tobacco or nicotine product on school premises	O	O	O
B. Post signs marking a tobacco-free school zone, that is, a specified distance from school grounds where tobacco use is prohibited	O	O	O
C. Prohibit advertising and promotion for tobacco and nicotine products on school premises	O	O	O

Alcohol use prevention

8-15. Does this school have a policy prohibiting use of alcohol among the following groups on school premises? *(Mark yes or no or I do not know for each group.)*

	Yes	No	I do not know
A. Students	O	O	O
B. Teachers and other school staff	O	O	O
C. Visitors to the school	O	O	O

8-16. Does this school take the following actions to help reduce use of alcohol among students? *(Mark yes or no or I do not know for each action.)*

	Yes	No	I do not know
A. Implement policies on how to respond when students are caught using alcohol on school premises	O	O	O
B. Post signs marking an alcohol-free school zone, that is, a specified distance from school grounds where alcohol use is prohibited	O	O	O
C. Prohibit advertising and promotion for alcohol products on school premises	O	O	O

For peer review only - http://bmjopen.bmj.com/site/about/guidelines.xhtml

Illicit drug use prevention

8-17. Does this school have a policy prohibiting use of illicit drugs among the following groups on school premises? *(Mark yes or no or I do not know for each group.)*

	Yes	No	I do not know
A. Students	O	O	O
B. Teachers and other school staff	O	O	O
C. Visitors to the school	O	O	O

8-18. Does this school take the following actions to help reduce use of illicit drugs among students? *(Mark yes or no or I do not know for each action.)*

	Yes	No	I do not know
A. Implement policies on how to respond when students are caught using illicit drugs on school premises	O	O	O
B. Post signs marking an illicit drug-free school zone, that is, a specified distance from school grounds where illicit drug use is prohibited	O	O	O

Crisis preparedness and emergency response

8-19. Does this school have a policy on crisis preparedness, response, and recovery from a natural disaster, conflict, pandemic, or other emergency situation?

A. Yes
B. No **(Go to 8-22)**
C. I do not know **(Go to 8-22)**

8-20. Does this policy ensure continuity for the following school services if the school building needs to be closed to students? *(Mark yes or no or I do not know for each service.)*

	Yes	No	I do not know
A. School health services	O	O	O
B. School food and nutrition services	O	O	O

8-21. Does this policy ensure continuity of instruction (possibly via remote learning) for the following subjects (or curricula) if the school building needs to be closed to students? *(Mark yes or no or I do not know for each subject.)*

	Yes	No	I do not know
A. Health education	O	O	O
B. Physical education	O	O	O
C. Other subjects	O	O	O

8-22. Does this school conduct regular emergency drills, such as fire or earthquake drills?

A. Yes
B. No
C. I do not know

8-23. Is this school officially designated to serve as a shelter for community members during or after a natural disaster?

A. Yes
B. No
C. I do not know

For peer review only - http://bmjopen.bmj.com/site/about/guidelines.xhtml

8-24. Does this school have measures in place in case of violence or conflict to help protect students and school staff (such as locked entry points, outsider identification processes, metal detectors, or security guards)?

A. Yes
B. No
C. I do not know

Eye health

8-25. Does this school have a policy requiring a minimum amount of outdoor activity daily to help prevent myopia or nearsightedness?

A. Yes
B. No
C. I do not know

Pregnant students

8-26. Does this school have a policy to allow pregnant students to attend school either in person or via remote learning?

A. Yes
B. No
C. I do not know

Note: Ask this question only if females attend this school.

For peer review only - http://bmjopen.bmj.com/site/about/guidelines.xhtml

GLOBAL SCHOOL-BASED STUDENT HEALTH SURVEY
2021 Version

This survey is about your health and the things you do that may affect your health. Students like you all over your country are doing this survey. Students in many other countries around the world also are doing this survey. The information you give will be used to develop better health programs for young people like yourself.

DO NOT write your name on this survey or the answer sheet. The answers you give will be kept private. No one will know how you answer. Answer the questions based on what you really know or do. There are no right or wrong answers.

Completing the survey is voluntary. Your grade or mark in this class will not be affected whether or not you answer the questions. If you do not want to answer a question, just leave it blank.

Make sure to read every question. Fill in the circles on your answer sheet that match your answer. Use only the pencil you are given. When you are done, do what the person who is giving you the survey says to do.

Here is an example of how to fill in the circles:

Fill in the circles like this Not like this or

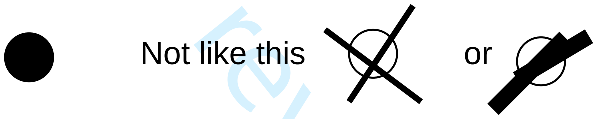

Survey

1. Do fish live in water?
   A. Yes
   B. No

Answer sheet

1. ● Ⓑ Ⓒ Ⓓ Ⓔ Ⓕ Ⓖ Ⓗ

Thank you very much for your help.

For peer review only

GSHS Core Questionnaire Respondent Demographics Module

1. How old are you?

   A. 11 years old or younger
   B. 12 years old
   C. 13 years old
   D. 14 years old
   E. 15 years old
   F. 16 years old
   G. 17 years old
   H. 18 years old or older

2. What is your sex?

   A. Male
   B. Female

3. In what grade/class/standard are you?
   COUNTRY SPECIFIC RESPONSE OPTIONS

   A. OPTION 1
   B. OPTION 2
   C. OPTION 3
   D. OPTION 4
   E. OPTION 5
   F. OPTION 6

For peer review only

For peer review only - http://bmjopen.bmj.com/site/about/guidelines.xhtml

GSHS Core Questionnaire Alcohol Use Module

The next 8 questions ask about drinking alcohol. This includes drinking COUNTRY SPECIFIC EXAMPLES. Drinking alcohol does not include drinking a few sips of wine for religious purposes. A "drink" is a glass of wine, a bottle of beer, a small glass of liquor or home brew, or a mixed drink.

1. How old were you when you had your first drink of alcohol other than a few sips?

   A. I have never had a drink of alcohol other than a few sips
   B. 7 years old or younger
   C. 8 or 9 years old
   D. 10 or 11 years old
   E. 12 or 13 years old
   F. 14 or 15 years old
   G. 16 or 17 years old
   H. 18 years old or older

2. During the past 30 days, on how many days did you have at least one drink containing alcohol?

   A. 0 days
   B. 1 or 2 days
   C. 3 to 5 days
   D. 6 to 9 days
   E. 10 to 19 days
   F. 20 to 29 days
   G. All 30 days

3. During the past 30 days, on the days you drank alcohol, how many drinks did you **usually** drink <u>per day</u>?

   A. I did not drink alcohol during the past 30 days
   B. Less than one drink
   C. 1 drink
   D. 2 drinks
   E. 3 drinks
   F. 4 drinks
   G. 5 or more drinks

4. During the past 30 days, what is the largest number of alcoholic drinks you had in a row,that is, within a couple of hours?

   A. I did not drink alcohol during the past 30 days
   B. 1 or 2 drinks
   C. 3 drinks
   D. 4 drinks
   E. 5 drinks
   F. 6 or 7 drinks
   G. 8 or 9 drinks
   H. 10 or more drinks

5. During the past 30 days, how did you **usually** get the alcohol you drank? SELECT ONLY ONE RESPONSE.

   A. I did not drink alcohol during the past 30 days
   B. I bought it in a store, shop, or from a street vendor
   C. I gave someone else money to buy it for me
   D. I got it from my friends
   E. I got it from my family
   F. I stole it or got it without permission
   G. I got it some other way

6. During your life, how many times have you got into trouble at home, work, or school or got into fights, as a result of drinking alcohol?

   A. 0 times
   B. 1 or 2 times
   C. 3 to 5 times
   D. 6 to 9 times
   E. 10 to 19 times
   F. 20 or more times

Staggering when walking, not being able to speak right, throwing up, and passing out are some signs of being really drunk.

7. During your life, how many times have you drank so much alcohol that you were really drunk?

   A. 0 times
   B. 1 or 2 times
   C. 3 to 5 times
   D. 6 to 9 times
   E. 10 to 19 times
   F. 20 or more times

For peer review only - http://bmjopen.bmj.com/site/about/guidelines.xhtml

GSHS Supplementary question on school attendance under the influence of alcohol

8. During the past 12 months, how many times did you attend school under the influence of alcohol?

   A. 0 times
   B. 1 or 2 times
   C. 3 to 5 times
   D. 6 to 9 times
   E. 10 to 19 times
   F. 20 to 39 times
   G. 40 or more times

For peer review only

For peer review only - http://bmjopen.bmj.com/site/about/guidelines.xhtml

GSHS Core Questionnaire Dietary Behaviours Module

The next 3 questions ask about your height, weight, and going hungry.

1. How tall are you without your shoes on? ON THE ANSWER SHEET, WRITE YOUR HEIGHT IN THE SHADED BOXES AT THE TOP OF THE GRID. THEN FILL IN THE CIRCLE BELOW EACH NUMBER.

Example

Height (cm)		
1	***5***	***3***
0	0	0
●	1	1
2	2	2
	3	●
	4	4
	●	5
	6	6
	7	7
	8	8
	9	9
	I do not know 9	

2. How much do you weigh without your shoes on? ON THE ANSWER SHEET, WRITE YOUR WEIGHT IN THE SHADED BOXES AT THE TOP OF THE GRID. THEN FILL IN THE CIRCLE BELOW EACH NUMBER.

Example

Weight (kg)		
0	***5***	***2***
●	0	0
1	1	1
2	2	●
	3	3
	4	4
	●	5
	6	6
	7	7
	8	8
	9	9
	I do not know 9	

3. During the past 30 days, how often did you go hungry because there was not enough food in your home?

   A. Never
   B. Rarely
   C. Sometimes
   D. Most of the time
   E. Always

The next 4 questions ask about what you might eat and drink.

4. During the past 7 days, how many times did you eat fruit, such as COUNTRY SPECIFIC EXAMPLES?

   A. I did not eat fruit during the past 7 days
   B. 1 to 3 times during the past 7 days
   C. 4 to 6 times during the past 7 days
   D. 1 time per day
   E. 2 times per day
   F. 3 times per day
   G. 4 or more times per day

5. During the past 7 days, how many times did you eat vegetables, such as COUNTRY SPECIFIC EXAMPLES?

   A. I did not eat vegetables during the past 7 days
   B. 1 to 3 times during the past 7 days
   C. 4 to 6 times during the past 7 days
   D. 1 time per day
   E. 2 times per day
   F. 3 times per day
   G. 4 or more times per day

6. During the past 7 days, how many times did you drink a can, bottle, or glass of a carbonated soft drink, such as COUNTRY SPECIFIC EXAMPLES? (Do **not** count diet soft drinks.)

   A. I did not drink carbonated soft drinks during the past 7 days
   B. 1 to 3 times during the past 7 days
   C. 4 to 6 times during the past 7 days
   D. 1 time per day
   E. 2 times per day
   F. 3 times per day
   G. 4 or more times per day

For peer review only

GSHS Core Questionnaire Dietary Behaviours Module

For this question, sugar-sweetened drinks include sports drinks (COUNTRY SPECIFIC EXAMPLES), energy drinks (COUNTRY SPECIFIC EXAMPLES), 100% fruit juices (COUNTRY SPECIFIC EXAMPLES), fruit drinks that are not 100% juice (COUNTRY SPECIFIC EXAMPLES), sugar-sweetened flavoured milks (COUNTRY SPECIFIC EXAMPLES), and sugar-sweetened teas, coffees, or flavoured waters.

For this question, DO NOT COUNT carbonated soft drinks measured in the previous question or diet or no calorie drinks.

7. During the past 7 days, how many times did you drink a can, bottle, or glass of a sugar-sweetened drink?

   A. I did not drink sugar-sweetened drinks during the past 7 days
   B. 1 to 3 times during the past 7 days
   C. 4 to 6 times during the past 7 days
   D. 1 time per day
   E. 2 times per day
   F. 3 times per day
   G. 4 or more times per day

For peer review only

For peer review only - http://bmjopen.bmj.com/site/about/guidelines.xhtml

GSHS Core Questionnaire Drug Use Module and supplementary question on school attendance under the influence of drugs

The next 4 questions ask about drug use. This includes using cannabis (also called marijuana), amphetamines, cocaine, inhalants, and heroin, and COUNTRY SPECIFIC EXAMPLES. Do not count tobacco and alcohol use.

1. How old were you when you first used drugs?

   A. I have never used drugs
   B. 7 years old or younger
   C. 8 or 9 years old
   D. 10 or 11 years old
   E. 12 or 13 years old
   F. 14 or 15 years old
   G. 16 or 17 years old
   H. 18 years old or older

2. During your life, how many times have you used cannabis (also called marijuana and COUNTRY SPECIFIC SLANG TERMS FOR CANNABIS)?

   A. 0 times
   B. 1 or 2 times
   C. 3 to 5 times
   D. 6 to 9 times
   E. 10 to 19 times
   F. 20 or more times

3. During the past 30 days, how many times did you use cannabis (also called marijuana and COUNTRY SPECIFIC SLANG TERMS FOR CANNABIS)?

   A. 0 times
   B. 1 or 2 times
   C. 3 to 5 times
   D. 6 to 9 times
   E. 10 to 19 times
   F. 20 or more times

4. During your life, how many times have you used amphetamines or methamphetamines (also called COUNTRY SPECIFIC SLANG TERMS FOR AMPHETAMINES AND METHAMPHETAMINES) for non-medical purposes?

   A. 0 times
   B. 1 or 2 times
   C. 3 to 5 times
   D. 6 to 9 times
   E. 10 to 19 times
   F. 20 or more times

5. During the past 12 months, how many times did you attend school under the influence of drugs, such as cannabis or cocaine?

   A. 0 times
   B. 1 or 2 times
   C. 3 to 5 times
   D. 6 to 9 times
   E. 10 to 19 times
   F. 20 or more times

For peer review only

GSHS Core Questionnaire Hygiene Module

The next 3 questions ask about your oral health.

1. During the past 30 days, how many times per day did you **usually** clean or brush your teeth?

   A. I did not clean or brush my teeth during the past 30 days
   B. Less than 1 time per day
   C. 1 time per day
   D. 2 times per day
   E. 3 or more times per day

2. During the past 30 days, did you **usually** use a toothpaste that contains fluoride when you cleaned or brushed your teeth?

   A. I did not clean or brush my teeth during the past 30 days
   B. Yes, I usually used a toothpaste that contains fluoride
   C. No, I did not usually use a toothpaste that contains fluoride
   D. I do not know if the toothpaste I usually used contains fluoride

3. During the past 30 days, did a problem with your mouth, teeth, or gums cause you to miss classes or school?

   A. Yes
   B. No

The next 3 questions ask about washing your hands.

4. During the past 30 days, how often did you wash your hands before eating?

   A. Never
   B. Rarely
   C. Sometimes
   D. Most of the time
   E. Always

5. During the past 30 days, how often did you wash your hands after using the toilet or latrine?

   A. Never
   B. Rarely
   C. Sometimes
   D. Most of the time
   E. Always

6. During the past 30 days, how often did you use soap when washing your hands?

   A. Never
   B. Rarely
   C. Sometimes
   D. Most of the time
   E. Always

For peer review only

GSHS Core Questionnaire Mental Health Module

The next 3 questions ask about your friendships and feelings.

1. How many close friends do you have?

   A. 0 friends
   B. 1 friend
   C. 2 friends
   D. 3 or more friends

2. During the past 12 months, how often did you feel lonely?

   A. Never
   B. Rarely
   C. Sometimes
   D. Most of the time
   E. Always

3. During the past 12 months, how often were you so worried about something that you could not sleep at night?

   A. Never
   B. Rarely
   C. Sometimes
   D. Most of the time
   E. Always

Sometimes people feel so depressed about the future that they may consider attempting suicide, that is, taking some action to end their own life. The next 3 questions ask about attempted suicide.

4. During the past 12 months, did you **seriously** consider attempting suicide?

   A. Yes
   B. No

5. During the past 12 months, did you make a plan about how you would attempt suicide?

   A. Yes
   B. No

6. During the past 12 months, how many times did you attempt suicide?

   A. 0 times
   B. 1 time
   C. 2 or 3 times
   D. 4 or 5 times
   E. 6 or more times

For peer review only

For peer review only - http://bmjopen.bmj.com/site/about/guidelines.xhtml

GSHS Core Questionnaire Physical Activity Module

The next 4 questions ask about physical activity. Physical activity is any activity that increases your heart rate and makes you breathe hard. Physical activity can be done in sports, playing with friends, walking to school, or in physical education class. Some examples of physical activity are running, fast walking, biking, dancing, football, and COUNTRY SPECIFIC EXAMPLES.

1. During the past 7 days, on how many days were you physically active for a total of at least 60 minutes per day? ADD UP ALL THE TIME YOU SPENT IN ANY KIND OF PHYSICAL ACTIVITY EACH DAY.

   A. 0 days
   B. 1 day
   C. 2 days
   D. 3 days
   E. 4 days
   F. 5 days
   G. 6 days
   H. 7 days

2. During the past 7 days, on how many days did you do exercises to strengthen or tone your muscles, such as push-ups, sit-ups, or weightlifting?

   A. 0 days
   B. 1 day
   C. 2 days
   D. 3 days
   E. 4 days
   F. 5 days
   G. 6 days
   H. 7 days

3. During the past 7 days, on how many days did you walk or ride a bicycle to or from school?

   A. 0 days
   B. 1 day
   C. 2 days
   D. 3 days
   E. 4 days
   F. 5 days
   G. 6 days
   H. 7 days

4. During this school year, on how many days did you go to physical education (PE) class each week?

   A. 0 days
   B. 1 day
   C. 2 days
   D. 3 days
   E. 4 days
   F. 5 or more days

The next question asks about the time you spend mostly sitting or lying down when you are not in school or doing homework.

5. How much time do you spend during a **typical or usual** day sitting or lying down doing such things as watching television, playing computer games, talking with friends, using your mobile phone, traveling in a motor vehicle, napping, or doing other activities sitting or lying down, such as COUNTRY SPECIFIC EXAMPLES? (Do **not** count time spent sleeping at night.)

   A. Less than 1 hour per day
   B. 1 to 2 hours per day
   C. 3 to 4 hours per day
   D. 5 to 6 hours per day
   E. 7 to 8 hours per day
   F. More than 8 hours per day

The next question asks about how much sleep you get.

6. On an average school night, how many hours of sleep do you get?

   A. 4 or less hours
   B. 5 hours
   C. 6 hours
   D. 7 hours
   E. 8 hours
   F. 9 hours
   G. 10 hours
   H. 11 or more hours

For peer review only - http://bmjopen.bmj.com/site/about/guidelines.xhtml

GSHS Core Questionnaire Protective Factors Module

The next 7 questions ask about your experiences at school and at home.

1. During the past 30 days, on how many days did you miss classes or school without permission?

   A. 0 days
   B. 1 or 2 days
   C. 3 to 5 days
   D. 6 to 9 days
   E. 10 or more days

2. During the past 30 days, how often were most of the students in your school kind and helpful?

   A. Never
   B. Rarely
   C. Sometimes
   D. Most of the time
   E. Always

3. During the past 30 days, how often were you able to talk to someone about difficult problems and worries?

   A. Never
   B. Rarely
   C. Sometimes
   D. Most of the time
   E. Always

4. During the past 30 days, how often did your parents or guardians understand your problems and worries?

   A. Never
   B. Rarely
   C. Sometimes
   D. Most of the time
   E. Always

5. During the past 30 days, how often did your parents or guardians check to see if your homework was done?

   A. Never
   B. Rarely
   C. Sometimes
   D. Most of the time
   E. Always

6. During the past 30 days, how often did your parents or guardians **really** know what you were doing with your free time?

   A. Never
   B. Rarely
   C. Sometimes
   D. Most of the time
   E. Always

7. During the past 30 days, how often did your parents or guardians go through your things without your approval?

   A. Never
   B. Rarely
   C. Sometimes
   D. Most of the time
   E. Always

For peer review only

For peer review only - http://bmjopen.bmj.com/site/about/guidelines.xhtml

GSHS Core Questionnaire Sexual Behaviours That Contribute to HIV Infection, Other STI, and Unintended Pregnancy Module

The next 5 questions ask about sexual intercourse.

1. Have you ever had sexual intercourse?

   A. Yes
   B. No

2. How old were you when you had sexual intercourse for the first time?

   A. I have never had sexual intercourse
   B. 11 years old or younger
   C. 12 years old
   D. 13 years old
   E. 14 years old
   F. 15 years old
   G. 16 or 17 years old
   H. 18 years old or older

3. During your life, with how many people have you had sexual intercourse?

   A. I have never had sexual intercourse
   B. 1 person
   C. 2 people
   D. 3 people
   E. 4 people
   F. 5 people
   G. 6 or more people

4. The **last time** you had sexual intercourse, did you or your partner use a condom or [COUNTRY SPECIFIC SLANG TERM FOR CONDOM]?

   A. I have never had sexual intercourse
   B. Yes
   C. No

5. The **last time** you had sexual intercourse, what **one** method did you or your partner use to **prevent pregnancy**? SELECT ONLY ONE RESPONSE.

   A. I have never had sexual intercourse
   B. No method was used to prevent pregnancy
   C. Birth control pills
   D. Condoms
   E. An IUD or implant
   F. A shot, patch, or birth control ring
   G. Withdrawal or some other method including COUNTRY SPECIFIC METHOD
   H. I do not know

For peer review only - http://bmjopen.bmj.com/site/about/guidelines.xhtml

For peer review only

GSHS Core Questionnaire Tobacco Use Module

The next 3 questions ask about cigarette use. Cigarettes include manufactured cigarettes, roll-your-own cigarettes, and kretek cigarettes, and COUNTRY SPECIFIC EXAMPLES.

1. Have you ever tried or experimented with cigarette smoking, even one or two puffs?

   A. Yes
   B. No

2. How old were you when you first tried smoking a cigarette?

   A. I have never tried smoking a cigarette
   B. 7 years old or younger
   C. 8 or 9 years old
   D. 10 or 11 years old
   E. 12 or 13 years old
   F. 14 or 15 years old
   G. 16 or 17 years old
   H. 18 years old or older

3. During the past 30 days, on how many days did you smoke cigarettes?

   A. 0 days
   B. 1 or 2 days
   C 3 to 5 days
   D. 6 to 9 days
   E. 10 to 19 days
   F. 20 to 29 days
   G. All 30 days

The next question asks about other forms of smoked tobacco products other than cigarettes. This includes pipes, cigars, mini cigars, cigarillos, waterpipes, hookah, shisha, narghile, hubble-bubble, bidis, and heated tobacco products (HTPs) and COUNTRY SPECIFIC EXAMPLES.

4. During the past 30 days, on how many days did you use any form of smoked tobacco products other than cigarettes?

   A. 0 days
   B. 1 or 2 days
   C 3 to 5 days
   D. 6 to 9 days
   E. 10 to 19 days
   F. 20 to 29 days
   G. All 30 days

The next question asks about smokeless tobacco products. This includes snuff, chewing tobacco, dip, betel quid with tobacco, and gutka and COUNTRY SPECIFIC EXAMPLES.

5. During the past 30 days, on how many days did you use any form of smokeless tobacco products?

   A. 0 days
   B. 1 or 2 days
   C 3 to 5 days
   D. 6 to 9 days
   E. 10 to 19 days
   F. 20 to 29 days
   G. All 30 days

The next question asks about electronic cigarettes. Electronic cigarettes, or e-cigarettes, are electronic devices that usually contain a nicotine-based liquid that is vaporized and inhaled. You may also know them as vape-pens, hookah-pens, electronic hookahs (e-hookahs), electronic cigars (e-cigars), electronic pipes (e-pipes), or e-vaporizers. Some look like cigarettes and others look like pens or small pipes. They are battery-powered devices that produce vapor instead of smoke. They do not contain tobacco.

6. During the past 30 days, on how many days did you use electronic cigarettes?

   A. 0 days
   B. 1 or 2 days
   C 3 to 5 days
   D. 6 to 9 days
   E. 10 to 19 days
   F. 20 to 29 days
   G. All 30 days

For peer review only

For peer review only - http://bmjopen.bmj.com/site/about/guidelines.xhtml

GSHS Core Questionnaire Violence and Unintentional Injury Module

The next 3 questions ask about serious injuries that happened to you. An injury is serious when it makes you miss at least one full day of usual activities (such as school, sports, or a job) or requires treatment by a doctor or nurse.

1. During the past 12 months, how many **times** were you seriously injured?

   A. 0 times
   B. 1 time
   C. 2 or 3 times
   D. 4 or 5 times
   E. 6 or 7 times
   F. 8 or 9 times
   G. 10 or 11 times
   H. 12 or more times

2. During the past 12 months, what was the **most serious injury** that happened to you?

   A. I was not seriously injured during the past 12 months
   B. I had a broken bone, a dislocated joint, or a broken or knocked out tooth
   C. I had a cut or stab wound
   D. I had a concussion or other head or neck injury, was knocked out, or could not breathe
   E. I had a gunshot wound
   F. I had a bad burn
   G. I was poisoned or took too much of a drug
   H. Something else happened to me

3. During the past 12 months, **what was the major cause** of the most serious injury that happened to you?

   A. I was not seriously injured during the past 12 months
   B. I was in a motor vehicle accident or hit by a motor vehicle
   C. I fell
   D. Something fell on me or hit me
   E. I was attacked or abused or was fighting with someone
   F. I was in a fire or too near a flame or something hot
   G. I inhaled or swallowed something bad for me
   H. Something else caused my injury

The next question asks about physical attacks. A physical attack occurs when one or more people hit or strike someone, or when one or more people hurt another person with a weapon (such as a stick, knife, or gun). It is not a physical attack when two students of about the same strength or power choose to fight each other.

4. During the past 12 months, how many times were you physically attacked?

   A. 0 times
   B. 1 time
   C. 2 or 3 times
   D. 4 or 5 times
   E. 6 or 7 times
   F. 8 or 9 times
   G. 10 or 11 times
   H. 12 or more times

The next question asks about physical fights. A physical fight occurs when two students of about the same strength or power choose to fight each other.

5. During the past 12 months, how many times were you in a physical fight?

   A. 0 times
   B. 1 time
   C. 2 or 3 times
   D. 4 or 5 times
   E. 6 or 7 times
   F. 8 or 9 times
   G. 10 or 11 times
   H. 12 or more times

For peer review only - http://bmjopen.bmj.com/site/about/guidelines.xhtml

GSHS Core Questionnaire Violence and Unintentional Injury Module

The next 3 questions ask about bullying. Bullying occurs when one or more students or other people about your age say or do hurtful or mean things. Bullying can occur when someone teases, threatens, ignores, spreads rumors about, calls someone a bad name, makes sexual remarks, or hits, shoves, or hurts another person over and over again. It is not bullying when two people of about the same strength or power argue or fight or tease each other in a friendly way.

6. During the past 12 months, were you bullied **on school property**?

   A. Yes
   B. No

7. During the past 12 months, were you bullied when you were **not on school property**?

   A. Yes
   B. No

Cyber bullying is a form of bullying using social media and other forms of online communication. Cyber bullying may happen on Instagram, Twitter, Snapchat, and Facebook, COUNTRY SPECIFIC EXAMPLES, and other social media platforms or through texting and email.

8. During the past 12 months, were you **cyber** bullied?

   A. Yes
   B. No

For peer review only - http://bmjopen.bmj.com/site/about/guidelines.xhtml

GSHS Supplementary questions on Physical Activity and the COVID-19 pandemic

The next question asks about physical activity. It is similar to but different than a question you were asked earlier. As a reminder, physical activity is any activity that increases your heart rate and makes you breathe hard. Physical activity can be done in sports, playing with friends, walking to school, or in physical education class. Some examples of physical activity are running, fast walking, biking, dancing, football, badminton, tennis, kabaddi, kho-kho, cricket, and swimming.

1. During the past 7 days, did you do at least **an average of** 60 minutes per day of physical activity across the 7 days?

   A. Yes
   B. No

Beginning in late 2019, the world experienced the coronavirus disease (COVID-19) pandemic. As part of the response to this pandemic, some schools and businesses were closed and some people were required to stay at home. Your community might still be experiencing the pandemic. The next question ask about the COVID-19 pandemic.

1. During the COVID-19 pandemic, did you attend school from home at least some of the time using a computer, mobile phone, or other electronic device?

   A. Yes
   B. No

For peer review only - http://bmjopen.bmj.com/site/about/guidelines.xhtml

Global School-Based Student Health Survey (GSHS) Parental/Guardian Notification Form

[Date]

Introduction

[Name of school] is participating in a study to help empower students to lead change using health data. Three other cities around the world are also participating. The study is sponsored by [name of agency]. The study has two main components.

Global School-Based Student Health Survey (GSHS)

The GSHS is a paper-and-pencil survey being given to a small number of students aged 13-17 across our city. It asks students about health behaviours and experiences. Students will not get any immediate benefit from taking part in the GSHS. However, the results of this survey will be used by some students, teachers, and local leaders to help develop a program to reduce student risk behaviors. Questions will be asked about the following topics:
[list the selected core modules].

Timeframe

Students will be asked to fill out a paper questionnaire during regular class time and it will take about 30 to 45 minutes to complete.

Physical activity study

In addition to the GSHS, students from one class in each school will be asked to wear an activity monitor on their wrist for one week. The activity monitor will measure physical activity (sitting, standing, walking, running, and lying down). The activity monitor will not interfere with their daily activities. Students in activity monitor classrooms will be given detailed directions about how to wear the monitor. At the end of the week, they will be asked to complete the GSHS just like students in all other sampled classes.

Student privacy

Survey procedures have been designed to protect student privacy. Students do not put their name on the questionnaire or answer sheet. No school or student is ever mentioned by name in a report of the results. Some students may find some questions to be a little sensitive.

Voluntary participation

We would like all students to take part in the survey and physical activity study, **but both activities are voluntary**. No action will be taken against the school, you, or a student, if a student does not take part. Students can skip any question that they do not wish to answer, students may stop participating in the survey at any point without penalty, and students may refuse to wear the activity monitor.

For peer review only

**Questions**	If you have any questions, please contact [name and contact information of survey coordinator].<br>Thank you for your cooperation.

For peer review only

Global School-Based Student Health Survey (GSHS) Parental/Guardian Permission Form

**Introduction**	[Name of school] is participating in a study to help empower students to lead change using health data. Three other cities around the world are also participating. The study is sponsored by [name of agency]. The study has two main components.
**Global School-Based Student Health Survey (GSHS)**	The GSHS is a paper-and-pencil survey being given to a small number of students aged 13-17 across our city. It asks students about health behaviours and experiences. Students will not get any immediate benefit from taking part in the GSHS. However, the results of this survey will be used by some students, teachers, and local leaders to help develop a program to reduce student risk behaviors. Questions will be asked about the following topics: [list the selected core modules].
**Timeframe**	Students will be asked to fill out a paper questionnaire during regular class time and it will take about 30 to 45 minutes to complete.
**Physical activity study**	In addition to the GSHS, students from one class in each school will be asked to wear an activity monitor on their wrist for one week. The activity monitor will measure physical activity (sitting, standing, walking, running, and lying down). The activity monitor will not interfere with their daily activities. Students in activity monitor classrooms will be given detailed directions about how to wear the monitor. At the end of the week, they will be asked to complete the GSHS just like students in all other sampled classes.
**Student privacy**	Survey procedures have been designed to protect student privacy. Students do not put their name on the questionnaire or answer sheet. No school or student is ever mentioned by name in a report of the results. Some students may find some questions to be a little sensitive.
**Voluntary participation**	We would like all students to take part in the survey and physical activity study, **but both activities are voluntary**. No action will be taken against the school, you, or a student, if a student does not take part. Students can skip any question that they do not wish to answer, students may stop participating in the survey at any point without penalty, and students may refuse to wear the activity monitor.
**Questions**	If you have any questions, please contact [name and contact information of survey coordinator]. Thank you for your cooperation.

Please complete and return the following permission form by [date] if you do NOT want your student to take part in the study.

For peer review only

Student’s name:____

I have read this form and know what the survey is about.
[ ] My student may **NOT** take part in this survey.

Parent/guardian signature:______________________________________ Date:___________________

For peer review only

Student Assent Form

**Introduction**	Your school is part of a study to help empower students to lead change using health data. This study is being done in three other cities around the world.<br>The study has several parts:<br>• a survey (called the Global School-based Student Health Survey – GSHS) where you will be asked to answer questions about your health;<br>• a physical activity study where some students will be asked to wear an activity monitor on their wrist;<br>• a workshop where some students will be asked to help come up with ideas to improve the health of all students in your school.
**Global School-Based Student Health Survey (GSHS)**	This survey is about your health and the things you do that may affect your health. Students like you from __ schools all over your city are doing this survey. Students in many other countries around the world have also done this survey before. The information you give will be used to develop better health programs for young people like yourself.<br>You will be asked to complete a paper questionnaire during regular class time. It will take about 30 to 45 minutes.<br>The answers you give will be kept private. No one will know how you answer. Please answer the questions based on what you really know or do. There are no right or wrong answers.
**Physical activity study**	Students from some classes also will be asked to wear an activity monitor on their wrist for one week. The activity monitor will measure physical activity (sitting, standing, walking, running, and lying down). The activity monitor will not interfere with anything you do. You will be given detailed directions about how to wear the monitor. At the end of the week, you will give the activity monitor back and then complete the GSHS just like students in other classes. The information collected with the activity monitor will be kept private and your name will not be recorded in the activity monitor.
**Workshop**	Some students will be asked to take part in data-to-action workshops. In these workshops, students, teachers, and local leaders will look at the study data and develop ideas on how the health of all students in your school could be improved.
**Your privacy**	The entire study has been designed to protect your privacy. There will not be any student, class, or school names on any questionnaire or answer sheet or recorded in the activity monitor. No school or student will be mentioned by name in a report of the results.

For peer review only

**Voluntary participation**	We would like all selected students to take part in the study, **but your participation is voluntary**. Your grade or mark in this class will not be affected whether or not you answer the questions or wear the activity monitor. If you do not want to answer a question, just leave it blank, you may stop participating in the study at any point without penalty, you may refuse to wear the activity monitor, and you do not have to volunteer for the data-to-action workshops.
**Questions**	If you have any questions, please contact [name and contact information of survey coordinator]. Thank you.

Please complete and return the following form by [date] if you do NOT want to take part in the study.

Your name:____

I have read this form and know what the study is about.
[ ] I do **NOT** want to take part in this study.

Student signature:_______________________________________ Date:____________________

For peer review only

Data to action workshop to improve adolescent health Parental/Guardian Notification Form

**Introduction**	[Name of school] has recently done a Global School-based Student Health Survey (GSHS) and a Global School Health Policies and Practices Survey (G-SHPSS) sponsored by [name of agency]. The GSHS collects information on health behaviours in 13-17 year old students, and the G-SHPPS on existing practices in schools. In the data to action workshop, the collected information will be used to plan interventions to improve the health of adolescents.
**Global AA-HA! workshop**	The data to action workshop will follow the Global Accelerated Action for the Health of Adolescents (Global AA-HA!) approach to improve young people's health. During the workshop, students, teachers and local authorities will explore information on student's behaviours and school practices together, identify where action is most needed, and set priorities for interventions to improve adolescent health. Students will not get any immediate benefit from taking part in the workshop. However, the results of the workshop will help students and other adolescents in the future.
**Timeframe**	Participation in the workshop will take about two hours per day on three consecutive days.
**Student privacy**	Workshop procedures have been designed to protect student privacy. No school or student will ever be mentioned by name in workshop documents.
**Voluntary participation**	**Participation in the workshop is voluntary**. Only parents/guardians of students that have put their name forward for voluntary participation in the workshop receive this permission form. If students wish, they may stop participating in the workshop at any point without penalty.
**Questions**	If you have any questions, please contact [name of local contact]. Thank you for your cooperation.

For peer review only

Data to action workshop to improve adolescent health Parental/Guardian Permission Form

**Introduction**	[Name of school] has recently done a Global School-based Student Health Survey (GSHS) and a Global School Health Policies and Practices Survey (G-SHPSS) sponsored by [name of agency]. The GSHS collects information on health behaviours in 13-17 year old students, and the G-SHPPS on existing practices in schools. In the data to action workshop, the collected information will be used to plan interventions to improve the health of adolescents.
**Global AA-HA! workshop**	The data to action workshop will follow the Global Accelerated Action for the Health of Adolescents (Global AA-HA!) approach to improve young people's health. During the workshop, students, teachers and local authorities will explore information on student's behaviours and school practices together, identify where action is most needed, and set priorities for interventions to improve adolescent health. Students will not get any immediate benefit from taking part in the workshop. However, the results of the workshop will help students and other adolescents in the future.
**Timeframe**	Participation in the workshop will take about two hours per day on three consecutive days.
**Student privacy**	Workshop procedures have been designed to protect student privacy. No school or student will ever be mentioned by name in workshop documents.
**Voluntary participation**	**Participation in the workshop is voluntary**. Only parents/guardians of students that have put their name forward for voluntary participation in the workshop receive this permission form. If students wish, they may stop participating in the workshop at any point without penalty.
**Questions**	If you have any questions, please contact [name of local contact]. Thank you for your cooperation.

Please complete and return the following permission form by [date] if you do NOT want your student to take part in the workshop.

Student's name:____

I have read this form and know what the workshop is about.

[ ] My student may **NOT** take part in this workshop.

Parent/guardian signature:____ Date:_____________________

For peer review only

Empowering Adolescents to lead change using health data

Working Agenda
Data-to-Action Workshop

The aim of this project is to develop, document and support the implementation of an intervention approach to improve the health of students in four selected cities (Sekondi-Takoradi, Ghana; Jaipur, India; St Catherine Parish, Jamaica; Fez, Morocco) using locally collected data. This is done through two surveys: The health-related data collected by the cities high schools via the **Global Schoolbased Student Health Survey (GSHS)** and the **Global School Health Policies and Practices Study (G-SHPPS).** Additionally, these data are enhanced by **photovoice** taken by students, who represent approximately 15 schools per city.

This three-day interactive workshop serves the above aim. During the workshop and based on the findings of the above surveys and photovoice, the Global Accelerated Action for the Health of Adolescents (**Global AA-HA!) approach** will be used to: **identify adolescent health needs through exploring the collected data; assess policies and practices already in place** and **identify gaps and needs for action to improve health.**

Thus, using the AA-HA! intervention menu, students, teachers, and local authorities will work together to find and prioritize policy and programme solutions based on the survey's results. By the end of the workshop, the different stakeholders will establish a unified action plan to be implemented over the next 2 years and the evaluation and reporting approach.

The following agenda is general for all cities and will be adapted based on the culture and the needs of each individual city. It has a hands-on approach, with little use of technology. The **activities** listed under notes, such as the ice breaking & team building activities, **might change depending on each city**. The attached activities instruction pages are based on the timeline of this agenda.

For peer review only - http://bmjopen.bmj.com/site/about/guidelines.xhtml

Day 1

Total duration: 08:30h

Time	Program	Notes	Materials
08:00 - 08:15	**Registration**		*Tags, pens & snacks*
08:15 - 08:30	**Session 1: Welcome and opening remarks**	*From each stakeholder*	
08:30 - 09:00	Ice breaking activity	*In small groups, participants find 5 things in common between all of them*	*Colored papers & pens*
09:00 - 09:20	Participant's expected goals of the workshop	*Activity 1: Participants write goals answering the question: What is the goal of this workshop?*	*Flipchart to each group and post its*
09:20 - 09:30	Open-ended discussion of activity 1		
09:30 - 09:45	**Coffee Break**		
09:45 - 10:00	Short presentation of the workshop's background & objectives (CN)		*Printouts of day schedule*
10:00 - 10:30	**Team building activity**	*Participants should tell a short story which they draw on fold paper in shape of a box*	*Foldable papers*
10:30 - 11:00	Participants identify areas of improvements	*Activity 2: In groups, participants answer questions: **Which** areas they would prioritize as having potential for improvement? **What** is the status quo?*	*Flipcharts & pens*
11:00 - 11:05	**Energizing activity**		
11:05 - 11:45	Presentation & discussion of activity 2 results		
11:45 - 12:00	**Coffee Break**		
12:00 - 12:10	**Welcoming students**	*Students arrive to workshop*	
12:10 - 12:40	Presentation of photovoice taken by students	*Students and participants walk around and exchange thoughts of the photovoice displayed*	*Photovoice*
12:40 - 13:10	Photovoice feedback: open ended discussion	*Open ended discussion of their notes on the photovoice*	

For peer review only - http://bmjopen.bmj.com/site/about/guidelines.xhtml

13:10 - 14:10	**Lunch break**		
14:10 - 14:20	**Session 2: Opening remarks on defining aims**		
14:20 - 14:40	Presentation of the results of the health behavior survey (GSHS)		*Printouts of factsheets in easy-to-understand format*
14:40 - 15:20	Identification of areas of improvement, answering the question "Why" these areas and what are the opportunities based on GSHS results	*Activity 3: In groups they share thoughts regarding the survey (possibly with students)*	
15:20 - 15:30	**Coffee Break**		
15:30 - 15:50	Presentation of the results of school policies and practices (G-SHPPS)		*Printouts of factsheets in easy-to-understand format*
15:50 - 16:30	Identification of areas of improvement, answering the question "Why" these areas and what are the opportunities based on G-SHPPS results	*Activity 4: In groups they share thoughts regarding the survey (possibly with students)*	
16:30 -17:30	Identify and summarize final needs of the city based on the results of the 3 activities (data, photovoice, and the stakeholder's own opinions). Followed by closing remarks.	*Wrap up*	*Flipchart & pens*
17:30 - 18:00	**Apero (optional)**		*Snacks and coffee*

Day 2

Total duration: 8:30h

**Time**	**Program**	***Notes***	***Materials***
08:00 - 08:05	**Registration**	*Tags distribution*	*Snack, pens and papers*
08:05 - 08:10	**Session 1: Opening remarks of day 2**		
08:10 - 08:20	Participants share thoughts about workshop so far & expectations of day 2		*Printouts of day schedule*
08:20 - 08:45	Review of day 1 findings		
08:45 - 09:00	**Coffee break**		
09:00 - 09:30	Discussion of evidence-based interventions: Participants **review** current evidence-based	*Activity 1: What must be done? Groups phrase each aim separately*	*Flipcharts, post its and pens*

For peer review only - http://bmjopen.bmj.com/site/about/guidelines.xhtml

	interventions as presented in the Global AA-HA! and a recent meta-analysis		
09:30 - 10:30	**Identify** possible intervention ideas in their city		
10:30 - 10:40	**Energizing activity**		
10:40 - 11:15	What are their key findings	*Groups present activity 1*	
11:15 - 11:30	**Coffee break**		
11:30 - 12:30	Re-visiting intervention ideas:<br>Participants **specify** which of their ideas are most realistic & possible, given the resources in each city	*Activity 2: Participants answer the question: **WHAT** are possible interventions based on identified resources. What is feasible? **How** to reach them?*	*Post its*
12:30 - 13:00	What are their key findings	*Groups present activity 2*	
13:00 - 14:00	**Lunch break**		
14:00 - 14:10	**Session 2: Opening remarks on goal setting & prioritization**		
14:10 - 14:15	**Energizing activity**		
14:15 - 15:15	Participants share their findings on the interventions, students give feedback and add interventions if needed	*Activity 3: In groups, students review the identified interventions with the representatives*	
15:15 - 15:45	Each group presents their findings on activity 3		
15:45 - 16:00	**Coffee Break**		
16:00 - 16:40	Open ended discussion: summarize and conclude	*Students and representatives share final thoughts*	
16:40 - 16:45	**Energizing activity**		
16:45 - 17:15	Participants reflect on student's feedback and prioritize/update interventions	*Participants alone*	
17:15 - 17:30	**Closing remarks of day 2**		

For peer review only - http://bmjopen.bmj.com/site/about/guidelines.xhtml

Day 3

Total duration: 8:30h

Time	Program	*Notes*	*Materials*
08:00 - 08:05	**Registrations**	*Distribution of tags*	*Tags, paper and snacks*
08:05 - 08:10	**Session 1: Opening remarks of day 3**		
08:10 - 08:25	Participants share thoughts about workshop so far & expectations of day 3		*Printouts of day schedule*
08:25 - 08:45	Short summary of day 2		*Print outs*
08:45 - 09:00	Short presentation of logic model	*Interactive activity*	
09:00 - 09:15	**Coffee Break**		
09:15 - 10:00	Transferring results of the summary of day 2 in a logic model	*In groups, participants fill out the logic model*	*Logic model distribution*
10:00 - 10:10	**Energizing activity**		
10:10 - 11:30	Participants continue to fill logic model	*Open ended discussion*	
11:30 - 11:45	**Coffee Break**		
11:45 - 13:00	Measurements: What can be measured and how to proceed	*In groups, participants reflect on WHERE can the measurements be*	
13:00 - 14:00	**Lunch break**		
14:00 - 14:05	**Session 2: Opening Remarks**		
14:05 - 15:00	Participants plan timeline for the interventions	*In groups, participants plan a timeline to reach the interventions set*	*Flipchart, post its and pens*
15:00 - 16:00	Interventions feedback (students feedback)	*Group presentation*	
16:00 - 16:15	**Coffee Break & Energizing activity**		
16:15 - 17:00	The different stakeholders will establish a unified action plan to be implemented over the next 2 years as well as the evaluation and reporting approach	*Open ended discussion*	
17:00 -17:15	Congratulations and closing remarks on what the workshop participants achieved		
17:15 -17:30	**Feedback on workshop**		*Print outs*

For peer review only

SPIRIT

STANDARD PROTOCOL ITEMS: RECOMMENDATIONS FOR INTERVENTIONAL TRIALS

SPIRIT 2013 Checklist: Recommended items to address in a clinical trial protocol and related documents*

Section/item	ItemNo	Description	Page #/paragraph
**Administrative information**			
Title	1	Descriptive title identifying the study design, population, interventions, and, if applicable, trial acronym	Page 1
Trial registration	2a	Trial identifier and registry name. If not yet registered, name of intended registry	Page 5, Trial registration
	2b	All items from the World Health Organization Trial Registration Data Set	Page 5, Trial registration
Protocol version	3	Date and version identifier	Supplementary material: study protocol
Funding	4	Sources and types of financial, material, and other support	Page 24, Funding
Roles and responsibilities	5a	Names, affiliations, and roles of protocol contributors	Page 1-2; Page 24, Contributors
	5b	Name and contact information for the trial sponsor	Page 24, Funding; Supplementary material: donor decision letter
	5c	Role of study sponsor and funders, if any, in study design; collection, management, analysis, and interpretation of data; writing of the report; and the decision to submit the report for publication, including whether they will have ultimate authority over any of these activities	Page 24, Funding
	5d	Composition, roles, and responsibilities of the coordinating centre, steering committee, endpoint adjudication committee, data management team, and other individuals or groups overseeing the trial, if applicable (see Item 21a for data monitoring committee)	Page 24, Authors' contributions; Supplementary material: study protocol

For peer review only

Introduction

Background and rationale	6a	Description of research question and justification for undertaking the trial, including summary of relevant studies (published and unpublished) examining benefits and harms for each intervention	Page 7-9, Introduction
	6b	Explanation for choice of comparators	Page 10, Study design
Objectives	7	Specific objectives or hypotheses	Page 9, Introduction, last paragraph
Trial design	8	Description of trial design including type of trial (eg, parallel group, crossover, factorial, single group), allocation ratio, and framework (eg, superiority, equivalence, noninferiority, exploratory)	Page 10, Study design

Methods: Participants, interventions, and outcomes

Study setting	9	Description of study settings (eg, community clinic, academic hospital) and list of countries where data will be collected. Reference to where list of study sites can be obtained	Page 10, Study design
Eligibility criteria	10	Inclusion and exclusion criteria for participants. If applicable, eligibility criteria for study centres and individuals who will perform the interventions (eg, surgeons, psychotherapists)	Page 11, Study population and sampling
Interventions	11a	Interventions for each group with sufficient detail to allow replication, including how and when they will be administered	Page 15-18, Interventions
	11b	Criteria for discontinuing or modifying allocated interventions for a given trial participant (eg, drug dose change in response to harms, participant request, or improving/worsening disease)	N/A
	11c	Strategies to improve adherence to intervention protocols, and any procedures for monitoring adherence (eg, drug tablet return, laboratory tests)	Page 18, Monitoring and evaluation
	11d	Relevant concomitant care and interventions that are permitted or prohibited during the trial	N/A
Outcomes	12	Primary, secondary, and other outcomes, including the specific measurement variable (eg, systolic blood pressure), analysis metric (eg, change from baseline, final value, time to event), method of aggregation (eg, median, proportion), and time point for each outcome. Explanation of the clinical relevance of chosen efficacy and harm outcomes is strongly recommended	Page 12-14, Data collection and measures; Tables 1 and 2

For peer review only - http://bmjopen.bmj.com/site/about/guidelines.xhtml

Participant timeline	13	Time schedule of enrolment, interventions (including any run-ins and washouts), assessments, and visits for participants. A schematic diagram is highly recommended (see Figure)	Page 10, Study design; Figure 1; Page 22, Discussion, 2nd paragraph
Sample size	14	Estimated number of participants needed to achieve study objectives and how it was determined, including clinical and statistical assumptions supporting any sample size calculations	Page 11-12, Study population and sampling
Recruitment	15	Strategies for achieving adequate participant enrolment to reach target sample size	Page 11-12, Study population and sampling; Page 12-13, Data collection and measures

Methods: Assignment of interventions (for controlled trials)

Allocation:

Sequence generation	16a	Method of generating the allocation sequence (eg, computer-generated random numbers), and list of any factors for stratification. To reduce predictability of a random sequence, details of any planned restriction (eg, blocking) should be provided in a separate document that is unavailable to those who enrol participants or assign interventions	Page 10, Study design
Allocation concealment mechanism	16b	Mechanism of implementing the allocation sequence (eg, central telephone; sequentially numbered, opaque, sealed envelopes), describing any steps to conceal the sequence until interventions are assigned	Page 10, Study design
Implementation	16c	Who will generate the allocation sequence, who will enrol participants, and who will assign participants to interventions	Page 10, Study design; Page 11-12, Study population and sampling
Blinding (masking)	17a	Who will be blinded after assignment to interventions (eg, trial participants, care providers, outcome assessors, data analysts), and how	N/A
	17b	If blinded, circumstances under which unblinding is permissible, and procedure for revealing a participant's allocated intervention during the trial	N/A

Methods: Data collection, management, and analysis

Data collection methods	18a	Plans for assessment and collection of outcome, baseline, and other trial data, including any related processes to promote data quality (eg, duplicate measurements, training of assessors) and a description of study instruments (eg, questionnaires, laboratory tests) along with their reliability and validity, if known. Reference to where data collection forms can be found, if not in the protocol	Page 12-15, Data collection and measures; Supplementary material: study protocol; Appendix
	18b	Plans to promote participant retention and complete follow-up, including list of any outcome data to be collected for participants who discontinue or deviate from intervention protocols	Page 18, Monitoring and evaluation
Data management	19	Plans for data entry, coding, security, and storage, including any related processes to promote data quality (eg, double data entry; range checks for data values). Reference to where details of data management procedures can be found, if not in the protocol	Page 18-20, Data management and analysis plan
Statistical methods	20a	Statistical methods for analysing primary and secondary outcomes. Reference to where other details of the statistical analysis plan can be found, if not in the protocol	Page 18-20, Data management and analysis plan
	20b	Methods for any additional analyses (eg, subgroup and adjusted analyses)	Page 18-20, Data management and analysis plan
	20c	Definition of analysis population relating to protocol non-adherence (eg, as randomised analysis), and any statistical methods to handle missing data (eg, multiple imputation)	Page 18-20, Data management and analysis plan
**Methods: Monitoring**			
Data monitoring	21a	Composition of data monitoring committee (DMC); summary of its role and reporting structure; statement of whether it is independent from the sponsor and competing interests; and reference to where further details about its charter can be found, if not in the protocol. Alternatively, an explanation of why a DMC is not needed	Page 18-20, Data management and analysis plan; Reference to standard procedures as per GSHS manual; Page 24, Funding

	21b	Description of any interim analyses and stopping guidelines, including who will have access to these interim results and make the final decision to terminate the trial	Page 15-18, Interventions
Harms	22	Plans for collecting, assessing, reporting, and managing solicited and spontaneously reported adverse events and other unintended effects of trial interventions or trial conduct	Page 18, Monitoring and evaluation
Auditing	23	Frequency and procedures for auditing trial conduct, if any, and whether the process will be independent from investigators and the sponsor	Page 18, Monitoring and evaluation; Page 24, Funding
**Ethics and dissemination**			
Research ethics approval	24	Plans for seeking research ethics committee/institutional review board (REC/IRB) approval	Page 23, Ethics
Protocol amendments	25	Plans for communicating important protocol modifications (eg, changes to eligibility criteria, outcomes, analyses) to relevant parties (eg, investigators, REC/IRBs, trial participants, trial registries, journals, regulators)	Page 21-22, Discussion
Consent or assent	26a	Who will obtain informed consent or assent from potential trial participants or authorised surrogates, and how (see Item 32)	Page 11, Study population and sampling; Page 14-15, Physical activity validation study; Page 23, Ethics
	26b	Additional consent provisions for collection and use of participant data and biological specimens in ancillary studies, if applicable	N/A
Confidentiality	27	How personal information about potential and enrolled participants will be collected, shared, and maintained in order to protect confidentiality before, during, and after the trial	Page 18-20, Data management and analysis plan
Declaration of interests	28	Financial and other competing interests for principal investigators for the overall trial and each study site	Page 24, Competing interests

For peer review only - http://bmjopen.bmj.com/site/about/guidelines.xhtml

Access to data	29	Statement of who will have access to the final trial dataset, and disclosure of contractual agreements that limit such access for investigators	Page 20, Dissemination
Ancillary and post-trial care	30	Provisions, if any, for ancillary and post-trial care, and for compensation to those who suffer harm from trial participation	N/A
Dissemination policy	31a	Plans for investigators and sponsor to communicate trial results to participants, healthcare professionals, the public, and other relevant groups (eg, via publication, reporting in results databases, or other data sharing arrangements), including any publication restrictions	Page 20, Dissemination
	31b	Authorship eligibility guidelines and any intended use of professional writers	Page 24, Contributors
	31c	Plans, if any, for granting public access to the full protocol, participant-level dataset, and statistical code	Page 20, Dissemination
**Appendices**			
Informed consent materials	32	Model consent form and other related documentation given to participants and authorised surrogates	Supplementary material: Study protocol; Appendix
Biological specimens	33	Plans for collection, laboratory evaluation, and storage of biological specimens for genetic or molecular analysis in the current trial and for future use in ancillary studies, if applicable	N/A

*It is strongly recommended that this checklist be read in conjunction with the SPIRIT 2013 Explanation & Elaboration for important clarification on the items. Amendments to the protocol should be tracked and dated. The SPIRIT checklist is copyrighted by the SPIRIT Group under the Creative Commons "Attribution-NonCommercial-NoDerivs 3.0 Unported" license.

For peer review only - http://bmjopen.bmj.com/site/about/guidelines.xhtml

BMJ Open

Effectiveness of a participatory approach to develop school health interventions in four low resource cities: Study protocol of the "Empowering adolescents to lead change using health data" cluster randomized controlled trial

Journal:	*BMJ Open*
Manuscript ID	bmjopen-2022-071353.R1
Article Type:	Protocol
Date Submitted by the Author:	02-Jun-2023
Complete List of Authors:	Guthold, Regina ; WHO, Maternal, Newborn, Child and Adolescent Health and Ageing Department<br>Kann, Laura; WHO, Department of Noncommunicable Diseases<br>Bhatti, Lubna; WHO, Department of Noncommunicable Diseases<br>Abduvahobov, Parviz; UNESCO, Section of Health and Education, Education Sector<br>Ansong, Joana; WHO Country Office for Ghana<br>Atkinson, Uki; National Council on Drug Abuse<br>Baltag , Valentina; World Health Organisation, Maternal, Newborn, Child and Adolescent Health and Ageing Department<br>Caffe, Sonja; PAHO, Family, Health Promotion and Life Course<br>Caixeta, Roberta ; Pan American Health Organization, Noncommunicable Diseases and Mental Health Department<br>Diallo, Cheick Bady; WHO Regional Office for Africa, Universal Health Coverage/Communicable and Noncommunicable Diseases<br>Fouad, Heba; WHO Regional Office for the Eastern Mediterranean, Noncommunicable Diseases and Mental Health Department<br>Haddad, Sally; University of Bern, Department of Health Science, Institute of Sport Science<br>Hachri, Hafid; WHO Country Office for Morocco<br>Jaggi, Jeannine; University of Bern, Department of Health Science, Institute of Sport Science<br>Joshi, Pradeep; WHO Country Office for India<br>Karna, Priya; WHO Country Office for India<br>Louazani, Sidi Allal; WHO, Department of Noncommunicable Diseases<br>Mbola Mbassi, Symplice; WHO Regional Office for Africa, Universal Health Coverage/Life Course<br>Mehta, Rajesh; WHO Regional Office for South-East Asia<br>Mudgal, Yogendra; Department of School Education<br>Nigg, Claudio; University of Bern, Department of Health Science, Institute of Sport Science<br>Okely, Tony; University of Wollongong, School of Education<br>Ondarsuhu, Dolores; Pan American Health Organization, Noncommunicable Diseases and Mental Health Department<br>Ouaourir, Tahar; Ministry of Health, Population Department<br>Trhari, Fatima Zahra; Ministry of Health, Population Department<br>Riley, Leanne; World Health Organization, Department of Noncommunicable Diseases

**Primary Subject Heading**:	Global health
Secondary Subject Heading:	Evidence based practice, Public health
Keywords:	EPIDEMIOLOGIC STUDIES, PAEDIATRICS, Community child health < PAEDIATRICS, Adolescent, Clinical Trial

SCHOLARONE™ Manuscripts

TITLE PAGE

Effectiveness of a participatory approach to develop school health interventions in four low resource cities: Study protocol of the "*Empowering adolescents to lead change using health data*" cluster randomized controlled trial

Author names

Regina Guthold,[1] Laura Kann,[2] Lubna Bhatti,[2] Parviz Abduvahobov,[3] Joana Ansong,[4] Uki Atkinson,[5] Valentina Baltag,[1] Sonja Caffe,[6] Roberta de Betânia Caixeta,[7] Cheick Bady Diallo,[8] Heba Fouad,[9] Sally Haddad,[10] Hafid Hachri,[11] Jeannine Jaggi,[10] Pradeep Joshi,[12] Priya Karna,[12] Sidi Allal Louazani,[2] Symplice Mbola Mbassi,[13] Rajesh Mehta,[14] Yogendra Mudgal,[15] Claudio R. Nigg,[10] Anthony D. Okely,[16] Dolores Ondarsuhu,[7] Tahar Ouaourir,[17] Fatima Zahra Trhari,[17] Leanne M. Riley,[2]

Author affiliations

[1] Maternal, Newborn, Child and Adolescent Health and Ageing Department, WHO, Geneva, Switzerland

[2] Department of Noncommunicable Diseases, WHO, Geneva, Switzerland

[3] Section of Health and Education, Education Sector, UNESCO, Paris, France

[4] WHO Country Office for Ghana, Accra, Ghana

[5] National Council on Drug Abuse, Kingston, Jamaica

[6] Family, Health Promotion and Life Course, Pan American Health Organization, Washington D.C., USA

For peer review only

For peer review only - http://bmjopen.bmj.com/site/about/guidelines.xhtml

[7] Noncommunicable Diseases and Mental Health Department, Pan American Health Organization, Washington D.C., USA

[8] Universal Health Coverage/Communicable and Noncommunicable Diseases, WHO Regional Office for Africa, Brazzaville, Congo

[9] Noncommunicable Diseases and Mental Health Department, WHO Regional Office for the Eastern Mediterranean, Cairo, Egypt

[10] Department of Health Science, Institute of Sport Science, University of Bern, Bern, Switzerland

[11] WHO Country Office for Morocco, Rabat, Morocco

[12] WHO Country Office for India, New Delhi, India

[13] Universal Health Coverage/Life Course, WHO Regional Office for Africa, Brazzaville, Congo

[14] WHO Regional Office for South-East Asia, New Delhi, India

[15] Department of School Education, Jaipur, India

[16] School of Health and Society and Early Start, University of Wollongong, Australia and Illawarra Health and Medical Research Institute, Wollongong, Australia

[17] Population Department, Ministry of Health, Rabat, Morocco

Corresponding author

Address correspondence to: Regina Guthold, Ph.D., Maternal, Newborn, Child and Adolescent Health and Ageing Department, WHO, 20, Avenue Appia, CH-1211 Geneva, Switzerland.

E-mail address: gutholdr@who.int

For peer review only

For peer review only - http://bmjopen.bmj.com/site/about/guidelines.xhtml

Word count

Abstract: 356

Main text: 3,815

Number of tables, figures, and references

Tables: 2

Figures: 1

References: 40

Keywords

Adolescent health, Global health, Physical activity, Randomized controlled trial, Schools, Health Promotion

For peer review only - http://bmjopen.bmj.com/site/about/guidelines.xhtml

ABSTRACT

Introduction

Comprehensive local data on adolescent health are often lacking, particularly in lower resource settings. Furthermore, there are knowledge gaps around which interventions are effective to support healthy behaviours. This study generates health information for students from cities in four low- and middle-income countries to plan, implement and subsequently evaluate a package of interventions to improve health outcomes.

Methods and analysis

We will conduct a cluster randomized controlled trial in schools in Fez, Morocco; Jaipur, India; Saint Catherine Parish, Jamaica; and Sekondi-Takoradi, Ghana. In each city, approximately 30 schools will be randomly selected and assigned to the control or intervention arm. Baseline data collection includes three components. First, a Global School Health Policies and Practices Survey (G-SHPPS) to be completed by principals of all selected schools. Second, a Global School-based Student Health Survey (GSHS) to be administered to a target sample of n=3,153 13-17-year-old students of randomly selected classes of these schools, including questions on alcohol, tobacco and drug use, diet, hygiene, mental health, physical activity, protective factors, sexual behaviors, violence and injury. Third, a study validating the GSHS physical activity questions against wrist-worn accelerometry in one randomly selected class in each control school (n approximately 300 students per city). Intervention schools will develop a suite of interventions using a participatory approach driven by students and involving parents/guardians, teachers and community stakeholders. Interventions will aim to change existing structures and policies

For peer review only - http://bmjopen.bmj.com/site/about/guidelines.xhtml

at schools to positively influence students' behavior, using collected data and guided by the framework for *Making Every School a Health Promoting School*. Outcomes will be assessed for differential change after a two-year follow-up.

Ethics and dissemination

The study was approved by WHO's Research Ethics Review Committee; by the Jodhpur School of Public Health's Institutional Review Board for Jaipur, India; by the Noguchi Memorial Institute for Medical Research Institutional Review Board for Sekondi-Takoradi, Ghana; by the Ministry of Health and Wellness' Advisory Panel on Ethics and Medico-Legal Affairs for St Catherine, Jamaica, and by the Comité d'éthique pour la recherche biomédicale of the Université Mohammed V of Rabat for Fez, Morocco. Findings will be shared through open access publications and conferences.

Trial registration number

NCT04963426

For peer review only

For peer review only - http://bmjopen.bmj.com/site/about/guidelines.xhtml

STRENGTHS AND LIMITATIONS OF THIS STUDY

- This study generates comprehensive individual- and school-level adolescent health information for direct use to plan, implement and subsequently evaluate a package of interventions to improve health outcomes across four cities from different world regions, with a focus on underserved locations.
- The approach to develop interventions is data-informed, systematic and evidence-based, culturally adapted and flexible, and participatory, driven by adolescents, parents, and school- and community stakeholders.
- This study makes use of existing global tools for data collection, intervention planning and implementation and builds on local policies and practices as a foundation for action.
- The COVID-19 pandemic and related school closures have led to delays in study implementation.
- Internet connectivity may be poor in some study sites, and alternative methods for study components, such as electronic collection of school policy information, may need to be explored.

For peer review only

For peer review only - http://bmjopen.bmj.com/site/about/guidelines.xhtml

INTRODUCTION

Adolescence is a unique period in life with rapid physical, cognitive and psychosocial growth. It is an important time for laying foundations of future health, including, for example, establishing behaviors related to noncommunicable diseases (NCDs) such as tobacco and alcohol use, diet and physical activity, as well as behaviors linked to sexual or mental health (1). Implementing effective, evidence-based interventions could substantially reduce risk behaviors and support protective factors among adolescents and improve their future adult health (2, 3). However, several critical barriers exist to implementing such interventions for adolescents.

First, there is a lack of relevant and context-specific adolescent health information, particularly in low- and middle-income countries (LMICs), that is essential to inform intervention planning (4). Where data exist, they are primarily national-level estimates on specific health conditions that often mask variations and unequal progress by smaller geographical units (5, 6). Local data are needed to uncover and address these inequalities and develop the most relevant, effective, age-appropriate and context-specific interventions for adolescents. This includes data on policies and practices affecting adolescent's behavior, and on their health behaviors and protective factors. Collecting this information is of particular importance in usually neglected urban settings where little data exist, such as cities other than capitals (called 'secondary cities' hereafter).

Second, information on adolescent health is sometimes collected with questionnaires that may not have been validated for the populations in which they are used. Physical activity is one example where the most internationally used questions for adolescents have only been tested for validity in a few high-income countries (7, 8). To produce useful data to inform decision

For peer review only - http://bmjopen.bmj.com/site/about/guidelines.xhtml

making, it is essential for these questions to be tested more widely. Another reason to test adolescent physical activity questions is related to the change in recommended levels of physical activity for this age group. Most currently used questions are based on the 2010 WHO guidelines on physical activity that recommended adolescents to be physically active for at least 60 minutes *each* day (9). In 2020, these guidelines were updated and now state that adolescents should engage in at least *an average* of 60 minutes of activity per day throughout the week (10). Consequently, there is a need for adolescent physical activity questions to be tested against the updated guidelines.

Third, there are significant knowledge gaps about which interventions might be effective in targeting risk behaviors and supporting protective factors in adolescents (11). Available evidence is strongest for universal school-based interventions targeting multiple risk behaviors, however, most of the included studies were conducted in high-income countries, while evidence from LMICs is lacking (11).

Fourth, adolescents are too rarely involved in health promotion planning, decision-making and implementation. Yet, participatory approaches engaging adolescents are crucial for sustainable change in cognitions and behavior (12, 13) and thus warranted.

Global tools that help overcome the aforementioned barriers exist. The Global School Health Policies and Practices Survey (G-SHPPS) generates important school-level policy information (14). The Global School-based Student Health Survey (GSHS) collects data on students' health behaviors and protective factors (15). The Global Accelerated Action for the Health of Adolescents (Global AA-HA!) provides a systematic, participatory approach to understanding adolescent health needs based on data, prioritizing these needs in the local context and

For peer review only

For peer review only - http://bmjopen.bmj.com/site/about/guidelines.xhtml

planning, implementing, monitoring and evaluating appropriate interventions (16). The initiative *Making Every School a Health Promoting School* provides an evidence-based framework for the implementation of eight Global Standards to improve students' health (17) and promotes a whole-school approach that has demonstrated positive effects on health (18, 19, 20, 21). Both the Global AA-HA! and *Making Every School a Health Promoting School* emphasize the importance of students' involvement in health promotion planning, decision-making and implementation in a manner that is empowering. Finally, photovoice, facilitates adolescent engagement by allowing them to pictorially take impressions of health facilitators and barriers in their schools and community to inform intervention planning (22, 23, 24). These available global tools will be used to achieve the goals of this study. The overarching aim is to generate health information for students of secondary cities from four LMICs of different world regions that will be directly used to plan, implement, and evaluate a package of interventions for programme planning, policy, and structural change to improve students' health.

Specific objectives include: First, to assess current health policies and practices in selected schools of the four cities. Second, to understand the levels of health risk and protective factors among school-going adolescents in these cities. Third, to validate currently used questions to assess students' physical activity behavior with wearable accelerometers. Fourth, to plan and evaluate a participatory intervention approach that focuses on changing existing structures, policies, and practices and on implementing programmes in and around schools to improve health outcomes for students.

For peer review only - http://bmjopen.bmj.com/site/about/guidelines.xhtml

METHODS AND ANALYSIS

Study design and locations

We will conduct a multi-site two-armed cluster randomized controlled trial using a nested cross-sectional design. The clusters are defined as schools and classes within schools. The study locations are spread across four world regions and include the cities of Fez, Morocco; Jaipur, India; Saint Catherine Parish, Jamaica; and Sekondi-Takoradi, Ghana. These locations (secondary cities) were purposefully selected in collaboration with WHO regional focal points to generate information for settings that are less likely to be in the focus or receive resources for adolescent health interventions.

In each city, approximately 30 schools will be selected and randomly assigned to either the intervention or control group. At baseline, all sampled schools will conduct a G-SHPPS to assess school policies and practices (14) and a GSHS to assess risk behaviors and protective factors among students (15). Additionally, in the control group of each city, we will conduct studies to validate GSHS physical activity questions against an accelerometer (Axivity AX3 (25)) to be worn by students on the wrist for eight days prior to the baseline survey.

Following the baseline surveys, the intervention schools will participate in data-to-action workshops to develop a package of interventions. Students, parents, teachers, local authorities and researchers will work together to find and prioritize policy and programme solutions to establish a unified action plan. Subsequently, these interventions will be implemented, and study processes and outcomes will be assessed for differential change via repeat G-SHPPS and GSHS surveys after a two-year follow-up. At follow up, new classes will be selected from the same schools to ensure participation of 13–17-year-old students similar in age to the baseline

For peer review only - http://bmjopen.bmj.com/site/about/guidelines.xhtml

sample. We hypothesize that the prevalence of behavioral risk factors would be lower and the prevalence of protective factors higher among students in schools in the intervention group compared to the control group at follow-up. An overview of our study design is shown in Figure 1.

Study population and sampling

This study's target population is 13- to 17-year-old students. In each city, following standard G-SHPPS and GSHS sampling procedures (14, 15), approximately 30 schools will be randomly selected from a list of schools including every school in the city with eligible classes (classes that the target population usually attends), provided by education authorities from the respective cities. Selection will be done by experts from WHO with probability proportional to the number of students in the eligible schools.

For the GSHS, in a second sampling stage, classes will be randomly selected from all eligible classes. All students of selected classes will be asked to participate in the survey.

The city sample size was calculated to test the hypothesis that the prevalence of behavioral risk factors would be lower among students in the schools of the intervention group versus those in the control group at follow-up using G*Power 3.1 (26). With most outcome variables (prevalence of behavioral risk and protective factors) being binary, logistic regression was selected as the statistical test, the required power level was set at 80%, and the significance level pre-specified at α=0.05. The to-be-detected effect was specified at an Odds Ratio = 0.75, which represents the average of pooled effect sizes from a recent meta-analysis (11)

For peer review only - http://bmjopen.bmj.com/site/about/guidelines.xhtml

considering universal school interventions across a range of behaviors, similar to the ones assessed in our study.

The initial sample size calculation resulted in n=473, which was inflated assuming a design effect of 1.5 and an expected response rate of 90%: 473*1.5/0.9 = 788. Finally, this sample size was quadrupled aiming at reporting for two age groups within both the intervention and control arm: 13-15 and 16-17 years, resulting in a target sample size of n=3,153.

Participation in the G-SHPPS and GSHS is voluntary. For the GSHS, in line with local policies, consent will be obtained from the students themselves and/or their parents/guardians (see Appendix for consent forms).

Data collection and measures

G-SHPPS and GSHS

The G-SHPPS questionnaire (2021 version) will assess school policies and practices (14) across seven broad topics (Table 1 and Appendix). Exemplary process indicators are presented under the topics in Table 1. School principals of the participating schools will be asked to electronically complete the questionnaire.

Table 1. Questionnaire modules of the Global School Health Policies and Practices Survey (G-SHPPS) and sample process indicators.

G-SHPPS module	Sample process indicators (Percentage of schools that…)
School Health Services	• routinely provide as part of the health services offered to students counselling to prevent violence (including intimate partner, violence, sexual violence, gender-based violence, bullying, and gang violence).<br>• routinely provide as part of the health services offered to students administration of recommended immunizations.<br>• routinely provide as part of the health services offered to students support for management of overweight and obesity.
School Physical environment	• provide soap and water at handwashing facilities for students.<br>• have improved sanitation facilities for students.

For peer review only - http://bmjopen.bmj.com/site/about/guidelines.xhtml

Food and Nutrition Services	• make sugar-sweetened carbonated soft drinks and other sugar-sweetened drinks available to students through the vending machines, stores, canteens, or snack bars on school premises.<br>• routinely make fruits and vegetables available to students through the school's food and nutrition services.<br>• price healthy foods and beverages in the vending machine, school store, canteen, or snack bar on school premises lower than unhealthy options.
Health Education	• teach health education.<br>• teach sexual and reproductive health and HIV transmission, prevention, and treatment to students.<br>• teach violence and bullying prevention to students.<br>• teach decision-making, problem-solving, goal-setting, and refusal skills to students to help them avoid or reduce health risks.
Physical education	• teach physical education.<br>• teach students as part of physical education the value of physical activity for health, enjoyment, challenge, self-expression, and/or social interaction.<br>• regularly provide recess or other physical activity breaks to students during the school day.
School Governance and Leadership	• have an official council, committee, or team responsible for implementing health promoting school policies, programs, and activities.<br>• regularly involve and engage parents/caregivers/families and students in developing health promoting school policies.
School Policies and Resources	• have a policy specifically about becoming or continuing to be a Health Promoting School.<br>• have a policy prohibiting use of at least some tobacco or nicotine products, alcohol, and Illicit drugs among students on school premises.<br>• regularly monitor, evaluate, or assess the quality of their health promoting policies, programs, or activities.

Students' health behaviors and protective factors will be assessed using the GSHS questionnaire (2021 version) (15) (see Appendix). The GSHS is a paper-based, self-administered questionnaire with generic answer sheets that students complete during one classroom period.

The GSHS includes ten core questionnaire modules. Table 2 presents these modules with exemplary primary outcome indicators. Each city will be encouraged to include all modules. However, up to four modules may be excluded if questions of a specific module are deemed too sensitive. Cities may add context-specific questions, not exceeding a total of 80 questions.

Anthropometric measurements of height and weight are taken prior to survey administration. Students receive their measurement results and are asked to subsequently record them on the questionnaire.

Inclusion of the physical activity module will be mandatory to allow for validity testing of some of these questions. Additionally, a new question will be included for validation against the

For peer review only

updated WHO physical activity guidelines (10) reading *"During the past 7 days, did you do at least an average of 60 minutes per day of physical activity across the 7 days?"*.

Participating cities will also be encouraged to include the standard GSHS questions on attending school from home during the pandemic, missing classes or school without permission, and on school attendance under the influence of alcohol and of drugs.

Once the questionnaire has been finalized for each city, the same questionnaire will be anonymously self-administered by all students.

Table 2. Questionnaire modules of the Global School-based Student Health Survey (GSHS) and sample primary outcome indicators.

GSHS module	Sample outcome indicators, dependent on the city's choice of modules (Percentage of students who…)
Alcohol use	• currently drank alcohol (at least one drink of alcohol on at least one day during the 30 days before the survey).<br>• ever drank so much alcohol that they were really drunk one or more times during their life.<br>• had their first drink alcohol other than a few sips before age 14 years , among students who ever had a drink of alcohol other than a few sips.
Dietary behaviors	• were underweight (<-2SD from median for BMI by age and sex) (measured).<br>• were overweight (>+1SD from median for BMI by age and sex) (measured).<br>• were obese (>+2SD from median for BMI by age and sex) (measured).<br>• usually drank carbonated soft drinks one or more times per day during the 30 days before the survey.
Drug use	• ever used cannabis one or more times during their life.<br>• used drugs before age 14 years for the first time, among students who ever used drugs.
Hygiene	• usually cleaned or brushed their teeth less than one time per day during the 30 days before the survey.<br>• never or rarely washed their hands after using the toilet or latrine during the 30 days before the survey.
Mental health	• seriously considered attempting suicide during the 12 months before the survey.<br>• attempted suicide one or more times during the 12 months before the survey.<br>• did not have any close friends.
Physical activity	• were physically active for a total of at least 60 minutes per day on all 7 days during the 7 days before the survey.<br>• attended physical education classes on three or more days each week during this school year.<br>• spent three or more hours per day during a typical or usual day sitting or lying down (doing such things as watching television, playing computer games, talking with friends, using their mobile phone, traveling in a motor vehicle, napping, or doing other activities sitting or lying down) when not in school or doing homework or sleeping at night.
Protective factors	• missed classes or school without permission on one or more days during the 30 days before the survey.<br>• reported that their parents or guardians most of the time or always understood their problems and worries during the 30 days before the survey.<br>• reported that their parents or guardians most of the time or always really knew what they were doing with their free time during the 30 days before the survey.
Sexual behaviors	• ever had sexual intercourse.<br>• had sexual intercourse before age 14 years for the first time, among students who ever had sexual intercourse.<br>• used a condom during last sexual intercourse, among students who ever had sexual intercourse.
Tobacco use	• currently used any tobacco products (used any tobacco products on at least 1 day during the 30 days before the survey).<br>• currently smoked cigarettes (smoked cigarettes on at least 1 day during the 30 days before the survey).

For peer review only - http://bmjopen.bmj.com/site/about/guidelines.xhtml

	• currently used electronic cigarettes (used e-cigarettes on at least 1 day during the 30 days before the survey).
Violence and unintentional injury	• were in a physical fight one or more times during the 12 months before the survey.<br>• were seriously injured one or more times during the 12 months before the survey.<br>• were bullied during the 12 months before the survey.

Additional measures

With the interventions of the present study aiming at improving students' health and including programme planning, policy, and structural change, it is hypothesized that a range of secondary outcomes would also be positively influenced. These secondary outcomes will depend on the topics and interventions selected by each city during the data-to-action workshop and will be identified upon finalization of each city's action plan. They may include school absenteeism and performance among learners in the intervention schools, improved health care seeking, secondary health outcomes and improved communication of adolescents with teachers and parents.

Physical activity validation study

In addition to the GSHS self-reported physical activity, activity data will be collected using the Axivity AX3 accelerometer. This device has previously been used in large-scale population-based (27, 28) and school-based studies (29). The Axivity AX3 records movement, and reports raw acceleration signals in real time, allowing for transparent analysis (30).

From each school in the control group, one class will be randomly selected to participate in testing the validity of questions currently used in the GSHS to assess student's physical activity, sedentary behavior and sleep, and the new question that was added to assess WHO's 2020 physical activity guidelines (10). Every student in the selected classes will be asked to participate (approximately 300 students per city).

For peer review only - http://bmjopen.bmj.com/site/about/guidelines.xhtml

Participation in the physical activity validation study is voluntary. Participating students will be asked to provide written assent, and their parents or guardians will be asked to provide written consent (see Appendix).

In the week prior to the GSHS baseline survey, selected students will wear the Axivity AX3 on their non-dominant wrist continuously for eight consecutive days (31). Trained personnel will instruct students to wear the waterproof device and to participate in all their normal activities. After having worn the accelerometer, students will be asked to participate in the GSHS survey at baseline. Axivity AX3 data will then be compared to data collected with the GSHS physical activity module and the added physical activity question.

Interventions

This study's intervention approach is embedded in the framework of the Global Standards for Health Promoting Schools and its concept of a whole-school approach to promoting health (18). Schools in the intervention arm will implement health promotion strategies to address the most critical health risk behaviors and protective factors, considering the eight Global Standards including government policies and resources; school policies and resources; school governance and leadership; school and community partnerships; school curriculum; school social-emotional environment; school physical environment; and school health services.

Following the baseline surveys, students in the intervention arm will receive instructions to participate in photovoice (22). This is an established (23, 24) qualitative participatory method through which adolescents can pictorially take impressions of health facilitators and barriers in

For peer review only

For peer review only - http://bmjopen.bmj.com/site/about/guidelines.xhtml

their school and community. Pictures will be uploaded onto a secure platform, reviewed and sorted by trained personnel, ensuring that no inappropriate photos are included.

To develop the interventions, data-to-action workshops will be held in each city (see Appendix for agenda). They will be facilitated by the local study coordinator and WHO and UNESCO personnel and include a minimum of three nominated students from each intervention school, teachers, parents, and up to five local authorities and researchers. The nomination of students for the workshops will be based on the student's interest in participating and ensure gender balance.

Data-to-action workshops will use the data collected during the baseline surveys, the photovoice pictures, and follow the Global AA-HA! approach and its three key steps (16): First, recognizing that the health needs are different in each city, a needs assessment will be done, using the GSHS data and the photovoice pictures. To facilitate this, for the workshop, data will be visualized in an easy-to-understand summary format, including fact sheets and infographics. Workshop participants will review the site-specific results to identify which conditions, health risks and determinants need to be addressed most urgently. Second, considering that the situation adolescents live in is different in each setting, in a landscape analysis, results from the local G-SHPPS will be reviewed, along with other existing programmes, policies and legislation. Similar to step one, results will be summarized in an easy-to-understand format and used to identify gaps and potential areas for improvement. Step two will also include a review of evidence-based interventions of the Global AA-HA! and a meta-analysis (11), prepared in a format that will speak to workshop participants. Third, specific actions will be identified to address the issues specific to each city, considering the outcomes of steps one and two.

For peer review only - http://bmjopen.bmj.com/site/about/guidelines.xhtml

Identified actions will be prioritized, considering availability of resources, effectiveness, feasibility, capacity to implement, cultural appropriateness, and focus on structural and systems-oriented changes.

This participatory approach will ensure that – while considering the adolescents' health needs and each city’s situation – the selected interventions will be based on evidence, be feasible, appropriate, acceptable and aligned with local priorities. For example, a set of interventions to decrease tobacco use might be selected for a city with a high prevalence of tobacco use among students. These might include individual- and interpersonal-level interventions, but also school policy changes such as the introduction of smoke-free school policies that would ultimately lead to sustained behavior change.

The workshop duration will be three days, and include students, parents and teachers for at least two hours per day. To ensure that adolescents are meaningfully engaged, existing guidelines on adolescent participation and civic engagement will be followed (32). Breakout sessions will be organized during the workshops for which the different stakeholders will be separated, to ensure that each stakeholder group gets their own space to express their views. The different views will be brought back to the plenary and integrated as appropriate. Students will also get the opportunity to comment on and add to actions proposed by other stakeholders.

Each city will develop a 2-year implementation plan based on the prioritized interventions. These will be classified by topic and implementation level, including structural, environmental, organizational, community, interpersonal and individual level. A team of local stakeholders will

For peer review only - http://bmjopen.bmj.com/site/about/guidelines.xhtml

be identified with clear responsibilities regarding implementation of the interventions, and additional technical support will be provided by WHO.

Monitoring and evaluation

As per the Global AA-HA! (16), each city's implementation plan will include process and outcome monitoring through data collected with the G-SHPPS and the GSHS. Progress in the implementation of interventions will be monitored by the team of local stakeholders and WHO on a periodic basis. For each agreed upon activity/intervention in the implementation plan, monthly reporting will include whether the activity is not started, in planning, in progress or implemented, with a section for next steps and who is responsible.

Schools in the control arm will operate under 'business as usual' within their specific context, that is, they will not receive any intervention selected during the data-to-action workshop. Follow-up G-SHPPS and GSHS surveys (the same as at baseline) will occur two years after the baseline surveys in all selected schools. For the GSHS, new classes from these schools will be randomly selected to ensure participation of 13-17 year old students similar in age to the baseline sample.

Data management and analysis plan

During electronic data collection through DataForm (LimeSurvey) (33), G-SHPPS data are uploaded to a secure server that will only be accessible by the city and WHO. Summary results of key indicators will be produced for each city.

For peer review only - http://bmjopen.bmj.com/site/about/guidelines.xhtml

GSHS data are collected on computer scannable answer sheets with no personal identifying information. Data will be processed using standard cleaning and generic data analysis programmes. A city-specific database will be generated and results will be displayed on a standard fact sheet including information on response rates, a summary of methods, and weighted prevalence estimates with confidence intervals (CIs) (15).

The Axivity AX3 data will be downloaded on-site and uploaded to a secure WHO server with unique identifiers, with no personal information. The unique identifier will be used to link these data to the student's GSHS physical activity data. Time spent in total physical activity, moderate- to vigorous-intensity physical activity, and in sedentary behaviour will be computed from the Axivity AX3 data for each student, using standard methods (34, 35). Agreement between the Axivity AX3 and the questionnaire data will be assessed using approaches consistent with previous studies (31, 36).

For GSHS and G-SHPPS follow-up surveys, the same data analysis procedures as at baseline will be used.

To assess effectiveness of the interventions, comparative analysis of changes of policies and programmes and of students' health behaviors between the intervention and control group will be undertaken. With most outcome variables being binary, most intervention effects will be estimated through adjusted odds ratios with 95% CIs, using random effects logistic regression to adjust for within-school clustering. Additionally, a score across multiple risk and protective behaviors will be computed for each city (depending on the selection of questionnaire modules in each city), and effectiveness of the intervention will also be tested using this score (37). Data analysts will be blinded to allocation of schools in the two arms.

For peer review only - http://bmjopen.bmj.com/site/about/guidelines.xhtml

The analysis will account for potential spillover effects as much as possible. Spillover effects are defined as benefits provided by interventions that extend beyond direct recipients and impact people in close physical or social proximity who did not directly receive the intervention themselves, namely students in the control arm (38).

Patient and Public Involvement Statement

Relevant local stakeholders will be engaged at all project stages to ensure that this research builds local capacity and is responsive to the health needs and priorities of the students in each city (39). Besides representatives of the national or local health and education authorities and local researchers, the study committees include community and school stakeholders. Student participants, parents/guardians, teachers, and community leaders will lead the use of the collected survey data to identify and implement interventions. Interventions will additionally be shaped by pictures that students provide of what they perceive as facilitators and barriers to their own health behavior.

Trial status

This trial began recruitment in January 2022. Baseline data collection started in the first city (Jaipur; India) in August 2022 and has been finalized in all four cities in May 2023. After the two-year intervention period and the follow-up assessments, the study completion date is expected in January 2026. WHO as the trial management body will provide oversight to this multi-city trial throughout its duration.

For peer review only - http://bmjopen.bmj.com/site/about/guidelines.xhtml

ETHICS AND DISSEMINATION

The master protocol and the four site-specific protocols have been approved by the WHO Research Ethics Review Committee (ERC.0003397). The four site-specific protocols have also been approved by the Jodhpur School of Public Health's Institutional Review Board for Jaipur, India; the Noguchi Memorial Institute for Medical Research Institutional Review Board for Sekondi-Takoradi, Ghana; the Ministry of Health and Wellness' Advisory Panel on Ethics and Medico-Legal Affairs for St Catherine, Jamaica, and the Comité d'éthique pour la recherche biomédicale of the Université Mohammed V of Rabat for Fez, Morocco. Following standard G-SHPPS procedures, permissions from all schools to take part in the study will be obtained prior to field work. For the GSHS, following standard procedures previously used in over 100 countries, all parents/guardians will be notified about the study, and parental/guardian consent will be obtained following local laws and policies. For the physical activity validation study, all parents/guardians will be notified and parental/guardian consent and/or student assent will be obtained in each city, adhering to local laws and policies.

The present study is minimally intrusive on the student's privacy, on schools and communities. All data collection tools have previously been tested and used in populations similar to the target population of this study. Through participatory planning and careful review, it will be ensured that the interventions are of minimal physical, psychological and social risk. It is expected that the target population will benefit from the outcome of the interventions, in the form of improved policies and practices, as well as improved behavioural outcomes. In each city, local stakeholders will determine appropriate compensation for participating students' time spent at the data-to-action workshop and to co-design interventions.

For peer review only

For peer review only - http://bmjopen.bmj.com/site/about/guidelines.xhtml

432 Following GSHS and G-SHPPS policy, data will be held at the country level, with a copy at WHO,
433 for an initial two-year period. During this time, each site will be encouraged to produce any
434 report and publication they desire. After this period, datasets will be made publicly available,
435 including in the WHO NCD microdata repository (40). GSHS and G-SHPPS results will be
436 presented in a summary report, including user-friendly infographics, for sharing with the
437 participating students, their parents, teachers and communities. Data and results from the
438 physical activity validation study will be stored on a WHO server and will be made available to
439 interested researchers upon request after the initial two-year period.

441 **Acknowledgements**

442 We thank Prof Susan Sawyer and Prof Antony Morgan for their thorough review of the study
443 protocol.

445 **Contributors**

446 RG wrote the first draft of this paper. RG and LMR conceptualized the study and developed the
447 methods. LK, LB and SAL contributed to the development of the methods and preparation for
448 sampling and data collection. PA contributed to the development of Table 2 and the methods
449 for the intervention. AO contributed to the development of the methods for the physical
450 activity validation study. CRN, SH, JJ and VB contributed to the development of the methods for
451 the intervention. JA, UA, SC, RBC, CD, HF, HH, PJ, PK, SMM, RM, YM, DO, TO and FZT coordinate
452 site-specific implementation and provided input on the manuscript from a site-specific
453 perspective. All authors have reviewed the paper and approved its final version.

For peer review only - http://bmjopen.bmj.com/site/about/guidelines.xhtml

For peer review only

Competing interests

The authors declare that they do not have competing interests.

Authors' information

RG, LK, LB, JA, VB, CD, HF, HH, PJ, PK, SAL, SMM, RM and LMR are members of the World Health Organization. SC, RBC and DO are members of the Pan American Health Organization. The authors alone are responsible for the views expressed in this publication and they do not necessarily represent the decisions, policy, or views of the World Health Organization or the Pan American Health Organization.

Funding

This work is supported by Fondation Botnar, Activity REG-19-015. The funder did not play a role in the design of this study.

Patient consent for publication

Not applicable.

Figure title and legend

Figure title

Figure 1. Overview of the study design.

For peer review only - http://bmjopen.bmj.com/site/about/guidelines.xhtml

475 **Figure legend**

476 [a] *GSHS - Global School-based Student Health Survey*

477 [b] *G-SHPPS - Global School Health Policies and Practices Survey*

For peer review only

For peer review only - http://bmjopen.bmj.com/site/about/guidelines.xhtml

REFERENCES

1. World Health Organization. Tackling NCDs. Best buys. Geneva, Switzerland: World Health Organization; 2017.
2. Watkins D, Hale J, Hutchinson B, Kataria I, Kontis V, Nugent R. Investing in non-communicable disease risk factor control among adolescents worldwide: a modelling study. BMJ global health. 2019;4(2):e001335.
3. Ginis KA, Nigg CR, Smith AL. Peer-delivered physical activity interventions: an overlooked opportunity for physical activity promotion. Translational behavioral medicine. 2013;3(4):434-43.
4. Azzopardi P, Kennedy E, Patton G. Data and indicators to measure adolescent health, social development and well-being. Florence, Italy: UNICEF Office of Research - Innocenti; 2017.
5. Neal S, Ruktanonchai C, Chandra-Mouli V, Matthews Z, Tatem AJ. Mapping adolescent first births within three east African countries using data from Demographic and Health Surveys: exploring geospatial methods to inform policy. Reproductive health. 2016;13(1):98.
6. Liang M, Simelane S, Fortuny Fillo G, Chalasani S, Weny K, Salazar Canelos P, et al. The State of Adolescent Sexual and Reproductive Health. The Journal of adolescent health : official publication of the Society for Adolescent Medicine. 2019;65(6s):S3-s15.
7. Ridgers ND, Timperio A, Crawford D, Salmon J. Validity of a brief self-report instrument for assessing compliance with physical activity guidelines amongst adolescents. Journal of science and medicine in sport. 2012;15(2):136-41.
8. Prochaska JJ, Sallis JF, Long B. A physical activity screening measure for use with adolescents in primary care. Archives of pediatrics & adolescent medicine. 2001;155(5):554-9.
9. World Health Organization. Global recommendations on physical activity for health. Geneva, Switzerland: World Health Organization; 2010.
10. World Health Organization. WHO guidelines on physical activity and sedentary behaviour. Geneva, Switzerland: World Health Organization; 2020.
11. MacArthur G, Caldwell DM, Redmore J, Watkins SH, Kipping R, White J, et al. Individual-, family-, and school-level interventions targeting multiple risk behaviours in young people. Cochrane Database Syst Rev. 2018;10(10):CD009927-CD.
12. Greene K. The theory of active involvement: processes underlying interventions that engage adolescents in message planning and/or production. Health communication. 2013;28(7):644-56.
13. Larsson I, Staland-Nyman C, Svedberg P, Nygren JM, Carlsson IM. Children and young people's participation in developing interventions in health and well-being: a scoping review. BMC health services research. 2018;18(1):507.
14. World Health Organization. Global School Health Policies and Practices Survey Geneva, Switzerland: World Health Organization; 2021 [Available from: https://www.who.int/teams/noncommunicable-diseases/surveillance/systems-tools/global-school-health-policies-and-practices-survey.
15. World Health Organization. Global School-based Student Health Survey (GSHS) 2021 [Available from: https://www.who.int/teams/noncommunicable-diseases/surveillance/systems-tools/global-school-based-student-health-survey.
16. World Health Organization. Global Accelerated Action for the Health of Adolescents (AA-HA!): guidance to support country implementation. Geneva, Switzerland; 2017.
17. World Health Organization; United Nations Educational; Scientific and Cultural Organization. Making every school a health-promoting school: implementation guidance. Geneva, Switzerland: World Health Organization; United Nations Educational; Scientific and Cultural Organization; 2021.
18. World Health Organization; United Nations Educational; Scientific and Cultural Organization. Making every school a health-promoting school: global standards and indicators for health-promoting

For peer review only - http://bmjopen.bmj.com/site/about/guidelines.xhtml

schools and systems. Geneva, Switzerland: World Health Organization and United Nations Educational, Scientific and Cultural Organization; 2021. Contract No.: 21 November.
19. Shinde S, Weiss HA, Varghese B, Khandeparkar P, Pereira B, Sharma A, et al. Promoting school climate and health outcomes with the SEHER multi-component secondary school intervention in Bihar, India: a cluster-randomised controlled trial. The Lancet. 2018;392(10163):2465-77.
20. Patton GC, Bond L, Carlin JB, Thomas L, Butler H, Glover S, et al. Promoting social inclusion in schools: a group-randomized trial of effects on student health risk behavior and well-being. Am J Public Health. 2006;96(9):1582-7.
21. Langford R, Bonell C, Jones H, Pouliou T, Murphy S, Waters E, et al. The World Health Organization's Health Promoting Schools framework: a Cochrane systematic review and meta-analysis. BMC Public Health. 2015;15(1):130.
22. PhotoVoice. PhotoVoice - Ethical photography for social change 2021 [Available from: https://photovoice.org/.
23. Fountain S, Hale R, Spencer N, Morgan J, James L, Stewart MK. A 10-Year Systematic Review of Photovoice Projects With Youth in the United States. Health promotion practice. 2021;22(6):767-77.
24. Catalani C, Minkler M. Photovoice: a review of the literature in health and public health. Health education & behavior : the official publication of the Society for Public Health Education. 2010;37(3):424-51.
25. Axivity. Axivity AX3 Newcastle, United Kingdom: Axivity; 2021 [Available from: https://axivity.com/product/ax3.
26. Faul F, Erdfelder E, Buchner A, Lang AG. Statistical power analyses using G*Power 3.1: tests for correlation and regression analyses. Behavior research methods. 2009;41(4):1149-60.
27. Doherty A, Jackson D, Hammerla N, Plötz T, Olivier P, Granat MH, et al. Large Scale Population Assessment of Physical Activity Using Wrist Worn Accelerometers: The UK Biobank Study. PloS one. 2017;12(2):e0169649.
28. Westgate K, Ridgway C, Rennie K, Strain T, Wijndaele K, Brage S. Feasibility of incorporating objective measures of physical activity in the STEPS program. A pilot study in Malawi. Cambridge, United Kingdom: MRC Epidemiology Unit, University of Cambridge; 2020.
29. Bonnesen CT, Toftager M, Madsen KR, Wehner SK, Jensen MP, Rosing JA, et al. Study protocol of the Healthy High School study: a school-based intervention to improve well-being among high school students in Denmark. BMC public health. 2020;20(1):95.
30. Troiano RP, McClain JJ, Brychta RJ, Chen KY. Evolution of accelerometer methods for physical activity research. British journal of sports medicine. 2014;48(13):1019-23.
31. Rääsk T, Mäestu J, Lätt E, Jürimäe J, Jürimäe T, Vainik U, et al. Comparison of IPAQ-SF and Two Other Physical Activity Questionnaires with Accelerometer in Adolescent Boys. PloS one. 2017;12(1):e0169527.
32. UNICEF. Engaged and heard! Guidelines on adolescent participation and civic engagement. New York, USA: UNICEF; 2020.
33. Lime Survey. Professional online surveys with LimeSurvey 2020 [Available from: https://www.limesurvey.org/.
34. Stewart T, Narayanan A, Hedayatrad L, Neville J, Mackay L, Duncan S. A Dual-Accelerometer System for Classifying Physical Activity in Children and Adults. Medicine and science in sports and exercise. 2018;50(12):2595-602.
35. Blythe A, Crouter SE, LaMunion SR. Validity of consumer-based physical activity monitors for estimating energy expenditure in youth. University of Tennessee Honors Thesis Project; 2017.
36. Bland JM, Altman DG. Statistical methods for assessing agreement between two methods of clinical measurement. Lancet (London, England). 1986;1(8476):307-10.

For peer review only - http://bmjopen.bmj.com/site/about/guidelines.xhtml

37. Prochaska JJ, Velicer WF, Nigg CR, Prochaska JO. Methods of quantifying change in multiple risk factor interventions. Preventive medicine. 2008;46(3):260-5.

38. Benjamin-Chung J, Arnold BF, Berger D, Luby SP, Miguel E, Colford JM, Jr., et al. Spillover effects in epidemiology: parameters, study designs and methodological considerations. International journal of epidemiology. 2018;47(1):332-47.

39. Council for International Organizations of Medical Sciences (CIOMS). International Ethical Guidelines for Health-related Research Involving Humans. Geneva, Switzerland; 2016.

[dataset] 40. World Health Organization. NCD Microdata Repository: World Health Organization; 2021 [Available from: https://extranet.who.int/ncdsmicrodata/index.php/home.

For peer review only

For peer review only - http://bmjopen.bmj.com/site/about/guidelines.xhtml

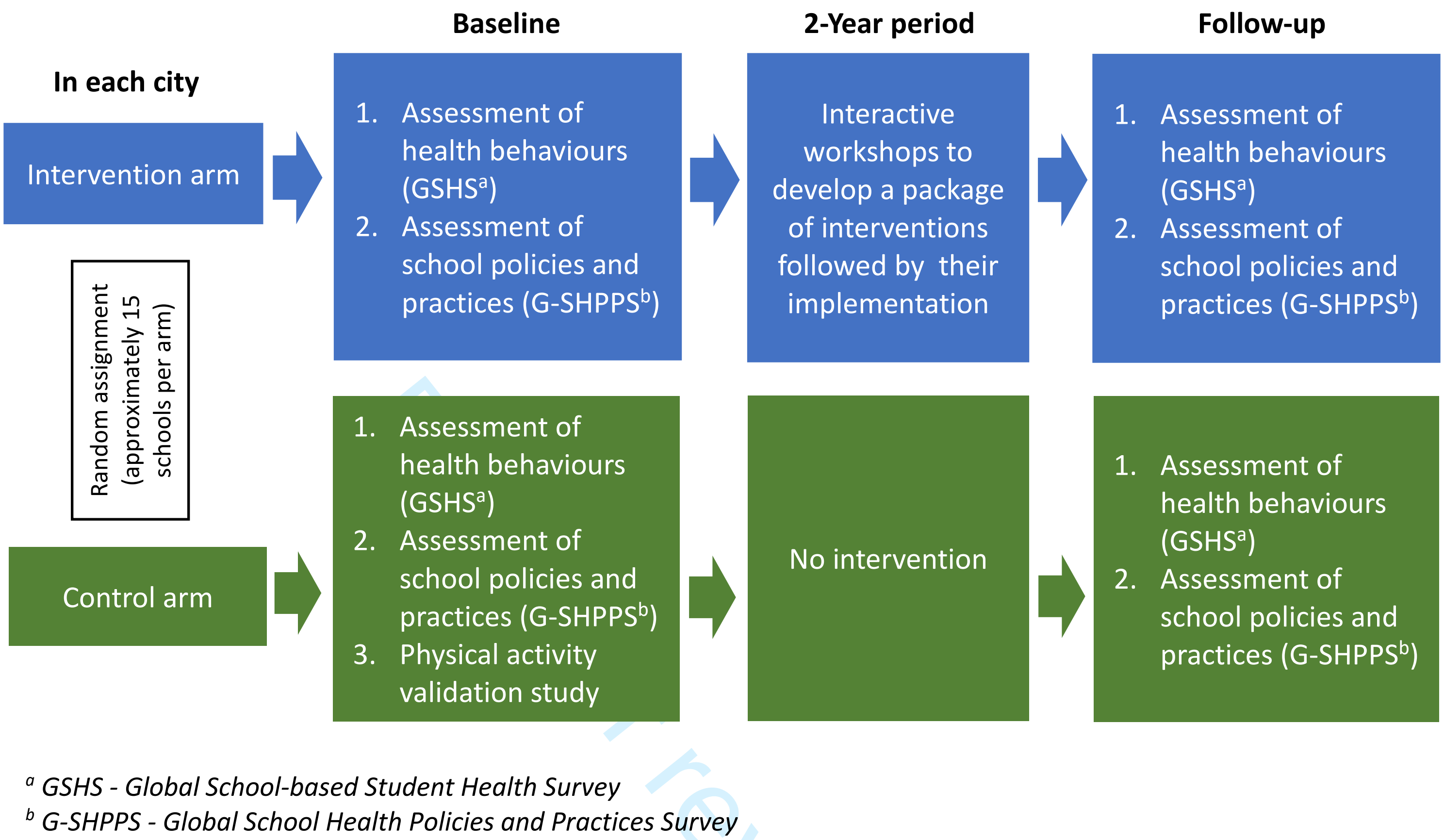

Figure 1. Overview of the study design.

For peer review only

Appendix

G-SHPPS questionnaire

GSHS questionnaire

Notification, permission and assent forms

Data-to-action workshop agenda

For peer review only

Global School Health Policies and Practices Survey (G-SHPPS)

2021

This questionnaire will be used to assess school health policies and practices across our country. Your cooperation is essential for making the results of this survey comprehensive, accurate, and timely. Your answers will be kept confidential. Individual school results will not be reported.

INSTRUCTIONS

1. This questionnaire should be completed by the principal or headteacher (or the person acting in that capacity) and concerns only activities that occur in this school and local community. Please feel free to consult with other people (such as a health education teacher or school nurse) if you are not sure of an answer.

2. For the purposes of this questionnaire, a "policy" or "school policy" is any **written** law, rule, regulation, administrative order, guideline, standard, or similar kind of mandate issued or created **by this school or a local, state, district, or federal agency or organization with authority over this school**. Schools may sometimes grant policy exceptions or waivers, but please answer each question based on what is considered the general policy and standard practice in this school.

3. For the purposes of this questionnaire, "health" is defined as a state of complete physical, mental, and social well-being and not merely the absence of disease or infirmity.

4. Follow the instructions for each question. If you are unsure about the meaning of a word or phrase that is bolded with an asterisk you will find a definition for it at the end of that question.

5. You may move forward and backward through the questionnaire by using the navigation buttons in the questionnaire. Do not use the forward and backward arrows on your browser.

6. If you need to stop responding and resume later, your responses will be saved. Just press the **Resume later** link at the bottom of the page before closing your browser. To resume please use the same URL or token provided initially to finalize your questionnaire.

7. Once you are comfortable with all your responses, click on the SUBMIT button at the end of the questionnaire.

8. Please answer every question.

9. If you have any questions about this survey, please contact the person identified on the email you were sent about this survey.

THANK YOU FOR YOUR ASSISTANCE.

For peer review only - http://bmjopen.bmj.com/site/about/guidelines.xhtml

Table of Contents

Page

1. **School Overview** 3

2. **School Health Services** 4
   - School health workers 4
   - Health services for students 5
   - Health information management 9
   - School entry requirements 9

3. **School Physical Environment** 10
   - Water, sanitation, and hygiene 10
   - Injury prevention and safety 11
   - Sun safety 11
   - Pest control 12

4. **Food and Nutrition Services** 13
   - Food preparation 14
   - Other foods and beverages available at school 15
   - Food and nutrition services environment 16

5. **Health Education** 17
   - Content of health education 18

6. **Physical Education** 20
   - Content of physical education 21
   - Facilities and equipment 21
   - Physical activity or recreation clubs and competitive sports 22

7. **School Governance and Leadership** 23
   - School health councils, committees, or teams 23
   - Community partnerships 24

8. **School Policies and Resources** 25
   - Overall curriculum 25
   - Professional development for teachers 26
   - Bullying and violence prevention 26
   - Tobacco use prevention 27
   - Alcohol use prevention 27
   - Illicit drug use prevention 28
   - Crisis preparedness and emergency response 28
   - Eye health 29
   - Pregnancy students 29

1. School Overview

1-01. Who attends this school?

A. All genders
B. Only males
C. Only females

1-02. How many students are enrolled in this school? _____

1-03. How many full-time teachers provide instruction to students in this school? _____

1-04. Are the following educational levels taught in this school? *(Mark yes or no for each level.)*

	Yes	No
A. Primary education (**ISCED 1**[1])	O	O
B. Lower secondary education (ISCED 2)	O	O
C. Upper secondary education (ISCED 3)	O	O

1-05. Is this a boarding school where students both learn and live?

A. Yes
B. No

1-06. How would you classify this school?

A. Government or public school
B. Non-government, religious, or private school
C. None of the above

1-07. Is this school located in a mostly rural or mostly urban setting?

A. Mostly rural
B. Mostly urban

1-08. Does this school have internet service reliable enough that it can be routinely used for student instruction?

A. Yes
B. No

[1] A widely-used global reference classification for education systems that is maintained and periodically revised by the UNESCO Institute for Statistics in consultation with Member States and other international and regional organizations. The International Standard for Classification of Education (ISCED) allows comparison of education systems across countries.

For peer review only - http://bmjopen.bmj.com/site/about/guidelines.xhtml

2. School Health Services

2-01. Which of the following statements **best** describes how **health services**[2] are regularly provided to students in this school?

A. Health services are provided both on school premises and at separate facilities (not on school premises) through a formal agreement with this school
B. Health services are provided only on school premises
C. Health services are provided only at separate facilities (not on school premises) through a formal agreement with this school to provide health services
D. Health services are only provided on an emergency basis **(Go to 2-15)**
E. I do not know **(Go to 2-15)**

2-02. Which of the following statements best describes how school policy addresses health services for students?

A. School policy provides a specific plan or guidelines for implementing health services for students
B. Health services are mentioned in a school policy, but there is no specific plan or guideline for implementing health services for students
C. Health services for students are not addressed in any school policy
D. I do not know

2-03. Does this school have someone officially responsible for managing or coordinating this school's health services?

A. Yes
B. No
C. I do not know

2-04. Does this school have the supplies needed for teachers and other school staff to apply **standard or universal precautions**[3], including disposable gloves and bandages?

A. Yes
B. No
C. I do not know

School health workers

2-05. Do the following types of **health workers**[4] provide health services to students in this school? *(Mark yes or no or I do not know for each type of health worker.)*

	Yes	No	I do not know
A. Nurses	O	O	O
B. Physicians	O	O	O
C. Psychologists, counsellors, or social workers	O	O	O
D. Dentists or dental hygienists	O	O	O
E. Nutritionists	O	O	O

[2] Services provided by a health worker to students enrolled in primary or secondary education, either on school premises or in a facility not on school premises that has a formal agreement with the school to provide health services to the school's students.
[3] A method of infection control in which all human blood, certain body fluids, and fresh tissues and cells of human origin are handled as if they are known to be infected with Human Immunodeficiency Virus (HIV), Hepatitis B Virus (HBV), and/or other blood-borne pathogens.
[4] A health worker is a person whose main function is to deliver health promotion, prevention, care, and/or treatment services to students, such as a nurse or clinical psychologist, but not a teacher.

For peer review only - http://bmjopen.bmj.com/site/about/guidelines.xhtml

2-06. On average, how many days per week is a health worker available to students in this school?

A. Less than 1 day
B. 1 day
C. 2 days
D. 3 days
E. 4 days
F. 5 or more days
G. I do not know

2-07. Does this school provide professional development opportunities to health workers to improve the quality of health services for students?

A. Yes
B. No
C. I do not know

Health services for students

2-08. As part of the health services offered to students, are the following **preventive interventions**[5] routinely provided? *(Mark yes or no or I do not know for each preventive intervention.)*

	Yes	No	I do not know
A. Routine preventive health check-ups	O	O	O
B. Administration of recommended immunizations (such as **diphtheria, HPV, tetanus, measles, and rubella**[6])	O	O	O
C. **Micronutrient**[7] supplements (such as iron, iodine, zinc, or Vitamin A)	O	O	O

2-09. Does this school provide information to students, parents, caregivers, and families about the value and importance of receiving routine immunizations to prevent infectious diseases?

A. Yes
B. No
C. I do not know

2-10. As part of the health services offered to students, are the following **screenings**[8] routinely provided for at least most students? *(Mark yes or no or I do not know for each screening.)*

	Yes	No	I do not know
A. Eye and vision	O	O	O
B. Ear and hearing	O	O	O
C. Oral health	O	O	O
D. Nutrition (such as for **anaemia, malnutrition, and obesity**[9]**)**	O	O	O

Note: If A, B, C, and D are all No or I do not know – Go to 2-12.

[5] A health intervention to prevent illness, disease, or injury.
[6] Diphtheria – A serious bacterial infection of the nose and throat. HPV – A sexually transmitted infection caused by the human papillomavirus that may lead to genital warts or cancer. Tetanus – A bacterial infection that causes painful muscle contractions particularly in the jaw and neck. Measles – A highly contagious viral disease characterized by a high fever and rash. Rubella – A viral disease characterized by a low fever, sore throat, and rash.
[7] Vitamins and minerals vital to healthy development, disease prevention, and well-being.
[8] Medical tests to check for diseases and health conditions before there are any signs or symptoms.
[9] Anaemia – A condition marked by a lack of red blood cells or of hemoglobin in the blood. Malnutrition – Lack of proper nutrition caused by not having enough to eat, not eating enough of the right things, or another disease. Obesity – A disorder involving excess body fat that increases the risk of other health problems.

For peer review only - http://bmjopen.bmj.com/site/about/guidelines.xhtml

2-11. Do the following actions typically occur when a student's screening indicates a potential problem? *(Mark yes or no or I do not know for each action.)*

	Yes	No	I do not know
A. The student is notified	O	O	O
B. The student's parents or caregiver are notified	O	O	O
C. The student's teachers are notified	O	O	O
D. A referral is provided for an appropriate health worker	O	O	O

2-12. As part of the health services offered to students, are the following **health promotion**[10] activities routinely provided? *(Mark yes or no or I do not know for each health promotion activity.)*

	Yes	No	I do not know
A. Timely care-seeking from an appropriate provider	O	O	O
B. Increased health literacy	O	O	O
C. Improved personal hygiene and handwashing with soap	O	O	O
D. Improved oral health care	O	O	O
E. Reduced consumption of sugar and **sugar-sweetened**[11] beverages	O	O	O
F. Increased physical activity and limited **sedentary behaviour**[12]	O	O	O
G. Appropriate use of electronic devices	O	O	O
H. Attainment of adequate sleep	O	O	O
I. Appropriate sun exposure for the context	O	O	O
J. Menstrual hygiene management	O	O	O

Note: Ask J only if females attend this school.

review only

[10] The process of enabling individuals to increase control over, and to improve, their health. Health promotion can happen formally or informally, in a group or one-on-one, and in a clinical setting.
[11] Sugar-sweetened beverages include carbonated soft drinks, sports drinks, energy drinks,100% fruit juices, fruit drinks that are not 100% juice, sugar-sweetened flavoured milks, and sugar-sweetened teas, coffees, or flavoured waters.
[12] Activities occurring in a reclining, seated, or lying position requiring very low energy expenditure.

For peer review only - http://bmjopen.bmj.com/site/about/guidelines.xhtml

2-13. As part of the health services offered to students, are the following health services in each health area routinely provided to students? Health services may include a clinical assessment and subsequent care and support or referral to another facility for care. *(Mark yes or no or I do not know for each health service.)*

	Yes	No	I do not know
**General/cross cutting**			
A. Provision of first aid	O	O	O
B. Administration of over-the-counter and prescribed medications	O	O	O
C. Control and management of pain (such as from a headache or toothache)	O	O	O
D. Management of non-specific symptoms (such as diarrhoea or fever)	O	O	O
**Positive health development**			
E. Identification of developmental difficulties and disabilities	O	O	O
F. Counselling related to physical and psychosocial development (such as puberty, skin changes, body image, or child marriage)	O	O	O
**Unintentional injury**			
G. Management of common childhood injuries (such as fractures or wounds)	O	O	O
H. Management of burns	O	O	O
I. Management of non-fatal drowning and related complications	O	O	O
**Violence**			
J. Counselling to prevent violence (including intimate partner violence, sexual violence, gender-based violence, bullying, and gang violence)	O	O	O
K. Support for victims of violence	O	O	O
**Sexual and reproductive health, including HIV**			
L. Contraceptive counselling	O	O	O
M. Counselling on prevention of HIV or sexually-transmitted infections	O	O	O
N. **HIV pre-exposure and/or post-exposure prophylaxis**[13]	O	O	O
O. HIV testing services	O	O	O
P. Management of sexually-transmitted infections	O	O	O
Q. Management of pregnancy (including the option to continue or return to school)	O	O	O

Note: Ask Q only if females attend this school.

w only

[13] Medicine taken to prevent getting HIV either before or after high risk behaviour.

For peer review only - http://bmjopen.bmj.com/site/about/guidelines.xhtml

2-14. As part of the health services offered to students, are the following health services in each health area routinely provided to students? Health services may include a clinical assessment and subsequent care and support or referral to another facility for care. *(Mark yes or no or I do not know for each health service.)*

	Yes	No	I do not know
**Communicable disease**			
A. Management of common infections (such as ear, eye, dental, skin, throat, or urinary tract infections)	O	O	O
B. Management of other infectious diseases (such as **cholera, dengue, dysentery, helminths, tuberculosis, or malaria**[14*])	O	O	O
C. Support for chronic care of HIV-infected children	O	O	O
**Noncommunicable disease, physical disability, and nutrition**			
D. Management of **anaemia**[15] (such as iron supplementation)	O	O	O
E. Support for management of overweight and **obesity**[16]	O	O	O
F. Management of asthma	O	O	O
G. Management of other chronic conditions (such as developmental disabilities, diabetes, heart disease, or seizures)	O	O	O
H. Rehabilitation, assistive technology, and other services for injured or disabled children	O	O	O
**Mental health, substance use, and self-harm**			
I. Assessment of risk factors and health behaviours (such as tobacco use, drug use, or physical inactivity)	O	O	O
J. Counselling on tobacco, alcohol, and other substance use	O	O	O
K. Short-term counselling or crisis intervention focused on mental health or situational concerns (such as grief or difficult transitions)	O	O	O
L. Management of common behavioural disorders in children (such as ADHD)	O	O	O
M. Management of emotional, anxiety, and depressive disorders	O	O	O
N. Management of eating disorders (such as **anorexia or bulimia**[17])	O	O	O
O. Stress management	O	O	O
P. Management of self-harm and/or suicide risk	O	O	O
Q. Management of **somatoform disorders**[18] and other psychosomatic conditions	O	O	O
R. Management of psychotic disorders	O	O	O
S. Management of harmful use of, dependence on, or withdrawal from a substance (such as alcohol or illicit drugs)	O	O	O

only

---

[14] Cholera – A bacterial disease causing acute diarrhoea and dehydration. Dengue – A viral disease spread through the bite of a certain type of infected mosquito. Dysentery – A bacterial disease causing inflammation of the intestines and bloody diarrhoea. Helminths – A parasitic worm that causes disease. Tuberculosis – A bacterial infection that mainly effects the lungs. Malaria – A serious and sometimes fatal disease caused by a parasite that infects a certain type of mosquito that feeds on humans.

[15] Anaemia – A condition marked by a lack of red blood cells or of hemoglobin in the blood.

[16] Obesity – A disorder involving excess body fat that increases the risk of other health problems.

[17] Anorexia – An eating disorder characterized by a very low body weight, an intense fear of gaining weight, and a distorted perception of weight. Bulimia - An eating disorder marked by binging followed by methods to avoid weight gain.

[18] Physical symptoms that suggest illness or injury, but which cannot be explained fully by a general medical condition or by the direct effect of a substance.

For peer review only - http://bmjopen.bmj.com/site/about/guidelines.xhtml

Health information management

2-15. Are the following types of health information recorded or kept on file in hard copy or electronic format for students in this school? *(Mark yes or no or I do not know for each type of health information.)*

	Yes	No	I do not know
A. Emergency contact information	O	O	O
B. Physical health history	O	O	O
C. Mental health history	O	O	O
D. Screening results	O	O	O
E. Vaccination history	O	O	O
F. Food allergy or other allergy information	O	O	O
G. Disabilities or special learning needs	O	O	O

2-16. Are the following types of data routinely collected from all students that attend this school? *(Mark yes or no or I do not know for each type of data.)*

	Yes	No	I do not know
A. Student health risk behaviours	O	O	O
B. Student injuries or illnesses that occur at school	O	O	O
C. Student use of school health services	O	O	O
D. Student perception of the quality of school health services	O	O	O
E. Reasons why students are absent from school	O	O	O

Note: If A, B, C, D, and E are all No or I do not know - Go to 2-18.

2-17. Does this school have an established process for routinely reviewing these data collected from students to help develop and implement school policies, programs, or activities?

A. Yes
B. No
C. I do not know

2-18. Does this school report any **notifiable diseases**[19] or infectious disease outbreaks that occur at school among students to the federal, state, or local ministry of health?

A. Yes
B. No
C. I do not know

School entry requirements

2-19. Does this school have a policy requiring students to be in compliance with the **National Immunization Schedule**[20] prior to enrollment in this school?

A. Yes
B. No
C. Not applicable. This country does not have a National Immunization Schedule.
D. I do not know

2-20. Does this school require students to have a preventive health check-up prior to enrollment in this school?

A. Yes
B. No
C. I do not know

---

[19] A notifiable disease is any disease that is required by law to be reported to government authorities.

[20] A National Immunization Schedule is a list of vaccinations, including the timing of all doses, which may be either recommended or compulsory, depending on the country

For peer review only - http://bmjopen.bmj.com/site/about/guidelines.xhtml

3. School Physical Environment

Water, sanitation, and hygiene

3-01. Which of the following statements **best** describes the **current** service level of this school's handwashing facilities for students?

A. Soap and water are available at handwashing facilities for students
B. Only water, but no soap, is available at handwashing facilities for students
C. No water is available at handwashing facilities for students
D. No handwashing facilities for students are available
E. I do not know

3-02. Which of the following statements **best** describes the **current** service level of this school's drinking water for students?

A. Drinking water for students is available from an **improved source**[21]
B. Drinking water for students comes from an improved source, but is not available
C. Drinking water for students is available from an **unimproved source**[22]
D. No drinking water for students is available
E. I do not know

3-03. Which of the following statements **best** describes the **current** service level of this school's sanitation facilities (such as toilets or latrines) for students?

A. Students have access to **improved sanitation facilities**[23] that are single sex, functional, and private
B. Students have access to improved sanitation facilities, but they are not single sex, they are not functional, or they are not private
C. Students only have access to **unimproved sanitation facilities**[24]
D. No sanitation facilities for students are available **(Go to 3-06)**
E. I do not know **(Go to 3-06)**

3-04. Does this school have sanitation facilities (such as toilets or latrines) that are accessible to students with physical disabilities?

A. Yes
B. No
C. I do not know

3-05. Are the school's sanitation facilities (such as toilets or latrines) usually cleaned daily when this school is in session?

A. Yes
B. No
C. I do not know

---

[21] Improved drinking water sources includes sources that, by nature of their construction or through active intervention, are protected from outside contamination, particularly fecal matter. These include piped water in a dwelling, plot, or yard; public standpipe; borehole; protected dug well; protected spring; and rainwater collection.
[22] Unimproved drinking water sources include unprotected dug well, unprotected spring, cart with small tank/drum, tanker truck, and surface water (river, dam, lake, pond, stream, canal, and irrigation channels).
[23] Improved sanitation facilities include a connection to a public sewer system, connection to a septic system, pour-flush latrine, and a ventilated improved pit latrine.
[24] Unimproved sanitation facilities include a public or shared latrine, an open pit latrine, and a bucket latrine.

For peer review only - http://bmjopen.bmj.com/site/about/guidelines.xhtml

3-06. Is garbage usually removed daily from the school premises when this school is in session?

A. Yes
B. No
C. I do not know

3-07. Does this school provide sanitary napkins or other menstrual supplies to students?

A. Yes, for free
B. Yes, for a fee
C. No
D. I do not know

Note: Ask this question only if females attend this school.

Injury prevention and safety

3-08. Are the following places or types of equipment routinely inspected for safety issues and hazards? *(Mark yes or no or I do not know for each place or type of equipment or mark not applicable (N/A) if this school does not have the place or type of equipment.)*

	Yes	No	I do not know	N/A
A. Playground equipment	O	O	O	O
B. Sports facilities	O	O	O	O
C. Fire extinguishers or other equipment to extinguish a fire (such as buckets of sand)	O	O	O	O
D. School structures and buildings	O	O	O	O
E. School grounds	O	O	O	O
F. Water source	O	O	O	O

3-09. Does this school have the following features to promote safety among students, including students with disabilities? *(Mark yes or no or I do not know for each feature.)*

	Yes	No	I do not know
A. Handrails on stairs	O	O	O
B. Ramps to facilitate change of level	O	O	O
C. Assistive devices (such as wheelchairs, wider doorways, specialized software, or adaptive switches or knobs)	O	O	O
D. Signage or paint to address vision impairments	O	O	O

Sun safety

3-10. Does this school use the following strategies to reduce sun exposure among students? *(Mark yes or no or I do not know for each strategy.)*

	Yes	No	I do not know
A. Schedule outdoor activities to avoid peak sun intensity	O	O	O
B. Provide shade areas	O	O	O
C. Encourage students to wear hats, other head coverings, or protective clothing when they are outside	O	O	O
D. Encourage students to wear sunscreen or other skin protection products when they are outside	O	O	O

For peer review only - http://bmjopen.bmj.com/site/about/guidelines.xhtml

Pest control

3-11. Are the school grounds where students play or exercise surrounded by a fence to keep out stray or roaming animals?

A. Yes
B. No
C. Not applicable. Stray or roaming animals are not a problem on school grounds
D. I do not know

3-12. Are procedures routinely implemented to control common pests (such as rodents, insects, or snakes) on school grounds?

A. Yes
B. No
C. I do not know

For peer review only

For peer review only - http://bmjopen.bmj.com/site/about/guidelines.xhtml

4. Food and Nutrition Services

4-01. Does this school provide **food and nutrition services**[25] to students?

A. Yes
B. No **(Go to 4-13)**
C. I do not know **(Go to 4-13)**

4-02. Which of the following statements **best** describes how school policy addresses food and nutrition services for students?

A. School policy provides a specific plan or guidelines for implementing food and nutrition services for students
B. Food and nutrition services are mentioned in a school policy, but there is no specific plan or guideline for implementing food and nutrition services for students
C. Food and nutrition services for students are not addressed in any school policy
D. I do not know

4-03. Does this school have someone officially responsible for managing or coordinating this school's food and nutrition services?

A. Yes
B. No
C. I do not know

4-04. Are the following meals or snacks routinely made available to students as part of this school's food and nutrition services? *(Mark yes or no or I do not know for each meal or snack.)*

	Yes	No	I do not know
A. Breakfast	O	O	O
B. Lunch	O	O	O
C. Evening meal	O	O	O
D. Snack(s)	O	O	O
E. Take-home rations	O	O	O

4-05. Which of the following statements **best** describes which students receive free or deeply discounted food as part of this school's food and nutrition services?

A. All students receive free or deeply discounted food
B. Some students receive free or deeply discounted food as determined by some kind of **social means testing**[26]*
or other quantitative criteria
C. No students receive free or deeply discounted food
D. I do not know

4-06. Does this school routinely conduct the following activities as part of its food and nutrition services for students? *(Mark yes or no or I do not know for each activity.)*

	Yes	No	I do not know
A. Collect suggestions from students, families, teachers, or other school staff on nutritious food and beverage preferences	O	O	O
B. Provide information to students or families on the nutrition and caloric content of foods and beverages	O	O	O
C. Work with local businesses to enhance the quality or variety of foods offered as part of this school's food and nutrition services	O	O	O

[25] The provision of food support either in the form of actual food or cash/vouchers/stipends for students to buy food available on school grounds.
[26] Assessment using set criteria to identify students who should receive school meals at no cost or at a deeply discounted price.

For peer review only - http://bmjopen.bmj.com/site/about/guidelines.xhtml

4-07. Are the following foods and beverages routinely made available to students through the school's food and nutrition services. *(Mark yes or no or I do not know for each food or beverage.)*

	Yes	No	I do not know
A. Fruit	O	O	O
B. Vegetables	O	O	O
C. Milk or milk products	O	O	O
D. Foods high in fiber such as whole grains, legumes, or nuts	O	O	O
E. Foods high in sugar including cookies, pastries, or other baked goods	O	O	O
F. Salty foods such as chips or crackers	O	O	O
G. Chocolate or other candy	O	O	O
H. Fried foods or other foods high in unhealthy fats	O	O	O
I. Sugar-sweetened carbonated soft drinks	O	O	O
J. Sports and energy drinks	O	O	O
K. 100% fruit or vegetable juices	O	O	O
L. Fruit or vegetable drinks that are not 100% juice	O	O	O
M. Sugar-sweetened flavoured milks	O	O	O
N. Sugar-sweetened teas, coffees, or flavoured waters	O	O	O
O. Water	O	O	O

Food preparation

4-08. Does this school have a policy describing how food served as part of the food and nutrition services should be prepared (in the school or elsewhere) to maximize its nutritional quality?

A. Yes
B. No **(Go to 4-13)**
C. I do not know **(Go to 4-13)**

4-09. Are the following nutrition objectives specifically addressed by this policy? *(Mark yes or no or I do not know for each nutrition objective.)*

	Yes	No	I do not know
A. Reduce saturated or trans-fat intake	O	O	O
B. Reduce salt intake	O	O	O
C. Reduce sugar intake	O	O	O
D. Increase fruit and vegetable consumption	O	O	O
E. Increase consumption of whole grains, legumes, and nuts	O	O	O
F. Increase **micronutrient**[27] intake	O	O	O

4-10. Are any foods served as part of this school's food and nutrition services fortified with **micronutrients**[27] (such as iron, iodine, zinc, or Vitamin A)?

A. Yes
B. No
C. I do not know

4-11. Are the foods that are part of this school's food and nutrition services prepared in the following locations? *(Mark yes or no or I do not know for each location.)*

	Yes	No	I do not know
A. On school grounds	O	O	O
B. Off-site in a centralized (not private) facility	O	O	O
C. Off-site in a private facility, such as a caterer	O	O	O

[27] Vitamins and minerals vital to healthy development, disease prevention, and well-being.

For peer review only - http://bmjopen.bmj.com/site/about/guidelines.xhtml

4-12. Does this school have a policy requiring food preparation staff (on school grounds or off-site) to follow safe food handling practices, such as **WHO's 5 Keys to Safer Food**[28]?

A. Yes
B. No
C. I do not know

Other foods and beverages available at school

4-13. May students purchase food or beverages from a vending machine, school store, canteen, or snack bar on school premises?

A. Yes
B. No **(Go to 4-17)**
C. I do not know **(Go to 4-17)**

4-14. Are healthy foods and beverages in the vending machine, school store, canteen, or snack bar on school premises priced lower than unhealthy options to encourage students to purchase the healthy ones?

A. Yes
B. No
C. I do not know

4-15. Does this school have a policy banning the sale of **sugar-sweetened beverages**[29] to students?

A. Yes
B. No
C. I do not know

4-16. Are the following foods and beverages routinely made available to students through the vending machines, stores, canteens, or snack bars on school premises. *(Mark yes or no or I do not know for each food or beverage.)*

	Yes	No	I do not know
A. Fruit	O	O	O
B. Vegetables	O	O	O
C. Milk or milk products	O	O	O
D. Foods high in fiber such as whole grains, legumes, or nuts	O	O	O
E. Foods high in sugar including cookies, pastries, or other baked goods	O	O	O
F. Salty foods such as chips or crackers	O	O	O
G. Chocolate or other candy	O	O	O
H. Fried foods or other foods high in unhealthy fats	O	O	O
I. Sugar-sweetened carbonated soft drinks	O	O	O
J. Sports and energy drinks	O	O	O
K. 100% fruit or vegetable juices	O	O	O
L. Fruit or vegetable drinks that are not 100% juice	O	O	O
M. Sugar-sweetened flavoured milks	O	O	O
N. Sugar-sweetened teas, coffees, or flavoured waters	O	O	O
O. Water	O	O	O

[28] WHO's 5 Keys to Safer Food are clean, separate raw and cooked, cook thoroughly, keep food at safe temperatures, and use safe water and raw materials.
[29] Sugar-sweetened beverages include carbonated soft drinks, sports drinks, energy drinks, 100% fruit juices, fruit drinks that are not 100% juice, sugar-sweetened flavoured milks, and sugar-sweetened teas, coffees, or flavoured waters.

For peer review only - http://bmjopen.bmj.com/site/about/guidelines.xhtml

Food and nutrition services environment

4-17. Does this school have a policy that students may bring food from home to eat during the school day?

A. Yes
B. No
C. I do not know

4-18. Does this school have a policy that students may bring water from home to drink during the school day?

A. Yes
B. No
C. I do not know

4-19. Where do most students **usually** eat meals during the school day?

A. In their regular classroom
B. In a dedicated eating space, such as a school cafeteria, dining hall, or other special room besides their regular classroom
C. Outside on school grounds
D. Somewhere else
E. Students do not eat meals during the school day
F. I do not know

4-20. Does this school have a policy prohibiting advertisements or promotions, including sponsorships, for candy, **sugar-sweetened beverages**[30], or other unhealthy foods and beverages on school premises?

A. Yes
B. No
C. I do not know

For peer review only

[30] Sugar-sweetened beverages include carbonated soft drinks, sports drinks, energy drinks, 100% fruit juices, fruit drinks that are not 100% juice, sugar-sweetened flavoured milks, and sugar-sweetened teas, coffees, or flavoured waters.

For peer review only - http://bmjopen.bmj.com/site/about/guidelines.xhtml

5. Health Education

5-01. Does this school teach **health education**[31]?

A. Yes
B. No **(Go to 6-01)**
C. I do not know **(Go to 6-01)**

5-02. Which of the following statements **best** describes how health education instruction occurs in this school?

A. Health education instruction occurs only in a regular classroom setting
B. Health education instruction occurs only through extra-curricular activities (such as after school, during lunch, or via clubs or school assemblies)
C. Health education instruction occurs both in a regular classroom setting and through extra-curricular activities
D. I do not know

5-03. Do students receive a grade for health education?

A. Yes
B. No
C. I do not know

5-04. Does this school have someone officially responsible for managing or coordinating this school's health education?

A. Yes
B. No
C. I do not know

5-05. Who provides **most** of the health education instruction in this school?

A. A health education teacher or specialist
B. A nurse or some other kind of health worker
C. Someone else
D. I do not know

5-06. Are the following materials provided to teachers in this school to guide health education instruction? *(Mark yes or no or I do not know for each material.)*

	**Yes**	**No**	**I do not know**
A. Learning outcomes or objectives	O	O	O
B. A planned progression of lesson plans or learning strategies and experiences	O	O	O
C. Teaching and learning resources and content	O	O	O
D. Assessment tools	O	O	O

[31] Any combination of learning experiences designed to help students improve their health by increasing their knowledge, influencing motivation, and improving health literacy.

For peer review only - http://bmjopen.bmj.com/site/about/guidelines.xhtml

5-07. Which of the following statements **best** describes how school policy addresses teaching **skills-based, participatory health education**[32]?

A. School policy provides a specific plan or guidance on teaching skills-based participatory health education
B. School policy mentions health education, but not teaching skills-based participatory health education
C. School policy does not mention health education
D. I do not know

5-08. Does this school routinely provide professional development opportunities for teachers to improve the quality of health education instruction?

A. Yes
B. No
C. I do not know

Content of health education

5-09. Are the following skills taught to students in this school to help them avoid or reduce health risks? *(Mark yes or no or I do not know for each skill.)*

	**Yes**	**No**	**I do not know**
A. How to access high quality health information, products, or services	O	O	O
B. Interpersonal communication	O	O	O
C. Decision-making	O	O	O
D. Problem-solving	O	O	O
E. Goal-setting	O	O	O
F. Refusal	O	O	O
F. Coping or stress management	O	O	O
G. Hand washing with soap	O	O	O
H. Tooth brushing with fluoride toothpaste	O	O	O
I. Using the internet and social media safely	O	O	O
J. Advocating for personal, family, or community health and wellbeing	O	O	O

[32] Skills-based, participatory health education includes strategies and techniques focused on skill development that encourage students to become actively involved in their learning process.

For peer review only - http://bmjopen.bmj.com/site/about/guidelines.xhtml

5-10. Are the following health topics taught to students in this school? *(Mark yes or no or I do not know for each topic.)*

	Yes	No	I do not know
A. Physical activity and fitness	O	O	O
B. Nutrition and dietary behaviour	O	O	O
C. **Sexual and reproductive health**[33]	O	O	O
D. HIV transmission, prevention, and treatment	O	O	O
E. Road safety	O	O	O
F. Violence and bullying prevention	O	O	O
G. Suicide prevention	O	O	O
H. Emotional and mental health	O	O	O
I. Tobacco and nicotine use prevention	O	O	O
J. Alcohol use prevention	O	O	O
K. Illicit drug use prevention	O	O	O
L. Infectious disease (such as the cold, flu, or COVID-19) prevention	O	O	O
M. Non-communicable disease (such as diabetes, cancer, or obesity) prevention	O	O	O
N. Oral health and prevention of oral disease	O	O	O
O. Healthy sleep	O	O	O
P. Personal hygiene, including handwashing	O	O	O
Q. Environmental health (such as the importance of clean air or water)	O	O	O
R. Sustainable development and consumption	O	O	O
S. Natural disaster preparedness and response	O	O	O
T. First aid	O	O	O
U. Immunizations (such as the HPV or COVID-19 vaccinations)	O	O	O
V. Safe sun exposure	O	O	O
W. Equity, inclusion, diversity, and human rights	O	O	O
X. Gender inequality and social norms	O	O	O

iew only

[33] Includes instruction on human growth and development, family life, reproduction, condoms and contraception, pregnancy, sexual behaviour, sexual abuse, and transmission and prevention of sexually transmitted infections.

For peer review only - http://bmjopen.bmj.com/site/about/guidelines.xhtml

6. Physical Education

Note: Ask this question if only males or only females attend this school.
6-01. Does this school teach **physical education**[34]?

A. Yes
B. No **(Go to 6-16)**
C. I do not know **(Go to 6-16)**

Note: Ask this question if both males and females attend this school.
6-02. Who is taught **physical education**[34] in this school?

A. All genders
B. Only males
C. Only females
D. No one **(Go to 6-16)**
E. I do not know **(Go to 6-16)**

6-03. Does this school provide adapted physical education for students with disabilities?

A. Yes
B. No
C. I do not know

6-04. Do students receive a grade for physical education?

A. Yes
B. No
C. I do not know

6-05. Can students in this school be excused from physical education for an extended period for the following reasons? *(Mark yes or no or I do not know for each reason.)*

	Yes	No	I do not know
A. Cultural	O	O	O
B. Gender	O	O	O
C. Academic achievement	O	O	O
D. Academic struggles or failure	O	O	O
E. Participation in other school activities	O	O	O
F. Poor physical health	O	O	O
G. Menstruation	O	O	O

Note: Ask G only if females attend this school.

6-06. Who provides **most** of the physical education instruction in this school?

A. A physical education teacher or specialist
B. A nurse or some other kind of health worker
C. Someone else
D. I do not know

[34] Class time spent teaching a physical education curriculum. Does not include instruction on physical activity topics in health education or any other subject.

For peer review only - http://bmjopen.bmj.com/site/about/guidelines.xhtml

6-07. Are the following materials provided to teachers in this school to guide physical education? *(Mark yes or no or I do not know for each type of material.)*

	Yes	No	I do not know
A. Learning outcomes or objectives	O	O	O
B. A planned progression of lesson plans or learning strategies and experiences	O	O	O
C. Teaching and learning resources and content	O	O	O
D. Assessment tools	O	O	O

6-08. Which of the following statements **best** describes how school policy addresses physical education for students?

A. School policy provides a specific plan or guidelines for implementing physical education
B. Physical education is mentioned in a school policy, but there is no specific plan or guideline for implementing physical education
C. Physical education is not addressed in any school policy
D. I do not know

6-09. Does this school routinely provide professional development opportunities for teachers to improve the quality of physical education instruction?

A. Yes
B. No
C. I do not know

Content of physical education

6-10. Are the following topics taught to students in this school? *(Mark yes or no or I do not know for each topic.)*

	Yes	No	I do not know
A. Movement concepts and skills (including motor skills such as walking or skipping and manipulative skills such as throwing, catching, or kicking)	O	O	O
B. The importance of life-long participation in physical activity	O	O	O
C. Developing an individualized physical activity plan, including frequency, intensity, and duration	O	O	O
D. The value and importance of fair play	O	O	O
E. Preventing injury during physical activity	O	O	O
F. The value of physical activity for health, enjoyment, challenge, self-expression, and/or social interaction	O	O	O
G. Strength training	O	O	O
H. Group or team sports or activities	O	O	O
I. Individual or paired sports or activities	O	O	O
J. The importance of endurance (aerobic) exercise for overall health and fitness	O	O	O
K. Flexibility or range of movement	O	O	O

Facilities and equipment

6-11. Does this school have a safe and clean **indoor** space for physical education class?

A. Yes
B. No
C. I do not know

For peer review only - http://bmjopen.bmj.com/site/about/guidelines.xhtml

6-12. Does this school have a safe and clean **outdoor** space for physical education class?

A. Yes
B. No **(Go to 6-16)**
C. I do not know **(Go to 6-16)**

6-13. Does this school allow community members to access this outdoor space for physical activity when school is not in session?

A. Yes
B. No
C. I do not know

6-14. Does this school provide a place where males and females can separately and privately change clothes before and after physical education?

A. Yes
B. No
C. I do not know

Note: Ask this question if both males and females attend this school.

6-15. Does this school have equipment (such as skipping ropes or balls) for use during physical education?

A. Yes
B. No
C. I do not know

Physical activity or recreation clubs and competitive sports

6-16. Does this school regularly provide recess or other physical activity breaks to students during the school day?

A. Yes
B. No
C. I do not know

6-17. Does this school offer **non-competitive physical activity or recreation clubs**[35] for students?

A. Yes
B. No
C. I do not know

6-18. Does this school offer school-sponsored sports teams that compete against teams from other schools?

A. Yes, for males only
B. Yes, for females only
C. Yes, for all genders
D. No
E. I do not know

Note: Ask this question if both males and females attend this school.

6-19. Does this school offer school-sponsored sports teams that compete against teams from other schools?

A. Yes
B. No
C. I do not know

Note: Ask this question if only males or only females attend this school.

[35] Any non-competitive physical activity program that is voluntary for students, in which students are given an equal opportunity to participate regardless of physical ability, and in which students have the opportunity to be involved in the planning, organization, and administration of the program, under the supervision of a qualified adult.

For peer review only - http://bmjopen.bmj.com/site/about/guidelines.xhtml

7. School Governance and Leadership

School health councils, committees, or teams

7-01. Does this school have an official council, committee, or team responsible for implementing health promoting school policies, programs, and activities?

A. Yes
B. No **(Go to 7-05)**
C. I do not know **(Go to 7-05)**

7-02. How often each year does this council, committee, or team routinely meet to address health promoting school policies, programs, and activities?

A. Weekly or more often
B. Monthly
C. Quarterly
D. Twice a year
E. Once a year or less often
F. I do not know

7-03. Are the following groups formally represented on this council, committee, or team? *(Mark yes or no or I do not know for each group.)*

	Yes	No	I do not know
A. School administrators	O	O	O
B. Teachers	O	O	O
C. Other school staff	O	O	O
D. Students	O	O	O
E. Parents, caregivers, or families	O	O	O
F. Government officials	O	O	O
G. Local businesses	O	O	O
H. Health workers (such as doctors or nurses)	O	O	O
I. Religious leaders	O	O	O

7-04. Does this council, committee, or team routinely conduct the following activities? *(Mark yes or no or I do not know for each activity.)*

	Yes	No	I do not know
A. Identify student health needs based on a review of relevant data	O	O	O
B. Recommend new or revised health promoting policies, programs, or activities to school administrators	O	O	O
C. Seek funding or leverage resources to support health promoting policies, programs, or activities for students or school staff	O	O	O
D. Communicate the importance of health promoting policies, programs, or activities to the school or community	O	O	O
E. Review health-related curricula or instructional materials	O	O	O

For peer review only - http://bmjopen.bmj.com/site/about/guidelines.xhtml

Community partnerships

7-05. Are the following groups regularly involved and engaged in **developing** health promoting school policies? *(Mark yes or no or I do not know for each group.)*

	Yes	No	I do not know
A. School administrators	O	O	O
B. Teachers	O	O	O
C. Other school staff	O	O	O
D. Students	O	O	O
E. Parents, caregivers, or families	O	O	O
F. Government officials	O	O	O
G. Local businesses	O	O	O
H. Health workers (such as doctors or nurses)	O	O	O
I. Religious leaders	O	O	O

7-06. Do the following groups regularly **receive copies of or information about** updated or new health promoting school policies? *(Mark yes or no or I do not know for each group.)*

	Yes	No	I do not know
A. School administrators	O	O	O
B. Teachers	O	O	O
C. Other school staff	O	O	O
D. Students	O	O	O
E. Parents, caregivers, or families	O	O	O
F. Government officials	O	O	O
G. Local businesses	O	O	O
H. Health workers (such as doctors or nurses)	O	O	O
I. Religious leaders	O	O	O

For peer review only - http://bmjopen.bmj.com/site/about/guidelines.xhtml

8. School Policies and Resources

8-01. Which of the following statements **best** describes how overall student health is promoted in school policy?

A. School policy provides a specific plan or guidance for promoting overall student health
B. Overall student health is mentioned in school policy, but without a specific plan or guidance on how to promote it
C. Overall student health is not addressed in any school policy
D. I do not know

8-02. Which of the following statements **best** describes the availability of resources in this school's budget to improve overall student health?

A. The school budget contains adequate resources to improve overall student health
B. The school budget contains some resources for improvement of overall student health, but not enough to do what is needed
C. The school budget does not contain any resources for improvement of overall student health
D. I do not know

8-03. Which of the following statements **best** describes how the health of teachers and other school staff is promoted in school policy?

A. School policy provides a specific plan or guidance for promoting the health of teachers and other school staff
B. The health of teachers and other school staff is mentioned in school policy, but without a specific plan or guidance on how to promote it
C. The health of teachers and other school staff is not addressed in any school policy
D. I do not know

8-04. Does this school regularly monitor, evaluate, or assess the quality of its health promoting policies, programs, or activities?

A. Yes
B. No
C. I do not know

The concept of a Health Promoting School embodies a whole-school approach to promoting health and educational attainment by capitalizing on the organizational potential of schools to foster the physical, social–emotional, and psychological conditions for health and positive educational outcomes. A Health Promoting School constantly strengthens its capacity as a safe and healthy setting for living, learning, and working. Other terms used to describe Health Promoting Schools are "comprehensive school health," "healthy school communities," and "school health education."

8-05. Does this school have a policy specifically about becoming or continuing to be a Health Promoting School?

A. Yes
B. No
C. I do not know

Overall curriculum

8-06. Does this school support **skills-based, participatory teaching methods**[36]?

A. Yes
B. No
C. I do not know

[36] Skills-based, participatory teaching methods are strategies and techniques focused on skill development that encourage students to become actively involved in their learning process.

For peer review only - http://bmjopen.bmj.com/site/about/guidelines.xhtml

8-07. Does this school allow teachers to make curriculum adaptations as appropriate to address the learning needs of students with disabilities?

A. Yes
B. No
C. I do not know

Professional development for teachers

8-08. Does this school routinely provide professional development opportunities for teachers on the following topics? *(Mark yes or no or I do not know for each topic.)*

	Yes	No	I do not know
A. The link between health and learning	O	O	O
B. Skills-based, participatory teaching methods	O	O	O
C. **Positive classroom management techniques**[37]	O	O	O
D. How to be a health promoting school	O	O	O
E. How to assess the health-related needs of students	O	O	O
F. How to deliver health-related content that best meets the needs of students	O	O	O
G. Standards for a healthy and safe learning environment	O	O	O
H. Child and adolescent development	O	O	O

Bullying and violence prevention

8-09. Does this school have a policy specifically prohibiting the following types of violence? *(Mark yes or no or I do not know for each type of violence.)*

	Yes	No	I do not know
A. Bullying among students	O	O	O
B. Fighting among students	O	O	O
C. Corporal punishment of students by teachers or other school staff	O	O	O
D. Physical, emotional, or sexual abuse of students by teachers or other school staff	O	O	O

8-10. Does this school have specific procedures in place for how a student can safely report any type of violence (such as bullying; fighting; corporal punishment; or physical, emotional, or sexual abuse)?

A. Yes
B. No
C. I do not know

8-11. Does this school have a policy describing how to respond when any type of violence (such as bullying; fighting; corporal punishment; or physical, emotional, or sexual abuse) occurs?

A. Yes
B. No
C. I do not know

[37] Positive classroom management techniques are focused on supporting and involving students to help them learn rather than focusing on bad behaviour.

For peer review only - http://bmjopen.bmj.com/site/about/guidelines.xhtml

8-12. Does this school collect data about incidences of violence (such as bullying; fighting; corporal punishment; or physical, emotional, or sexual abuse) including when, where, or how it occurred or who was involved?

A. Yes
B. No
C. I do not know

Tobacco use prevention

8-13. Does this school have a policy prohibiting use of at least some tobacco or nicotine products (such as cigarettes, other forms of smoked tobacco products, smokeless tobacco products, or electronic cigarettes) among the following groups on school premises? *(Mark yes or no or I do not know for each group.)*

	Yes	No	I do not know
A. Students	O	O	O
B. Teachers and other school staff	O	O	O
C. Visitors to the school	O	O	O

8-14. Does this school take the following actions to help reduce use of tobacco or nicotine products among students? *(Mark yes or no or I do not know for each action.)*

	Yes	No	I do not know
A. Implement policies on how to respond when students are caught using a tobacco or nicotine product on school premises	O	O	O
B. Post signs marking a tobacco-free school zone, that is, a specified distance from school grounds where tobacco use is prohibited	O	O	O
C. Prohibit advertising and promotion for tobacco and nicotine products on school premises	O	O	O

Alcohol use prevention

8-15. Does this school have a policy prohibiting use of alcohol among the following groups on school premises? *(Mark yes or no or I do not know for each group.)*

	Yes	No	I do not know
A. Students	O	O	O
B. Teachers and other school staff	O	O	O
C. Visitors to the school	O	O	O

8-16. Does this school take the following actions to help reduce use of alcohol among students? *(Mark yes or no or I do not know for each action.)*

	Yes	No	I do not know
A. Implement policies on how to respond when students are caught using alcohol on school premises	O	O	O
B. Post signs marking an alcohol-free school zone, that is, a specified distance from school grounds where alcohol use is prohibited	O	O	O
C. Prohibit advertising and promotion for alcohol products on school premises	O	O	O

For peer review only - http://bmjopen.bmj.com/site/about/guidelines.xhtml

Illicit drug use prevention

8-17. Does this school have a policy prohibiting use of illicit drugs among the following groups on school premises? *(Mark yes or no or I do not know for each group.)*

	Yes	No	I do not know
A. Students	O	O	O
B. Teachers and other school staff	O	O	O
C. Visitors to the school	O	O	O

8-18. Does this school take the following actions to help reduce use of illicit drugs among students? *(Mark yes or no or I do not know for each action.)*

	Yes	No	I do not know
A. Implement policies on how to respond when students are caught using illicit drugs on school premises	O	O	O
B. Post signs marking an illicit drug-free school zone, that is, a specified distance from school grounds where illicit drug use is prohibited	O	O	O

Crisis preparedness and emergency response

8-19. Does this school have a policy on crisis preparedness, response, and recovery from a natural disaster, conflict, pandemic, or other emergency situation?

A. Yes
B. No **(Go to 8-22)**
C. I do not know **(Go to 8-22)**

8-20. Does this policy ensure continuity for the following school services if the school building needs to be closed to students? *(Mark yes or no or I do not know for each service.)*

	Yes	No	I do not know
A. School health services	O	O	O
B. School food and nutrition services	O	O	O

8-21. Does this policy ensure continuity of instruction (possibly via remote learning) for the following subjects (or curricula) if the school building needs to be closed to students? *(Mark yes or no or I do not know for each subject.)*

	Yes	No	I do not know
A. Health education	O	O	O
B. Physical education	O	O	O
C. Other subjects	O	O	O

8-22. Does this school conduct regular emergency drills, such as fire or earthquake drills?

A. Yes
B. No
C. I do not know

8-23. Is this school officially designated to serve as a shelter for community members during or after a natural disaster?

A. Yes
B. No
C. I do not know

8-24. Does this school have measures in place in case of violence or conflict to help protect students and school staff (such as locked entry points, outsider identification processes, metal detectors, or security guards)?

A. Yes
B. No
C. I do not know

Eye health

8-25. Does this school have a policy requiring a minimum amount of outdoor activity daily to help prevent myopia or nearsightedness?

A. Yes
B. No
C. I do not know

Pregnant students

8-26. Does this school have a policy to allow pregnant students to attend school either in person or via remote learning?

A. Yes
B. No
C. I do not know

Note: Ask this question only if females attend this school.

review only

For peer review only - http://bmjopen.bmj.com/site/about/guidelines.xhtml

GLOBAL SCHOOL-BASED STUDENT HEALTH SURVEY
2021 Version

This survey is about your health and the things you do that may affect your health. Students like you all over your country are doing this survey. Students in many other countries around the world also are doing this survey. The information you give will be used to develop better health programs for young people like yourself.

DO NOT write your name on this survey or the answer sheet. The answers you give will be kept private. No one will know how you answer. Answer the questions based on what you really know or do. There are no right or wrong answers.

Completing the survey is voluntary. Your grade or mark in this class will not be affected whether or not you answer the questions. If you do not want to answer a question, just leave it blank.

Make sure to read every question. Fill in the circles on your answer sheet that match your answer. Use only the pencil you are given. When you are done, do what the person who is giving you the survey says to do.

Here is an example of how to fill in the circles:

Fill in the circles like this ● Not like this 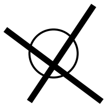 or 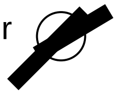

Survey

1. Do fish live in water?
   A. Yes
   B. No

Answer sheet

1. ● Ⓑ Ⓒ Ⓓ Ⓔ Ⓕ Ⓖ Ⓗ

Thank you very much for your help.

For peer review only

GSHS Core Questionnaire Respondent Demographics Module

1. How old are you?

   A. 11 years old or younger
   B. 12 years old
   C. 13 years old
   D. 14 years old
   E. 15 years old
   F. 16 years old
   G. 17 years old
   H. 18 years old or older

2. What is your sex?

   A. Male
   B. Female

3. In what grade/class/standard are you?
   COUNTRY SPECIFIC RESPONSE OPTIONS

   A. OPTION 1
   B. OPTION 2
   C. OPTION 3
   D. OPTION 4
   E. OPTION 5
   F. OPTION 6

For peer review only

GSHS Core Questionnaire Alcohol Use Module

The next 8 questions ask about drinking alcohol. This includes drinking COUNTRY SPECIFIC EXAMPLES. Drinking alcohol does not include drinking a few sips of wine for religious purposes. A "drink" is a glass of wine, a bottle of beer, a small glass of liquor or home brew, or a mixed drink.

1. How old were you when you had your first drink of alcohol other than a few sips?

   A. I have never had a drink of alcohol other than a few sips
   B. 7 years old or younger
   C. 8 or 9 years old
   D. 10 or 11 years old
   E. 12 or 13 years old
   F. 14 or 15 years old
   G. 16 or 17 years old
   H. 18 years old or older

2. During the past 30 days, on how many days did you have at least one drink containing alcohol?

   A. 0 days
   B. 1 or 2 days
   C. 3 to 5 days
   D. 6 to 9 days
   E. 10 to 19 days
   F. 20 to 29 days
   G. All 30 days

3. During the past 30 days, on the days you drank alcohol, how many drinks did you **usually** drink per day?

   A. I did not drink alcohol during the past 30 days
   B. Less than one drink
   C. 1 drink
   D. 2 drinks
   E. 3 drinks
   F. 4 drinks
   G. 5 or more drinks

4. During the past 30 days, what is the largest number of alcoholic drinks you had in a row,that is, within a couple of hours?

   A. I did not drink alcohol during the past 30 days
   B. 1 or 2 drinks
   C. 3 drinks
   D. 4 drinks
   E. 5 drinks
   F. 6 or 7 drinks
   G. 8 or 9 drinks
   H. 10 or more drinks

5. During the past 30 days, how did you **usually** get the alcohol you drank? SELECT ONLY ONE RESPONSE.

   A. I did not drink alcohol during the past 30 days
   B. I bought it in a store, shop, or from a street vendor
   C. I gave someone else money to buy it for me
   D. I got it from my friends
   E. I got it from my family
   F. I stole it or got it without permission
   G. I got it some other way

6. During your life, how many times have you got into trouble at home, work, or school or got into fights, as a result of drinking alcohol?

   A. 0 times
   B. 1 or 2 times
   C. 3 to 5 times
   D. 6 to 9 times
   E. 10 to 19 times
   F. 20 or more times

Staggering when walking, not being able to speak right, throwing up, and passing out are some signs of being really drunk.

7. During your life, how many times have you drank so much alcohol that you were really drunk?

   A. 0 times
   B. 1 or 2 times
   C. 3 to 5 times
   D. 6 to 9 times
   E. 10 to 19 times
   F. 20 or more times

For peer review only - http://bmjopen.bmj.com/site/about/guidelines.xhtml

GSHS Supplementary question on school attendance under the influence of alcohol

8. During the past 12 months, how many times did you attend school under the influence of alcohol?

   A. 0 times
   B. 1 or 2 times
   C. 3 to 5 times
   D. 6 to 9 times
   E. 10 to 19 times
   F. 20 to 39 times
   G. 40 or more times

For peer review only

For peer review only - http://bmjopen.bmj.com/site/about/guidelines.xhtml

GSHS Core Questionnaire Dietary Behaviours Module

The next 3 questions ask about your height, weight, and going hungry.

1. How tall are you without your shoes on? ON THE ANSWER SHEET, WRITE YOUR HEIGHT IN THE SHADED BOXES AT THE TOP OF THE GRID. THEN FILL IN THE CIRCLE BELOW EACH NUMBER.

Example

Height (cm)		
1	*5*	*3*
0	0	0
●	1	1
2	2	2
	3	●
	4	4
	●	5
	6	6
	7	7
	8	8
	9	9
	I do not know 9	

2. How much do you weigh without your shoes on? ON THE ANSWER SHEET, WRITE YOUR WEIGHT IN THE SHADED BOXES AT THE TOP OF THE GRID. THEN FILL IN THE CIRCLE BELOW EACH NUMBER.

Example

Weight (kg)		
0	*5*	*2*
●	0	0
1	1	1
2	2	●
	3	3
	4	4
	●	5
	6	6
	7	7
	8	8
	9	9
	I do not know 9	

3. During the past 30 days, how often did you go hungry because there was not enough food in your home?

   A. Never
   B. Rarely
   C. Sometimes
   D. Most of the time
   E. Always

The next 4 questions ask about what you might eat and drink.

4. During the past 7 days, how many times did you eat fruit, such as COUNTRY SPECIFIC EXAMPLES?

   A. I did not eat fruit during the past 7 days
   B. 1 to 3 times during the past 7 days
   C. 4 to 6 times during the past 7 days
   D. 1 time per day
   E. 2 times per day
   F. 3 times per day
   G. 4 or more times per day

5. During the past 7 days, how many times did you eat vegetables, such as COUNTRY SPECIFIC EXAMPLES?

   A. I did not eat vegetables during the past 7 days
   B. 1 to 3 times during the past 7 days
   C. 4 to 6 times during the past 7 days
   D. 1 time per day
   E. 2 times per day
   F. 3 times per day
   G. 4 or more times per day

6. During the past 7 days, how many times did you drink a can, bottle, or glass of a carbonated soft drink, such as COUNTRY SPECIFIC EXAMPLES? (Do **not** count diet soft drinks.)

   A. I did not drink carbonated soft drinks during the past 7 days
   B. 1 to 3 times during the past 7 days
   C. 4 to 6 times during the past 7 days
   D. 1 time per day
   E. 2 times per day
   F. 3 times per day
   G. 4 or more times per day

For peer review only

GSHS Core Questionnaire Dietary Behaviours Module

For this question, sugar-sweetened drinks include sports drinks (COUNTRY SPECIFIC EXAMPLES), energy drinks (COUNTRY SPECIFIC EXAMPLES), 100% fruit juices (COUNTRY SPECIFIC EXAMPLES), fruit drinks that are not 100% juice (COUNTRY SPECIFIC EXAMPLES), sugar-sweetened flavoured milks (COUNTRY SPECIFIC EXAMPLES), and sugar-sweetened teas, coffees, or flavoured waters.

For this question, DO NOT COUNT carbonated soft drinks measured in the previous question or diet or no calorie drinks.

7. During the past 7 days, how many times did you drink a can, bottle, or glass of a sugar-sweetened drink?

   A. I did not drink sugar-sweetened drinks during the past 7 days
   B. 1 to 3 times during the past 7 days
   C. 4 to 6 times during the past 7 days
   D. 1 time per day
   E. 2 times per day
   F. 3 times per day
   G. 4 or more times per day

For peer review only

For peer review only - http://bmjopen.bmj.com/site/about/guidelines.xhtml

GSHS Core Questionnaire Drug Use Module and supplementary question on school attendance under the influence of drugs

The next 4 questions ask about drug use. This includes using cannabis (also called marijuana), amphetamines, cocaine, inhalants, and heroin, and COUNTRY SPECIFIC EXAMPLES. Do not count tobacco and alcohol use.

1. How old were you when you first used drugs?

   A. I have never used drugs
   B. 7 years old or younger
   C. 8 or 9 years old
   D. 10 or 11 years old
   E. 12 or 13 years old
   F. 14 or 15 years old
   G. 16 or 17 years old
   H. 18 years old or older

2. During your life, how many times have you used cannabis (also called marijuana and COUNTRY SPECIFIC SLANG TERMS FOR CANNABIS)?

   A. 0 times
   B. 1 or 2 times
   C. 3 to 5 times
   D. 6 to 9 times
   E. 10 to 19 times
   F. 20 or more times

3. During the past 30 days, how many times did you use cannabis (also called marijuana and COUNTRY SPECIFIC SLANG TERMS FOR CANNABIS)?

   A. 0 times
   B. 1 or 2 times
   C. 3 to 5 times
   D. 6 to 9 times
   E. 10 to 19 times
   F. 20 or more times

4. During your life, how many times have you used amphetamines or methamphetamines (also called COUNTRY SPECIFIC SLANG TERMS FOR AMPHETAMINES AND METHAMPHETAMINES) for non-medical purposes?

   A. 0 times
   B. 1 or 2 times
   C. 3 to 5 times
   D. 6 to 9 times
   E. 10 to 19 times
   F. 20 or more times

5. During the past 12 months, how many times did you attend school under the influence of drugs, such as cannabis or cocaine?

   A. 0 times
   B. 1 or 2 times
   C. 3 to 5 times
   D. 6 to 9 times
   E. 10 to 19 times
   F. 20 or more times

For peer review only

For peer review only - http://bmjopen.bmj.com/site/about/guidelines.xhtml

GSHS Core Questionnaire Hygiene Module

The next 3 questions ask about your oral health.

1. During the past 30 days, how many times per day did you **usually** clean or brush your teeth?

   A. I did not clean or brush my teeth during the past 30 days
   B. Less than 1 time per day
   C. 1 time per day
   D. 2 times per day
   E. 3 or more times per day

2. During the past 30 days, did you **usually** use a toothpaste that contains fluoride when you cleaned or brushed your teeth?

   A. I did not clean or brush my teeth during the past 30 days
   B. Yes, I usually used a toothpaste that contains fluoride
   C. No, I did not usually use a toothpaste that contains fluoride
   D. I do not know if the toothpaste I usually used contains fluoride

3. During the past 30 days, did a problem with your mouth, teeth, or gums cause you to miss classes or school?

   A. Yes
   B. No

The next 3 questions ask about washing your hands.

4. During the past 30 days, how often did you wash your hands before eating?

   A. Never
   B. Rarely
   C. Sometimes
   D. Most of the time
   E. Always

5. During the past 30 days, how often did you wash your hands after using the toilet or latrine?

   A. Never
   B. Rarely
   C. Sometimes
   D. Most of the time
   E. Always

6. During the past 30 days, how often did you use soap when washing your hands?

   A. Never
   B. Rarely
   C. Sometimes
   D. Most of the time
   E. Always

For peer review only

GSHS Core Questionnaire Mental Health Module

The next 3 questions ask about your friendships and feelings.

1. How many close friends do you have?

   A. 0 friends
   B. 1 friend
   C. 2 friends
   D. 3 or more friends

2. During the past 12 months, how often did you feel lonely?

   A. Never
   B. Rarely
   C. Sometimes
   D. Most of the time
   E. Always

3. During the past 12 months, how often were you so worried about something that you could not sleep at night?

   A. Never
   B. Rarely
   C. Sometimes
   D. Most of the time
   E. Always

Sometimes people feel so depressed about the future that they may consider attempting suicide, that is, taking some action to end their own life. The next 3 questions ask about attempted suicide.

4. During the past 12 months, did you **seriously** consider attempting suicide?

   A. Yes
   B. No

5. During the past 12 months, did you make a plan about how you would attempt suicide?

   A. Yes
   B. No

6. During the past 12 months, how many times did you attempt suicide?

   A. 0 times
   B. 1 time
   C. 2 or 3 times
   D. 4 or 5 times
   E. 6 or more times

For peer review only

For peer review only - http://bmjopen.bmj.com/site/about/guidelines.xhtml

GSHS Core Questionnaire Physical Activity Module

The next 4 questions ask about physical activity. Physical activity is any activity that increases your heart rate and makes you breathe hard. Physical activity can be done in sports, playing with friends, walking to school, or in physical education class. Some examples of physical activity are running, fast walking, biking, dancing, football, and COUNTRY SPECIFIC EXAMPLES.

1. During the past 7 days, on how many days were you physically active for a total of at least 60 minutes per day? ADD UP ALL THE TIME YOU SPENT IN ANY KIND OF PHYSICAL ACTIVITY EACH DAY.

   A. 0 days
   B. 1 day
   C. 2 days
   D. 3 days
   E. 4 days
   F. 5 days
   G. 6 days
   H. 7 days

2. During the past 7 days, on how many days did you do exercises to strengthen or tone your muscles, such as push-ups, sit-ups, or weightlifting?

   A. 0 days
   B. 1 day
   C. 2 days
   D. 3 days
   E. 4 days
   F. 5 days
   G. 6 days
   H. 7 days

3. During the past 7 days, on how many days did you walk or ride a bicycle to or from school?

   A. 0 days
   B. 1 day
   C. 2 days
   D. 3 days
   E. 4 days
   F. 5 days
   G. 6 days
   H. 7 days

4. During this school year, on how many days did you go to physical education (PE) class each week?

   A. 0 days
   B. 1 day
   C. 2 days
   D. 3 days
   E. 4 days
   F. 5 or more days

The next question asks about the time you spend mostly sitting or lying down when you are not in school or doing homework.

5. How much time do you spend during a **typical or usual** day sitting or lying down doing such things as watching television, playing computer games, talking with friends, using your mobile phone, traveling in a motor vehicle, napping, or doing other activities sitting or lying down, such as COUNTRY SPECIFIC EXAMPLES? (Do **not** count time spent sleeping at night.)

   A. Less than 1 hour per day
   B. 1 to 2 hours per day
   C. 3 to 4 hours per day
   D. 5 to 6 hours per day
   E. 7 to 8 hours per day
   F. More than 8 hours per day

The next question asks about how much sleep you get.

6. On an average school night, how many hours of sleep do you get?

   A. 4 or less hours
   B. 5 hours
   C. 6 hours
   D. 7 hours
   E. 8 hours
   F. 9 hours
   G. 10 hours
   H. 11 or more hours

For peer review only - http://bmjopen.bmj.com/site/about/guidelines.xhtml

For peer review only

GSHS Core Questionnaire Protective Factors Module

The next 7 questions ask about your experiences at school and at home.

1. During the past 30 days, on how many days did you miss classes or school without permission?

   A. 0 days
   B. 1 or 2 days
   C. 3 to 5 days
   D. 6 to 9 days
   E. 10 or more days

2. During the past 30 days, how often were most of the students in your school kind and helpful?

   A. Never
   B. Rarely
   C. Sometimes
   D. Most of the time
   E. Always

3. During the past 30 days, how often were you able to talk to someone about difficult problems and worries?

   A. Never
   B. Rarely
   C. Sometimes
   D. Most of the time
   E. Always

4. During the past 30 days, how often did your parents or guardians understand your problems and worries?

   A. Never
   B. Rarely
   C. Sometimes
   D. Most of the time
   E. Always

5. During the past 30 days, how often did your parents or guardians check to see if your homework was done?

   A. Never
   B. Rarely
   C. Sometimes
   D. Most of the time
   E. Always

6. During the past 30 days, how often did your parents or guardians **really** know what you were doing with your free time?

   A. Never
   B. Rarely
   C. Sometimes
   D. Most of the time
   E. Always

7. During the past 30 days, how often did your parents or guardians go through your things without your approval?

   A. Never
   B. Rarely
   C. Sometimes
   D. Most of the time
   E. Always

For peer review only

For peer review only - http://bmjopen.bmj.com/site/about/guidelines.xhtml

GSHS Core Questionnaire Sexual Behaviours That Contribute to HIV Infection, Other STI, and Unintended Pregnancy Module

The next 5 questions ask about sexual intercourse.

1. Have you ever had sexual intercourse?

   A. Yes
   B. No

2. How old were you when you had sexual intercourse for the first time?

   A. I have never had sexual intercourse
   B. 11 years old or younger
   C. 12 years old
   D. 13 years old
   E. 14 years old
   F. 15 years old
   G. 16 or 17 years old
   H. 18 years old or older

3. During your life, with how many people have you had sexual intercourse?

   A. I have never had sexual intercourse
   B. 1 person
   C. 2 people
   D. 3 people
   E. 4 people
   F. 5 people
   G. 6 or more people

4. The **last time** you had sexual intercourse, did you or your partner use a condom or [COUNTRY SPECIFIC SLANG TERM FOR CONDOM]?

   A. I have never had sexual intercourse
   B. Yes
   C. No

5. The **last time** you had sexual intercourse, what **one** method did you or your partner use to **prevent pregnancy**? SELECT ONLY ONE RESPONSE.

   A. I have never had sexual intercourse
   B. No method was used to prevent pregnancy
   C. Birth control pills
   D. Condoms
   E. An IUD or implant
   F. A shot, patch, or birth control ring
   G. Withdrawal or some other method including COUNTRY SPECIFIC METHOD
   H. I do not know

For peer review only

For peer review only - http://bmjopen.bmj.com/site/about/guidelines.xhtml

GSHS Core Questionnaire Tobacco Use Module

The next 3 questions ask about cigarette use. Cigarettes include manufactured cigarettes, roll-your-own cigarettes, and kretek cigarettes, and COUNTRY SPECIFIC EXAMPLES.

1. Have you ever tried or experimented with cigarette smoking, even one or two puffs?

   A. Yes
   B. No

2. How old were you when you first tried smoking a cigarette?

   A. I have never tried smoking a cigarette
   B. 7 years old or younger
   C. 8 or 9 years old
   D. 10 or 11 years old
   E. 12 or 13 years old
   F. 14 or 15 years old
   G. 16 or 17 years old
   H. 18 years old or older

3. During the past 30 days, on how many days did you smoke cigarettes?

   A. 0 days
   B. 1 or 2 days
   C 3 to 5 days
   D. 6 to 9 days
   E. 10 to 19 days
   F. 20 to 29 days
   G. All 30 days

The next question asks about other forms of smoked tobacco products other than cigarettes. This includes pipes, cigars, mini cigars, cigarillos, waterpipes, hookah, shisha, narghile, hubble-bubble, bidis, and heated tobacco products (HTPs) and COUNTRY SPECIFIC EXAMPLES.

4. During the past 30 days, on how many days did you use any form of smoked tobacco products other than cigarettes?

   A. 0 days
   B. 1 or 2 days
   C 3 to 5 days
   D. 6 to 9 days
   E. 10 to 19 days
   F. 20 to 29 days
   G. All 30 days

The next question asks about smokeless tobacco products. This includes snuff, chewing tobacco, dip, betel quid with tobacco, and gutka and COUNTRY SPECIFIC EXAMPLES.

5. During the past 30 days, on how many days did you use any form of smokeless tobacco products?

   A. 0 days
   B. 1 or 2 days
   C 3 to 5 days
   D. 6 to 9 days
   E. 10 to 19 days
   F. 20 to 29 days
   G. All 30 days

The next question asks about electronic cigarettes. Electronic cigarettes, or e-cigarettes, are electronic devices that usually contain a nicotine-based liquid that is vaporized and inhaled. You may also know them as vape-pens, hookah-pens, electronic hookahs (e-hookahs), electronic cigars (e-cigars), electronic pipes (e-pipes), or e-vaporizers. Some look like cigarettes and others look like pens or small pipes. They are battery-powered devices that produce vapor instead of smoke. They do not contain tobacco.

6. During the past 30 days, on how many days did you use electronic cigarettes?

   A. 0 days
   B. 1 or 2 days
   C 3 to 5 days
   D. 6 to 9 days
   E. 10 to 19 days
   F. 20 to 29 days
   G. All 30 days

For peer review only

GSHS Core Questionnaire Violence and Unintentional Injury Module

The next 3 questions ask about <u>serious injuries</u> that happened to you. An injury is serious when it makes you miss at least one full day of usual activities (such as school, sports, or a job) or requires treatment by a doctor or nurse.

1. During the past 12 months, how many **times** were you seriously injured?

   A. 0 times
   B. 1 time
   C. 2 or 3 times
   D. 4 or 5 times
   E. 6 or 7 times
   F. 8 or 9 times
   G. 10 or 11 times
   H. 12 or more times

2. During the past 12 months, what was the **most serious injury** that happened to you?

   A. I was not seriously injured during the past 12 months
   B. I had a broken bone, a dislocated joint, or a broken or knocked out tooth
   C. I had a cut or stab wound
   D. I had a concussion or other head or neck injury, was knocked out, or could not breathe
   E. I had a gunshot wound
   F. I had a bad burn
   G. I was poisoned or took too much of a drug
   H. Something else happened to me

3. During the past 12 months, **what was the major cause** of the most serious injury that happened to you?

   A. I was not seriously injured during the past 12 months
   B. I was in a motor vehicle accident or hit by a motor vehicle
   C. I fell
   D. Something fell on me or hit me
   E. I was attacked or abused or was fighting with someone
   F. I was in a fire or too near a flame or something hot
   G. I inhaled or swallowed something bad for me
   H. Something else caused my injury

The next question asks about physical attacks. A physical attack occurs when one or more people hit or strike someone, or when one or more people hurt another person with a weapon (such as a stick, knife, or gun). It is not a physical attack when two students of about the same strength or power choose to fight each other.

4. During the past 12 months, how many times were you physically attacked?

   A. 0 times
   B. 1 time
   C. 2 or 3 times
   D. 4 or 5 times
   E. 6 or 7 times
   F. 8 or 9 times
   G. 10 or 11 times
   H. 12 or more times

The next question asks about physical fights. A physical fight occurs when two students of about the same strength or power choose to fight each other.

5. During the past 12 months, how many times were you in a physical fight?

   A. 0 times
   B. 1 time
   C. 2 or 3 times
   D. 4 or 5 times
   E. 6 or 7 times
   F. 8 or 9 times
   G. 10 or 11 times
   H. 12 or more times

For peer review only - http://bmjopen.bmj.com/site/about/guidelines.xhtml

GSHS Core Questionnaire Violence and Unintentional Injury Module

The next 3 questions ask about bullying. Bullying occurs when one or more students or other people about your age say or do hurtful or mean things. Bullying can occur when someone teases, threatens, ignores, spreads rumors about, calls someone a bad name, makes sexual remarks, or hits, shoves, or hurts another person over and over again. It is not bullying when two people of about the same strength or power argue or fight or tease each other in a friendly way.

6. During the past 12 months, were you bullied **on school property**?

   A. Yes
   B. No

7. During the past 12 months, were you bullied when you were **not on school property**?

   A. Yes
   B. No

Cyber bullying is a form of bullying using social media and other forms of online communication. Cyber bullying may happen on Instagram, Twitter, Snapchat, and Facebook, COUNTRY SPECIFIC EXAMPLES, and other social media platforms or through texting and email.

8. During the past 12 months, were you **cyber** bullied?

   A. Yes
   B. No

For peer review only - http://bmjopen.bmj.com/site/about/guidelines.xhtml

GSHS Supplementary questions on Physical Activity and the COVID-19 pandemic

The next question asks about physical activity. It is similar to but different than a question you were asked earlier. As a reminder, physical activity is any activity that increases your heart rate and makes you breathe hard. Physical activity can be done in sports, playing with friends, walking to school, or in physical education class. Some examples of physical activity are running, fast walking, biking, dancing, football, badminton, tennis, kabaddi, kho-kho, cricket, and swimming.

1. During the past 7 days, did you do at least **an average of** 60 minutes per day of physical activity across the 7 days?

   A. Yes
   B. No

Beginning in late 2019, the world experienced the coronavirus disease (COVID-19) pandemic. As part of the response to this pandemic, some schools and businesses were closed and some people were required to stay at home. Your community might still be experiencing the pandemic. The next question ask about the COVID-19 pandemic.

1. During the COVID-19 pandemic, did you attend school from home at least some of the time using a computer, mobile phone, or other electronic device?

   A. Yes
   B. No

For peer review only - http://bmjopen.bmj.com/site/about/guidelines.xhtml

Global School-Based Student Health Survey (GSHS) Parental/Guardian Notification Form

[Date]

**Introduction**	[Name of school] is participating in a study to help empower students to lead change using health data. Three other cities around the world are also participating. The study is sponsored by [name of agency]. The study has two main components.
**Global School-Based Student Health Survey (GSHS)**	The GSHS is a paper-and-pencil survey being given to a small number of students aged 13-17 across our city. It asks students about health behaviours and experiences. Students will not get any immediate benefit from taking part in the GSHS. However, the results of this survey will be used by some students, teachers, and local leaders to help develop a program to reduce student risk behaviors. Questions will be asked about the following topics: [list the selected core modules].
**Timeframe**	Students will be asked to fill out a paper questionnaire during regular class time and it will take about 30 to 45 minutes to complete.
**Physical activity study**	In addition to the GSHS, students from one class in each school will be asked to wear an activity monitor on their wrist for one week. The activity monitor will measure physical activity (sitting, standing, walking, running, and lying down). The activity monitor will not interfere with their daily activities. Students in activity monitor classrooms will be given detailed directions about how to wear the monitor. At the end of the week, they will be asked to complete the GSHS just like students in all other sampled classes.
**Student privacy**	Survey procedures have been designed to protect student privacy. Students do not put their name on the questionnaire or answer sheet. No school or student is ever mentioned by name in a report of the results. Some students may find some questions to be a little sensitive.
**Voluntary participation**	We would like all students to take part in the survey and physical activity study, **but both activities are voluntary**. No action will be taken against the school, you, or a student, if a student does not take part. Students can skip any question that they do not wish to answer, students may stop participating in the survey at any point without penalty, and students may refuse to wear the activity monitor.

For peer review only

**Questions**	If you have any questions, please contact [name and contact information of survey coordinator].<br>Thank you for your cooperation.

For peer review only

Global School-Based Student Health Survey (GSHS) Parental/Guardian Permission Form

**Introduction**	[Name of school] is participating in a study to help empower students to lead change using health data. Three other cities around the world are also participating. The study is sponsored by [name of agency]. The study has two main components.
**Global School-Based Student Health Survey (GSHS)**	The GSHS is a paper-and-pencil survey being given to a small number of students aged 13-17 across our city. It asks students about health behaviours and experiences. Students will not get any immediate benefit from taking part in the GSHS. However, the results of this survey will be used by some students, teachers, and local leaders to help develop a program to reduce student risk behaviors. Questions will be asked about the following topics: [list the selected core modules].
**Timeframe**	Students will be asked to fill out a paper questionnaire during regular class time and it will take about 30 to 45 minutes to complete.
**Physical activity study**	In addition to the GSHS, students from one class in each school will be asked to wear an activity monitor on their wrist for one week. The activity monitor will measure physical activity (sitting, standing, walking, running, and lying down). The activity monitor will not interfere with their daily activities. Students in activity monitor classrooms will be given detailed directions about how to wear the monitor. At the end of the week, they will be asked to complete the GSHS just like students in all other sampled classes.
**Student privacy**	Survey procedures have been designed to protect student privacy. Students do not put their name on the questionnaire or answer sheet. No school or student is ever mentioned by name in a report of the results. Some students may find some questions to be a little sensitive.
**Voluntary participation**	We would like all students to take part in the survey and physical activity study, **but both activities are voluntary**. No action will be taken against the school, you, or a student, if a student does not take part. Students can skip any question that they do not wish to answer, students may stop participating in the survey at any point without penalty, and students may refuse to wear the activity monitor.
**Questions**	If you have any questions, please contact [name and contact information of survey coordinator]. Thank you for your cooperation.

Please complete and return the following permission form by [date] if you do NOT want your student to take part in the study.

For peer review only - http://bmjopen.bmj.com/site/about/guidelines.xhtml

Student's name:____

I have read this form and know what the survey is about.
[ ] My student may **NOT** take part in this survey.

Parent/guardian signature:______________________________________ Date:___________________

For peer review only

Student Assent Form

**Introduction**	Your school is part of a study to help empower students to lead change using health data. This study is being done in three other cities around the world.<br><br>The study has several parts:<br>• a survey (called the Global School-based Student Health Survey – GSHS) where you will be asked to answer questions about your health;<br>• a physical activity study where some students will be asked to wear an activity monitor on their wrist;<br>• a workshop where some students will be asked to help come up with ideas to improve the health of all students in your school.
**Global School-Based Student Health Survey (GSHS)**	This survey is about your health and the things you do that may affect your health. Students like you from __ schools all over your city are doing this survey. Students in many other countries around the world have also done this survey before. The information you give will be used to develop better health programs for young people like yourself.<br><br>You will be asked to complete a paper questionnaire during regular class time. It will take about 30 to 45 minutes.<br><br>The answers you give will be kept private. No one will know how you answer. Please answer the questions based on what you really know or do. There are no right or wrong answers.
**Physical activity study**	Students from some classes also will be asked to wear an activity monitor on their wrist for one week. The activity monitor will measure physical activity (sitting, standing, walking, running, and lying down). The activity monitor will not interfere with anything you do. You will be given detailed directions about how to wear the monitor. At the end of the week, you will give the activity monitor back and then complete the GSHS just like students in other classes. The information collected with the activity monitor will be kept private and your name will not be recorded in the activity monitor.
**Workshop**	Some students will be asked to take part in data-to-action workshops. In these workshops, students, teachers, and local leaders will look at the study data and develop ideas on how the health of all students in your school could be improved.
**Your privacy**	The entire study has been designed to protect your privacy. There will not be any student, class, or school names on any questionnaire or answer sheet or recorded in the activity monitor. No school or student will be mentioned by name in a report of the results.

For peer review only

**Voluntary participation**	We would like all selected students to take part in the study, **but your participation is voluntary**. Your grade or mark in this class will not be affected whether or not you answer the questions or wear the activity monitor. If you do not want to answer a question, just leave it blank, you may stop participating in the study at any point without penalty, you may refuse to wear the activity monitor, and you do not have to volunteer for the data-to-action workshops.
**Questions**	If you have any questions, please contact [name and contact information of survey coordinator]. Thank you.

Please complete and return the following form by [date] if you do NOT want to take part in the study.

Your name:____

I have read this form and know what the study is about.
[ ] I do **NOT** want to take part in this study.

Student signature:_______________________________________ Date:____________________

For peer review only

Data to action workshop to improve adolescent health Parental/Guardian Notification Form

**Introduction**	[Name of school] has recently done a Global School-based Student Health Survey (GSHS) and a Global School Health Policies and Practices Survey (G-SHPSS) sponsored by [name of agency]. The GSHS collects information on health behaviours in 13-17 year old students, and the G-SHPPS on existing practices in schools. In the data to action workshop, the collected information will be used to plan interventions to improve the health of adolescents.
**Global AA-HA! workshop**	The data to action workshop will follow the Global Accelerated Action for the Health of Adolescents (Global AA-HA!) approach to improve young people's health. During the workshop, students, teachers and local authorities will explore information on student's behaviours and school practices together, identify where action is most needed, and set priorities for interventions to improve adolescent health. Students will not get any immediate benefit from taking part in the workshop. However, the results of the workshop will help students and other adolescents in the future.
**Timeframe**	Participation in the workshop will take about two hours per day on three consecutive days.
**Student privacy**	Workshop procedures have been designed to protect student privacy. No school or student will ever be mentioned by name in workshop documents.
**Voluntary participation**	**Participation in the workshop is voluntary**. Only parents/guardians of students that have put their name forward for voluntary participation in the workshop receive this permission form. If students wish, they may stop participating in the workshop at any point without penalty.
**Questions**	If you have any questions, please contact [name of local contact]. Thank you for your cooperation.

For peer review only

Data to action workshop to improve adolescent health Parental/Guardian Permission Form

**Introduction**	[Name of school] has recently done a Global School-based Student Health Survey (GSHS) and a Global School Health Policies and Practices Survey (G-SHPSS) sponsored by [name of agency]. The GSHS collects information on health behaviours in 13-17 year old students, and the G-SHPPS on existing practices in schools. In the data to action workshop, the collected information will be used to plan interventions to improve the health of adolescents.
**Global AA-HA! workshop**	The data to action workshop will follow the Global Accelerated Action for the Health of Adolescents (Global AA-HA!) approach to improve young people's health. During the workshop, students, teachers and local authorities will explore information on student's behaviours and school practices together, identify where action is most needed, and set priorities for interventions to improve adolescent health. Students will not get any immediate benefit from taking part in the workshop. However, the results of the workshop will help students and other adolescents in the future.
**Timeframe**	Participation in the workshop will take about two hours per day on three consecutive days.
**Student privacy**	Workshop procedures have been designed to protect student privacy. No school or student will ever be mentioned by name in workshop documents.
**Voluntary participation**	**Participation in the workshop is voluntary**. Only parents/guardians of students that have put their name forward for voluntary participation in the workshop receive this permission form. If students wish, they may stop participating in the workshop at any point without penalty.
**Questions**	If you have any questions, please contact [name of local contact]. Thank you for your cooperation.

Please complete and return the following permission form by [date] if you do NOT want your student to take part in the workshop.

Student's name:____

I have read this form and know what the workshop is about.

[ ] My student may **NOT** take part in this workshop.

Parent/guardian signature:_______________________________________ Date:____________________

For peer review only

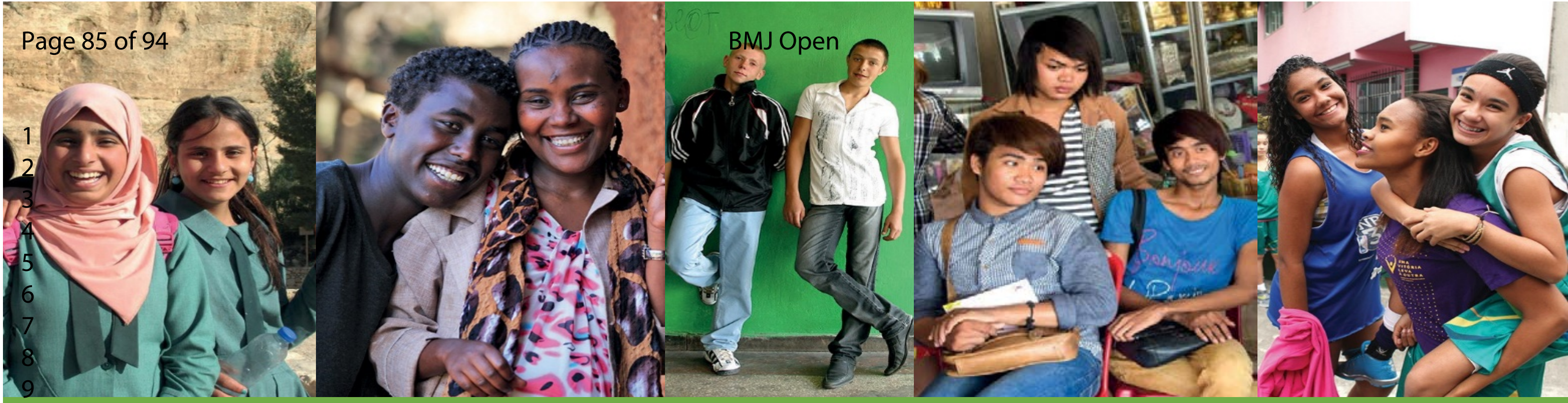

EMPOWERING ADOLESCENTS TO LEAD CHANGE USING HEALTH DATA

DATA-TO-ACTION WORKSHOP

Concept note

Background and Scope

Comprehensive local data on adolescent health are often lacking, particularly in lower-resource settings. Furthermore, there are knowledge gaps as to which interventions are effective to support healthy behaviours. To fill these gaps and generate new data and information, WHO across all three levels is conducting a study entitled "Empowering adolescents to lead change using health data", funded by Fondation Botnar.

The overarching aim of the study is to generate health information for students from cities in four low- and middle-income countries on which to plan, implement and subsequently evaluate a package of interventions to improve health outcomes. The study uses a cluster-randomized controlled trial design and is conducted in four cities of different world regions, namely Fez, Morocco; Jaipur, India; Saint Catherine Parish, Jamaica; and Sekondi-Takoradi, Ghana.

The study consists of several components: First, baseline assessments are conducted in randomly-selected schools of the four cities, using existing WHO tools: the Global School-based Student Health Survey (GSHS) to assess health behaviours and protective factors among students, and the Global School Health Policies and Practices Survey (G-SHPPS) to assess school policies. These data are being enhanced by photovoice (photos and drawings from the students). Second, in half of the schools (intervention arm), the GSHS and G-SHPPS baseline results will be used to plan and subsequently implement a package of interventions. This will be done following the approach of the Global Accelerated Action for the Health of Adolescents (AA-HA!) and based on the framework of the Global Standards for Health Promoting Schools. Third, follow-up surveys will be conducted two years after baseline to assess the differential change in health outcomes between the intervention and the control group.

The baseline surveys in the respective cities have been conducted from August 2022 – May 2023. The results from these baseline surveys will be used in a data-to-action workshop in each city, taking place a few months after completion of data collection.

For peer review only - http://bmjopen.bmj.com/site/about/guidelines.xhtml

Workshop objectives

The objectives of the data-to-action workshop are:

- Familiarize workshop participants with the study and the data;
- Identify adolescent health needs based on the collected data (needs assessment);
- Identify gaps in policies/programmes/practices (landscape analysis);
- Identify priority areas for action based on the needs assessment and landscape analysis;
- Develop a package of interventions to be implemented based on the identified priority areas for action;
- Develop an action plan with timelines and a plan to monitor implementation of the interventions.

Expected outcomes

Expected outcomes of the data-to-action workshop are:

- Workshop participants familiarized with the study and the data;
- Adolescent health needs identified;
- Gaps in policies/programmes/practices identified;
- Priority areas for action identified;
- Package of interventions to be implemented based on the identified priority areas for action identified;
- Action plan with timelines and a plan to monitor implementation of the interventions developed.

Methodology

The data-to-action workshop will be held over three days. The workshop will be held in the respective language spoken in each city and include plenary and group work sessions.

Participants

The data-to-action workshop will bring together:
- Education and health authority representatives;
- Community leaders;
- From the selected intervention schools in each city:
    - School focal points (including teachers, school administrators and school health persons);
    - Students;
    - Parents;
- Representatives of WHO and UNESCO;
- External partners.

For peer review only - http://bmjopen.bmj.com/site/about/guidelines.xhtml

Agenda

DAY 1

DAY 1		
Goal: Familiarize workshop participants with the study and the data and identify health needs		
**9.00 – 9.15**	Registration	
**9.15 - 9.30**	Welcome and opening remarks	Plenary
**9.30 – 10.00**	Workshop background and objectives<br>- Ice-breaking activity<br>- Participant's expected goals<br>- Expected outcomes of the workshop	Plenary
**10.00 – 10.30**	Introductory presentations<br>- Overview of the study and methods<br>- Overview of study implementation in the respective city	Plenary
**10.30 – 10.45**	Break	
**10.45 – 11.45**	Identifying health needs based on the GSHS results<br>- Presentation and review of GSHS results<br>- Group work	Plenary, Group work
**11.45 – 12.30**	Identifying health needs based on GSHS results<br>- Report back from group work	Group work
**12.30 – 13.30**	Lunch	
**13.30 – 15.00**	Identifying health needs based on photovoice<br>- Presentation and review of photovoice photos and drawing<br>- Group work and report back from group work	Plenary, Group work
**15.00 – 15.15**	Break	
**15.15 – 16.30**	Agreeing on health needs	Plenary

DAY 2

DAY 2		
Goal: Identify gaps in policies/programmes/practices and priority areas for action, draft interventions		
**9.00 – 9.20**	Recap of Day 1<br>- Review of expectations and of findings of Day 1	Plenary
**9.20 – 9.50**	Existing national & regional adolescent health programmes<br>- Presentations and short Q & A	Plenary
**9.50 – 10.30**	Landscape analysis: what policies/practices are in place and where are gaps? (Focus on health issues identified in Day 1)<br>- Presentation of G-SHPPS results<br>- Review of G-SHPPS results (group work)	Plenary, Group work
**10.30 – 10.45**	Break	
**10.45 – 11.30**	Identification of gaps in policies/programmes/practices	Plenary

For peer review only - http://bmjopen.bmj.com/site/about/guidelines.xhtml

	- Report back from group work	
**11.30 – 12.30**	Identification of priority areas for action	Plenary
**12.30 – 13.30**	Lunch	
**13.30 – 14.30**	Intervention planning:<br>- Review evidence-based interventions<br>- Identification of possible intervention ideas	Group work
**14.15 – 15.00**	Intervention planning:<br>- Report back from group work	Plenary
**15.00 – 15.15**	Break	
**15.15 – 16.30**	Revisiting and prioritizing possible interventions	Plenary

DAY 3

**DAY 3**		
Goal: Develop a package of interventions and an action plan with timelines and monitoring		
**9.00 – 9.20**	Recap of Day 2<br>- Review of expectations and of findings of Day 1	Plenary
**9.20 – 10.30**	Finalizing a package of prioritized interventions	Plenary
**10.30 – 10.45**	Break	
**10.45 – 12.30**	Developing a logic model:<br>- Presentation of a logic model<br>- Transferring results of Day 2 in a logic model	Plenary, Group work
**12.30 – 13.30**	Lunch	
**13.30 – 15.00**	Developing a unified action plan<br>- What can be measured and how?<br>- Roles and responsibilities	Group work, Plenary
**15.00 – 15.15**	Break	
**15.15 – 16.45**	Developing a unified action plan, cont.<br>- Timelines for implementation	Plenary
**16.45 – 17.00**	Feed-back, congratulations and closing remarks	Plenary

For peer review only - http://bmjopen.bmj.com/site/about/guidelines.xhtml

STANDARD PROTOCOL ITEMS: RECOMMENDATIONS FOR INTERVENTIONAL TRIALS

SPIRIT 2013 Checklist: Recommended items to address in a clinical trial protocol and related documents*

Section/item	ItemNo	Description	Page #/paragraph
**Administrative information**			
Title	1	Descriptive title identifying the study design, population, interventions, and, if applicable, trial acronym	Page 1
Trial registration	2a	Trial identifier and registry name. If not yet registered, name of intended registry	Page 5, Trial registration
	2b	All items from the World Health Organization Trial Registration Data Set	Page 5, Trial registration
Protocol version	3	Date and version identifier	Supplementary material: study protocol
Funding	4	Sources and types of financial, material, and other support	Page 24, Funding
Roles and responsibilities	5a	Names, affiliations, and roles of protocol contributors	Page 1-2; Page 23, Contributors
	5b	Name and contact information for the trial sponsor	Page 24, Funding
	5c	Role of study sponsor and funders, if any, in study design; collection, management, analysis, and interpretation of data; writing of the report; and the decision to submit the report for publication, including whether they will have ultimate authority over any of these activities	Page 24, Funding
	5d	Composition, roles, and responsibilities of the coordinating centre, steering committee, endpoint adjudication committee, data management team, and other individuals or groups overseeing the trial, if applicable (see Item 21a for data monitoring committee)	Page 24, Authors' contributions; Page 16-19, Interventions

For peer review only - http://bmjopen.bmj.com/site/about/guidelines.xhtml

**Introduction**			
Background and rationale	6a	Description of research question and justification for undertaking the trial, including summary of relevant studies (published and unpublished) examining benefits and harms for each intervention	Page 7-9, Introduction
	6b	Explanation for choice of comparators	Page 10, Study design
Objectives	7	Specific objectives or hypotheses	Page 9, Introduction, last paragraph
Trial design	8	Description of trial design including type of trial (eg, parallel group, crossover, factorial, single group), allocation ratio, and framework (eg, superiority, equivalence, noninferiority, exploratory)	Page 10, Study design
**Methods: Participants, interventions, and outcomes**			
Study setting	9	Description of study settings (eg, community clinic, academic hospital) and list of countries where data will be collected. Reference to where list of study sites can be obtained	Page 10, Study design
Eligibility criteria	10	Inclusion and exclusion criteria for participants. If applicable, eligibility criteria for study centres and individuals who will perform the interventions (eg, surgeons, psychotherapists)	Page 11, Study population and sampling
Interventions	11a	Interventions for each group with sufficient detail to allow replication, including how and when they will be administered	Page 16-19, Interventions
	11b	Criteria for discontinuing or modifying allocated interventions for a given trial participant (eg, drug dose change in response to harms, participant request, or improving/worsening disease)	N/A
	11c	Strategies to improve adherence to intervention protocols, and any procedures for monitoring adherence (eg, drug tablet return, laboratory tests)	Page 19, Monitoring and evaluation
	11d	Relevant concomitant care and interventions that are permitted or prohibited during the trial	N/A

For peer review only - http://bmjopen.bmj.com/site/about/guidelines.xhtml

Outcomes	12	Primary, secondary, and other outcomes, including the specific measurement variable (eg, systolic blood pressure), analysis metric (eg, change from baseline, final value, time to event), method of aggregation (eg, median, proportion), and time point for each outcome. Explanation of the clinical relevance of chosen efficacy and harm outcomes is strongly recommended	Page 12-15, Data collection and measures; Tables 1 and 2
Participant timeline	13	Time schedule of enrolment, interventions (including any run-ins and washouts), assessments, and visits for participants. A schematic diagram is highly recommended (see Figure)	Page 10, Study design; Figure 1
Sample size	14	Estimated number of participants needed to achieve study objectives and how it was determined, including clinical and statistical assumptions supporting any sample size calculations	Page 11-12, Study population and sampling
Recruitment	15	Strategies for achieving adequate participant enrolment to reach target sample size	Page 11-12, Study population and sampling; Page 12-14, Data collection and measures

Methods: Assignment of interventions (for controlled trials)

Allocation:

Sequence generation	16a	Method of generating the allocation sequence (eg, computer-generated random numbers), and list of any factors for stratification. To reduce predictability of a random sequence, details of any planned restriction (eg, blocking) should be provided in a separate document that is unavailable to those who enrol participants or assign interventions	Page 10, Study design
Allocation concealment mechanism	16b	Mechanism of implementing the allocation sequence (eg, central telephone; sequentially numbered, opaque, sealed envelopes), describing any steps to conceal the sequence until interventions are assigned	Page 10, Study design
Implementation	16c	Who will generate the allocation sequence, who will enrol participants, and who will assign participants to interventions	Page 10, Study design; Page 11-12, Study population and sampling

For peer review only - http://bmjopen.bmj.com/site/about/guidelines.xhtml

Blinding (masking)	17a	Who will be blinded after assignment to interventions (eg, trial participants, care providers, outcome assessors, data analysts), and how	Page 19-21, Data management and analysis plan
	17b	If blinded, circumstances under which unblinding is permissible, and procedure for revealing a participant's allocated intervention during the trial	N/A
**Methods: Data collection, management, and analysis**			
Data collection methods	18a	Plans for assessment and collection of outcome, baseline, and other trial data, including any related processes to promote data quality (eg, duplicate measurements, training of assessors) and a description of study instruments (eg, questionnaires, laboratory tests) along with their reliability and validity, if known. Reference to where data collection forms can be found, if not in the protocol	Page 12-15, Data collection and measures; Appendix
	18b	Plans to promote participant retention and complete follow-up, including list of any outcome data to be collected for participants who discontinue or deviate from intervention protocols	Page 19, Monitoring and evaluation
Data management	19	Plans for data entry, coding, security, and storage, including any related processes to promote data quality (eg, double data entry; range checks for data values). Reference to where details of data management procedures can be found, if not in the protocol	Page 19-21, Data management and analysis plan
Statistical methods	20a	Statistical methods for analysing primary and secondary outcomes. Reference to where other details of the statistical analysis plan can be found, if not in the protocol	Page 19-21, Data management and analysis plan
	20b	Methods for any additional analyses (eg, subgroup and adjusted analyses)	Page 19-21, Data management and analysis plan
	20c	Definition of analysis population relating to protocol non-adherence (eg, as randomised analysis), and any statistical methods to handle missing data (eg, multiple imputation)	Page 19-21, Data management and analysis plan

**Methods: Monitoring**			
Data monitoring	21a	Composition of data monitoring committee (DMC); summary of its role and reporting structure; statement of whether it is independent from the sponsor and competing interests; and reference to where further details about its charter can be found, if not in the protocol. Alternatively, an explanation of why a DMC is not needed	Page 19-21, Data management and analysis plan; Reference to standard procedures as per GSHS manual; Page 24, Funding
	21b	Description of any interim analyses and stopping guidelines, including who will have access to these interim results and make the final decision to terminate the trial	Page 16-19, Interventions
Harms	22	Plans for collecting, assessing, reporting, and managing solicited and spontaneously reported adverse events and other unintended effects of trial interventions or trial conduct	Page 19, Monitoring and evaluation
Auditing	23	Frequency and procedures for auditing trial conduct, if any, and whether the process will be independent from investigators and the sponsor	Page 19, Monitoring and evaluation; Page 24, Funding
**Ethics and dissemination**			
Research ethics approval	24	Plans for seeking research ethics committee/institutional review board (REC/IRB) approval	Page 22-23, Ethics and dissemination
Protocol amendments	25	Plans for communicating important protocol modifications (eg, changes to eligibility criteria, outcomes, analyses) to relevant parties (eg, investigators, REC/IRBs, trial participants, trial registries, journals, regulators)	Page 22-23, Ethics and dissemination

For peer review only - http://bmjopen.bmj.com/site/about/guidelines.xhtml

Consent or assent	26a	Who will obtain informed consent or assent from potential trial participants or authorised surrogates, and how (see Item 32)	Page 11-12, Study population and sampling; Page 15-16, Physical activity validation study; Page 22-23, Ethics and dissemination
	26b	Additional consent provisions for collection and use of participant data and biological specimens in ancillary studies, if applicable	N/A
Confidentiality	27	How personal information about potential and enrolled participants will be collected, shared, and maintained in order to protect confidentiality before, during, and after the trial	Page 19-21, Data management and analysis plan
Declaration of interests	28	Financial and other competing interests for principal investigators for the overall trial and each study site	Page 24, Competing interests
Access to data	29	Statement of who will have access to the final trial dataset, and disclosure of contractual agreements that limit such access for investigators	Page 22-23, Ethics and dissemination
Ancillary and post-trial care	30	Provisions, if any, for ancillary and post-trial care, and for compensation to those who suffer harm from trial participation	N/A
Dissemination policy	31a	Plans for investigators and sponsor to communicate trial results to participants, healthcare professionals, the public, and other relevant groups (eg, via publication, reporting in results databases, or other data sharing arrangements), including any publication restrictions	Page 22-23, Ethics and dissemination
	31b	Authorship eligibility guidelines and any intended use of professional writers	Page 23, Contributors
	31c	Plans, if any, for granting public access to the full protocol, participant-level dataset, and statistical code	Page 22-23, Ethics and dissemination

For peer review only - http://bmjopen.bmj.com/site/about/guidelines.xhtml

Appendices

Informed consent materials	32	Model consent form and other related documentation given to participants and authorised surrogates	Appendix
Biological specimens	33	Plans for collection, laboratory evaluation, and storage of biological specimens for genetic or molecular analysis in the current trial and for future use in ancillary studies, if applicable	N/A

*It is strongly recommended that this checklist be read in conjunction with the SPIRIT 2013 Explanation & Elaboration for important clarification on the items. Amendments to the protocol should be tracked and dated. The SPIRIT checklist is copyrighted by the SPIRIT Group under the Creative Commons "Attribution-NonCommercial-NoDerivs 3.0 Unported" license.

For peer review only
